# Enantio-Complementary Synthesis of 2-Substituted Pyrrolidines and Piperidines via Transaminase-Triggered Cyclizations

**DOI:** 10.1021/jacsau.3c00103

**Published:** 2023-05-12

**Authors:** Christian M. Heckmann, Caroline E. Paul

**Affiliations:** Biocatalysis section, Department of Biotechnology, Delft University of Technology, Van der Maasweg 9, 2629 HZ Delft, The Netherlands

**Keywords:** asymmetric synthesis, biocatalysis, chiral amines, enzyme, *N*-heterocycles

## Abstract

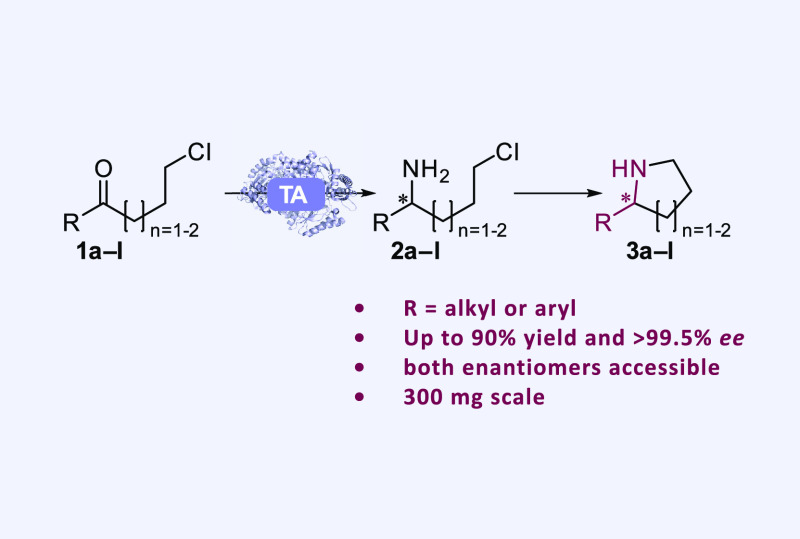

Chiral *N*-heterocycles are a common motif in many active pharmaceutical ingredients; however, their synthesis often relies on the use of heavy metals. In recent years, several biocatalytic approaches have emerged to reach enantiopurity. Here, we describe the asymmetric synthesis of 2-substituted pyrrolidines and piperidines, starting from commercially available ω-chloroketones by using transaminases, which has not yet been comprehensively studied. Analytical yields of up to 90% and enantiomeric excesses of up to >99.5% for each enantiomer were achieved, which has not previously been shown for bulky substituents. This biocatalytic approach was applied to synthesize (*R*)-2-(*p*-chlorophenyl)pyrrolidine on a 300 mg scale, affording 84% isolated yield, with >99.5% *ee*.

## Introduction

**Figure 1:**
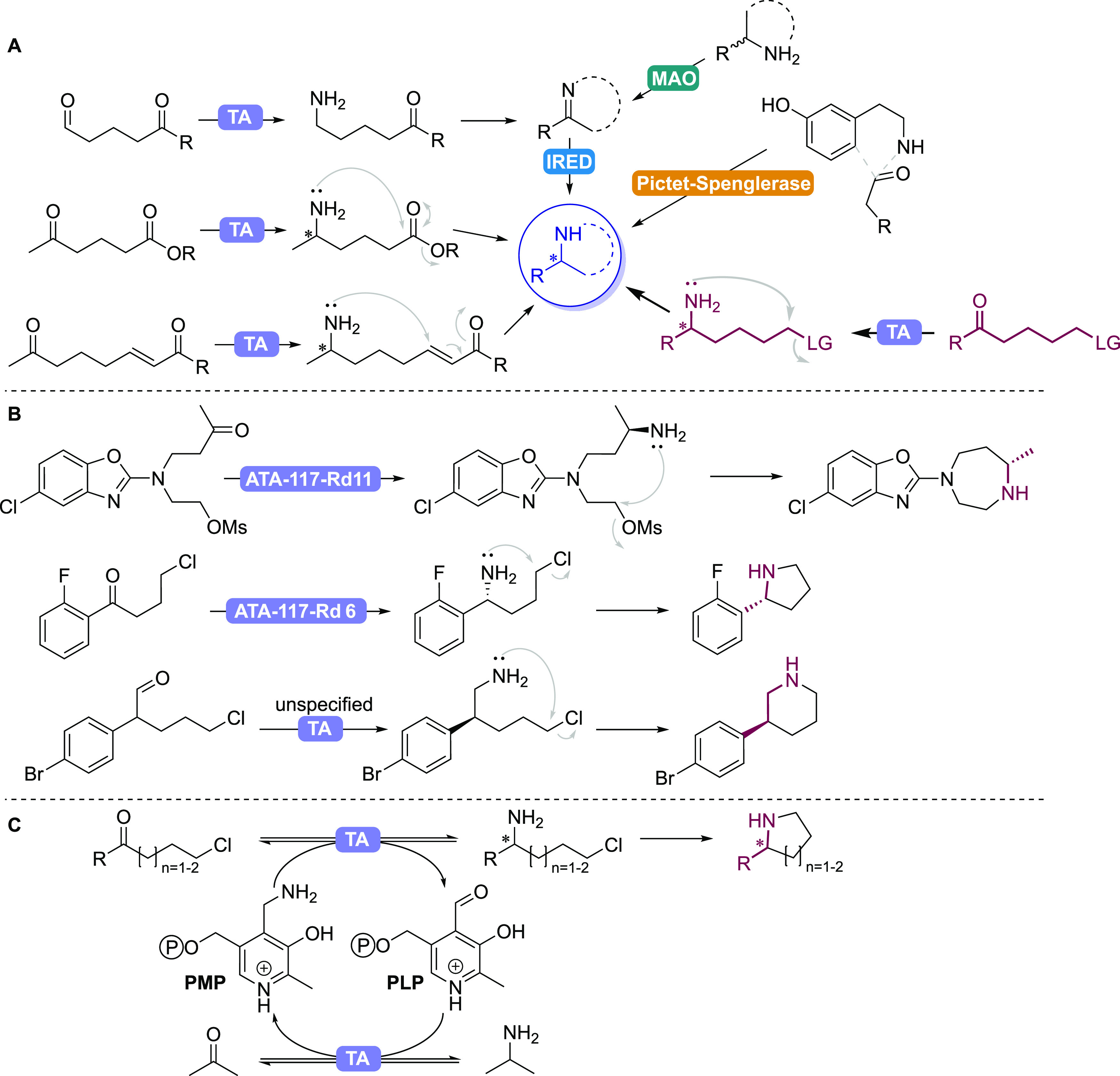
(A) Biocatalytic strategies for the synthesis of chiral cyclic amines. (B) Literature examples of the transamination of carbonyl substrates bearing a terminal leaving group, followed by cyclization. (C) Proposed synthesis of 2-substituted chiral pyrrolidines and piperidines starting from commercially available ω-chloroketones.

Chiral amines are important building blocks of many active pharmaceutical ingredients (APIs) and agrochemicals;^1,2^ however, their synthesis by nonenzymatic means usually requires stoichiometric amounts of chiral auxiliaries or the use of chiral rare metal catalysts.^3^ On the other hand, an increasingly large portfolio of enzymes has become available for the synthesis of chiral amines, such as transaminases,^4^ imine reductases (including reductive aminases),^5^ and amine dehydrogenases.^6^ These enzymes have found applications in the synthesis of APIs such as antidiabetic drug sitagliptin,^7^ LSD1 inhibitor GSK2879552,^8^ JAK1 inhibitor abrocitinib,^9^ BTK inhibitor nemtabrutinib,^10^ and CDK2/4/6 inhibitor PF-06873600,^11^ improving the efficiency and sustainability of these processes on up to >100 kg scales.^12^ Nitrogen-containing heterocycles represent a privileged structure in many APIs,^13^ and thus chiral cyclic amines are especially important building blocks. Several biocatalytic routes toward this moiety have been reported (Figure 1A);^14−23^ yet, an attractive strategy, biocatalytic reductive amination of a ketone bearing a leaving group, followed by spontaneous cyclization, remains to be explored (Figure 1B).^7,24,25^ Only three examples have been reported in the literature, two of which produce the (*R*)-enantiomer in the 2-position.^7,24^ The third example generates a 3-substituted piperidine, but was abandoned during development due to the instability of the aldehyde starting material, and no data on conversions or yield were reported.^25^ Transaminases (TAs) are pyridoxal-5′-phosphate (PLP)-dependent enzymes catalyzing the transfer of an amino group from a sacrificial amine donor to a prochiral ketone substrate. Here, we describe their application in the stereoselective synthesis of 2-substituted chiral pyrrolidines and piperidines starting from commercially available ω-chloroketones, employing isopropylamine (IPA) as the amine donor (Figure 1C). Access to both enantiomers is reported for the first time, and the effects of both methyl and electron-rich and -deficient phenyl substituents are explored.

## Results and Discussion

A panel of 10 TAs was selected, composed of five (*S*)- and five (*R*)-selective enzymes, all of which have previously been described. For the (*S*)-selective TAs, *Cv*STA^26^ from *Chromobacterium violaceum*, and HEwT (wt^27^ and W56G mutant^28^) from *Halomonas elongata*, as well as two TAs that have been engineered to accept bulky–bulky ketones, 3FCR-4M^29^ from *Ruegeria* sp. TM1040, and *Pj*STA-R6-8^30^ from *Pseudomonas jessenii* were selected. For the (*R*)-selective TAs, *At*RTA^31^ from *Aspergillus terreus*, *Ts*RTA^32^ from *Thermomyces stellatus*, ATA-117 from *Arthrobacter* sp., as well as its evolved variants for the synthesis of sitagliptin,^7^ ATA-117-Rd6, and ATA-117-Rd11 were chosen. All TAs were produced by recombinant expression in *Escherichia coli* BL21(DE3) and used as lyophilized cell-free extracts (CFEs, SI Figure S1).

**Figure 2:**
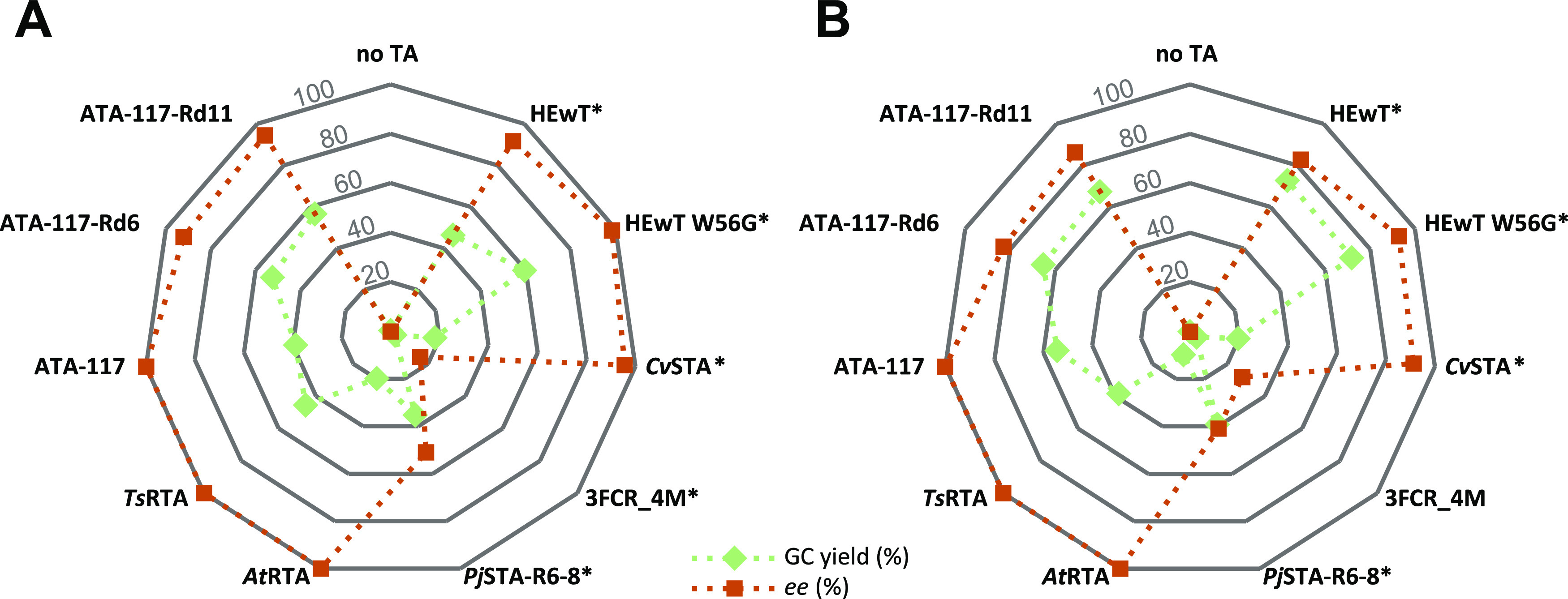
GC yields (determined using a calibration curve of the products) and *ee*s for the TA-catalyzed synthesis of (A) 2-methylpyrrolidine **3a** and (B) 2-methylpiperidine **3b**. Enzymes labeled with an asterisk produce the (*S*)-enantiomer, while those without produce the (*R*)-enantiomer. Conditions: TA (10 mg/mL), **1a-b** (50 mM), PLP (1 mM), IPA (0.5), DMSO (5% v/v), KP_*i*_-buffer (100 mM), pH 8, 30 °C, 700 rpm, final volume 0.5 mL. Reaction time: 22 h (**1a**; except *Pj*STA-R6-8: 24 h) or 24 h followed by the addition of NaOH (100 μL, 10 M) and further incubation for 3h (**1b**). Data are the average of duplicates.

**Figure 3:**
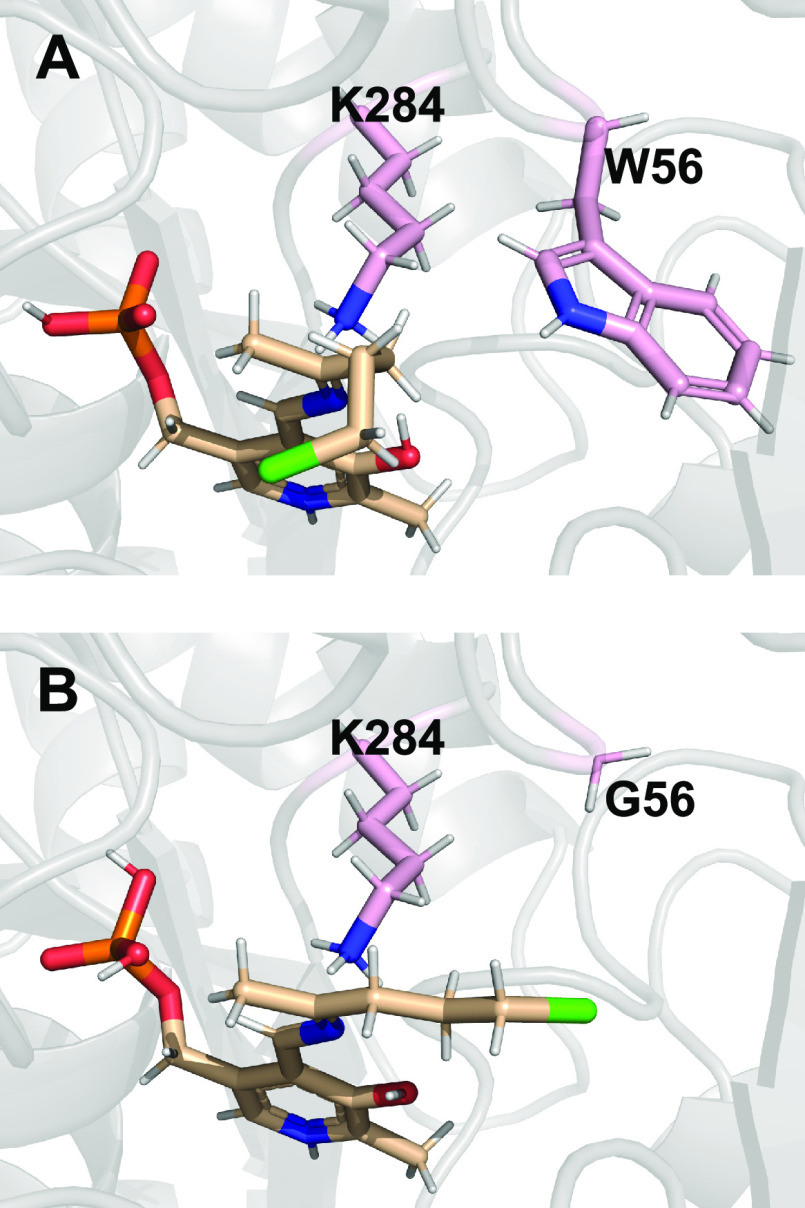
Docked quinonoid intermediate of 5-chloropentan-2-one **1a**. (A) HEwT wt, (B) HEwT W56G. Docking was carried out with the dock_run.mcr macro in YASARA 20.12.24; the figure was generated using Open Source PyMOL 2.5.0.

**Scheme 1:**
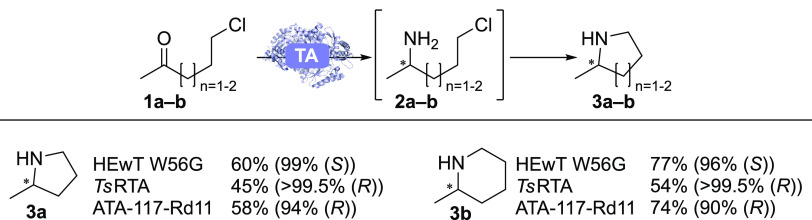
GC Yields (Determined Using a Calibration Curve of the Products) and *ee*s (in Parentheses) for the Best-Performing Variants in the TA-Catalyzed Synthesis of **3a** and **3b** Conditions: TA (10 mg/mL), **1a-b** (50 mM), PLP (1 mM), IPA (1 M or 0.5 M (*Ts*RTA and ATA-117)), DMSO (5% v/v), KP_*i*_-buffer (100 mM), pH 8, 30 °C, 700 rpm, final volume 0.5 mL Reaction time: 22 h (**1a**) or 24 h followed by the addition of NaOH (100 μL, 10 M) and further incubation for 3h (**1b**). Data are the average of duplicates.

Initial reactions were set up using 5-chloropentan-2-one **1a** and 6-chlorohexan-2-one **1b** as substrates (Figure 2, SI Figures S2,3), with the best-performing variants summarized in Scheme 1. HEwT W56G showed the highest yields (determined by gas chromatography (GC) with a calibration curve of the product) and enantioselectivity among the (*S*)-selective TAs. Removing the bulky tryptophan side chain alleviates steric clash with the chloro-alkyl chain, allowing for the chloro-alkyl chain to extend into the binding pocket, which one may speculate leads to a more productive positioning (Figure 3), although more detailed modeling would be required to confirm this hypothesis. A similar effect on enantioselectivity has previously been reported for HEwT mutants bearing a W56C mutation with *p*-substituted acetophenones.^33^ For the (*R*)-selective TAs, a trade-off between GC yield and enantioselectivity was observed. The wild-type TAs showed essentially complete enantioselectivities in almost all cases. On the other hand, the engineered variants of ATA-117 showed higher analytical yields of the product but with reduced *ee*s. The improved conversions are likely due to enhanced acceptance of IPA as the amine donor (SI Figures S2,3 see the effect of 1 vs 0.5 M IPA on ATA-117 and the engineered variants), for which the variants were optimized during the evolutionary campaign, as well as their increased stability.^7^ The variants having been engineered to accept bulky–bulky ketones, reduced enantioselectivity with the small–bulky ketones used here was expected. While cyclization to the pyrrolidine was spontaneous under the reaction conditions (the chloro-amine was never detected), cyclization to the piperidine required incubation with sodium hydroxide for 3 h, due to the additional degree of freedom conferred by the extra methylene. Significant levels of nonenzymatic hydrolysis of **1a** were observed during the biocatalytic reaction, whereas **1b** was stable and only degraded during the incubation with excess sodium hydroxide (SI Figure S4).

**Figure 4:**
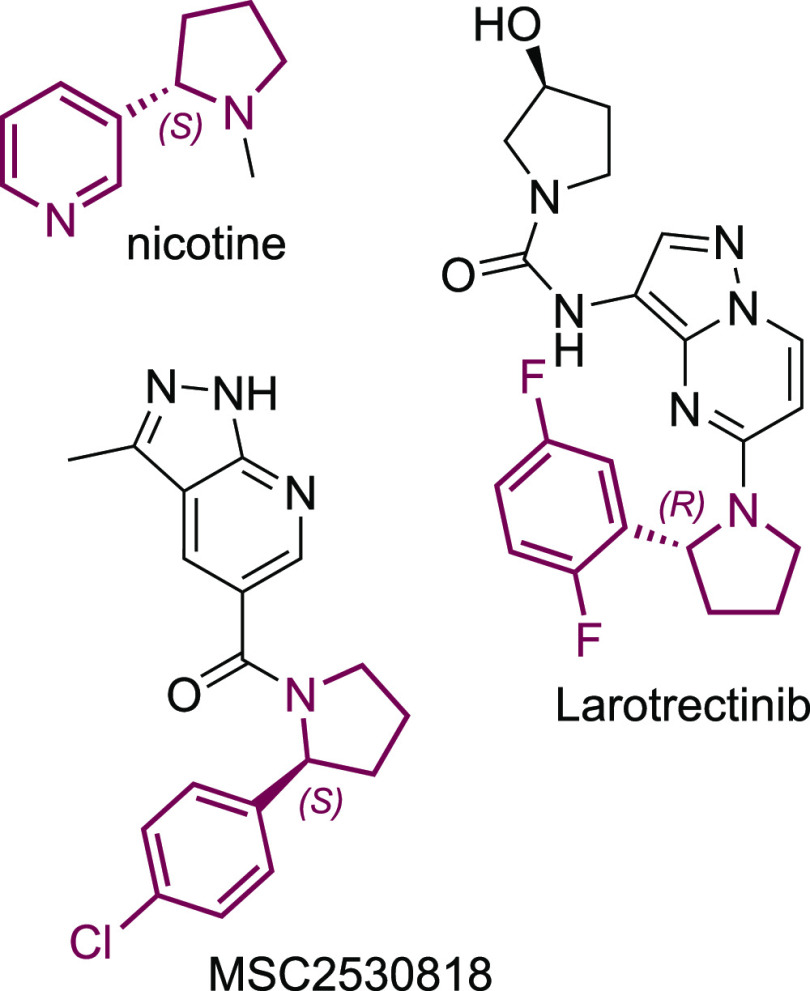
Structures of bioactive molecules containing 2-arylpyrrolidines.

**Figure 5:**
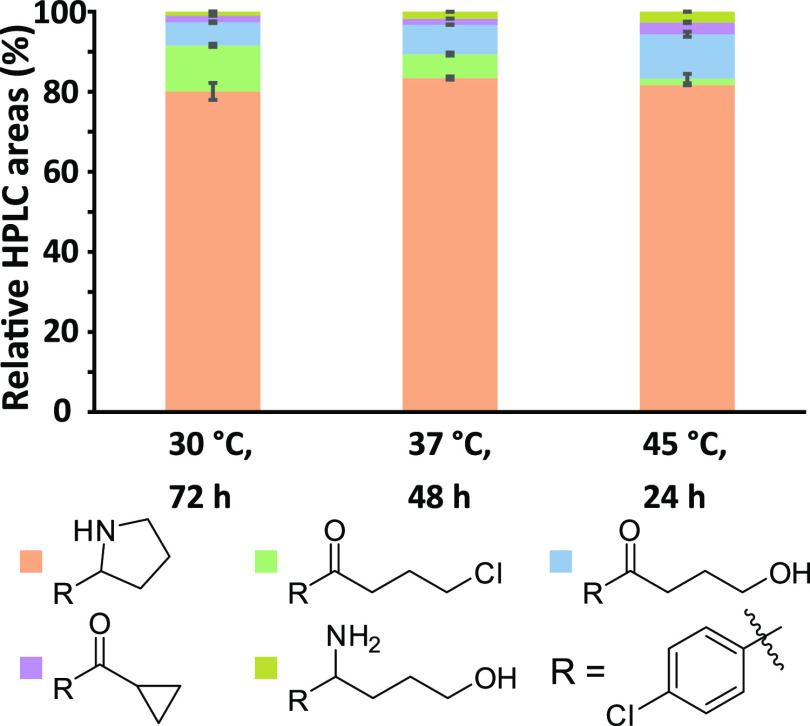
Relative HPLC areas of TA-catalyzed reactions with **1f** as substrate, using varying temperatures (30, 37, and 45 °C). Conditions: ATA-117-Rd6 (10 mg/mL), **1f** (50 mM), PLP (1 mM), IPA (1 M), DMSO (20% v/v), KP_*i*_-buffer (100 mM), pH 8, 700 rpm, final volume 0.5 mL. Data are the average of duplicates; error bars represent standard errors.

**Scheme 2:**
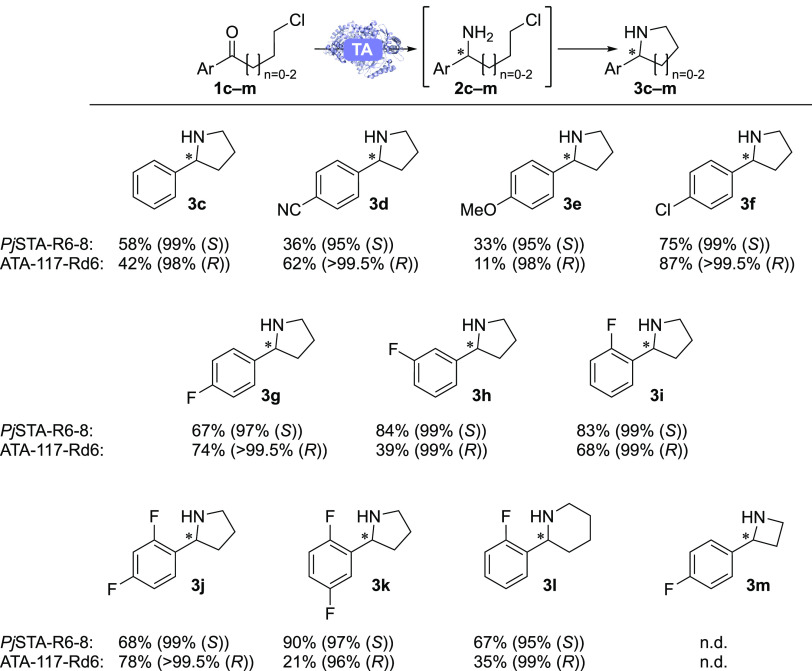
HPLC Yields (Determined Using a Calibration Curve of the Products) and *ee*s (in Parentheses) for the Best-Performing Variants in the Synthesis of **3c-m** Conditions: TA (10 mg/mL), **1c-g** (50 mM), PLP (1 mM), IPA (1 M), DMSO (20% v/v), KP_*i*_-buffer (100 mM), pH 8, 37 °C, 700 rpm, final volume 0.5 mL Reaction time: 48 h. For **3l**: subsequent addition of NaOH (50 μL, 10 M) and further incubation for 1 h. Data are the average of duplicates. N.d.: not detected.

2-Arylpyrrolidines are featured in several bioactive molecules, such as nicotine, larotrectinib,^34^ and MSC2530818 (Figure 4).^35^ Thus, their synthesis from commercially available 4-chlorobutyrophenones (**1c-k**) was investigated (Scheme 2). Given the bulky–bulky nature of these substrates, the focus was shifted toward the engineered TAs 3FCR-4M, *Pj*STA-R6-8, ATA-117-Rd6, and ATA-117-Rd11. Preliminary tests using ATA-117-Rd6 indicated that higher reaction temperatures of 37 °C and longer reaction times of 48 h were required for these more hindered substrates (Figure 5). Thus, screening of the panel of substrates against the panel of bulky–bulky TAs was carried out under these conditions, the comprehensive results of which can be seen in Supporting Figures S5–15. Hydrolysis and cyclopropane formation were observed as the major nonenzymatic side reactions (identified by liquid chromatography–mass spectrometry (LC-MS), see the SI). The desired products were identified by comparison to authentic standards (commercial, except **3d** which was synthesized and characterized by NMR, see the SI) as well as LC-MS (see the SI). The absolute configurations were assigned based on the known selectivities of ATA-117-Rd6^7^ and *Pj*STA^30^ for **1i** and butyrophenone, respectively, as well as consistent elution orders on the chiral GC (see SI), and further confirmed by optical rotation for (*R*)-**3f** (see below). For all substrates, ATA-117-Rd6 was the best (*R*)-selective TA, whereas *Pj*STA-R6-8 was the best (*S*)-selective TA (Scheme 2). ATA-117-Rd6 is less specialized than ATA-117-Rd11 (6 vs 11 rounds of directed evolution toward the synthesis of sitagliptin),^7^ and thus may retain more promiscuity. IPA has been reported to be an unsuitable amine donor for 3FCR-4M.^36^ The additional mutations in ATA-117-Rd11 are concentrated at the upper back of both the large and small binding pockets, resulting in an enlarged cavity that may influence the dynamics of the substrate entering the active site, as well as the tightness of binding (SI Figure S16).

In the synthesis of 2-arylpyrrolidines **3c-k** (Scheme 2), both enzymes showed excellent (≥95% *ee*) and complementary enantioselectivities, while high-performance liquid chromatography (HPLC) yields ranged from low (10%) to excellent (90%). Of particular interest are products **3f** and **3k**, which are motifs featured in the APIs larotrectinib, and MSC2530818 (Figure 4), respectively. In contrast, recent examples of the asymmetric synthesis of 2-aryl-*N*-heterocycles employing a reductive amination strategy using transition-metal catalysts are mostly limited to <90% *ee*, with only a few exceptions.^37−39^ Biocatalytic imine reduction using IREDs generally reaches >99 *ee*;^22^ however, the starting imines for this strategy are typically not commercially available. Fanourakis et al.^40^ reported the synthesis of (*R*)-**3c** in 33% yield and 89% *ee* starting from readily available 4-phenylbutan-1-ol by employing an asymmetric benzylic C-H activation strategy, followed by Mitsunobu cyclization and deprotection.

In the case of ATA-117-Rd6, *para*-substitution with an electron-donating methoxy group resulted in decreased yield, whereas electron-withdrawing chloride, fluoride, and to a lesser extent cyano in that position was beneficial. Fluoride in the *ortho*-position was also beneficial, with di-substitution in both positions showing a synergistic effect. However, a *meta*-fluoro-substituent resulted in decreased yield even in the presence of an additional *ortho*-fluoro group. Docking of the quinonoid intermediates for each substrate (SI Figures S17–26) showed that substitutions on the phenyl ring result in a more twisted configuration around the Cα-N bond. Electron-withdrawing substituents likely increase the yield by increasing the electrophilicity of the carbonyl, increasing its reactivity. The additional increase in yield for the *para*-halogenated compounds may be explained by a π-halogen interaction^41^ with Y61 of the neighboring subunit. A structural explanation for the decreased yields with *meta*-fluoride remains elusive.

*Pj*STA-R6-8 largely showed similar trends to ATA-117-Rd6 with some notable exceptions; a *para*-cyano group was detrimental compared to no substitution, whereas *meta*-fluorination was beneficial, with 2′,5′-difluorination being highly beneficial. This effect may be due to the *meta*-fluoro group pointing into the active site entrance, allowing the *ortho*-fluoride to form a hydrogen bond with the PLP hydroxy group and K288. *para*-Fluorination appears to disrupt the possibility for this hydrogen bond, which may explain why 2′,4′-difluorination exhibited similar HPLC yields to para-fluorination and decreased HPLC yields compared to *ortho*-fluorination (SI Figures S27–36).

Curiously, *Pj*STA-R6-8 only showed very slight preference for the electronically activating *para*-nitrile group over the electronically deactivating *para*-methoxy substituent. This preference may be due to steric interactions of the linear nitrile group with M54 and L417, which are reduced with the flexible bent methoxy group, as well as additional beneficial hydrophobic interactions of the methyl group with M54, L57, and M58 (SI Figures S27–29). For most substrates, *Pj*STA-R6-8 showed higher HPLC yields than ATA-117-Rd6. For the synthesis of 2-arylpiperidine **3l**, additional incubation with base was required to complete cyclization, whereas the 3-chloro-propiophenone **1m** rapidly degraded and no transaminated product was observed (the major degradation product was 3-isopropylamino-propiophenone, as determined by LC-MS, see SI).

**Scheme 3:**
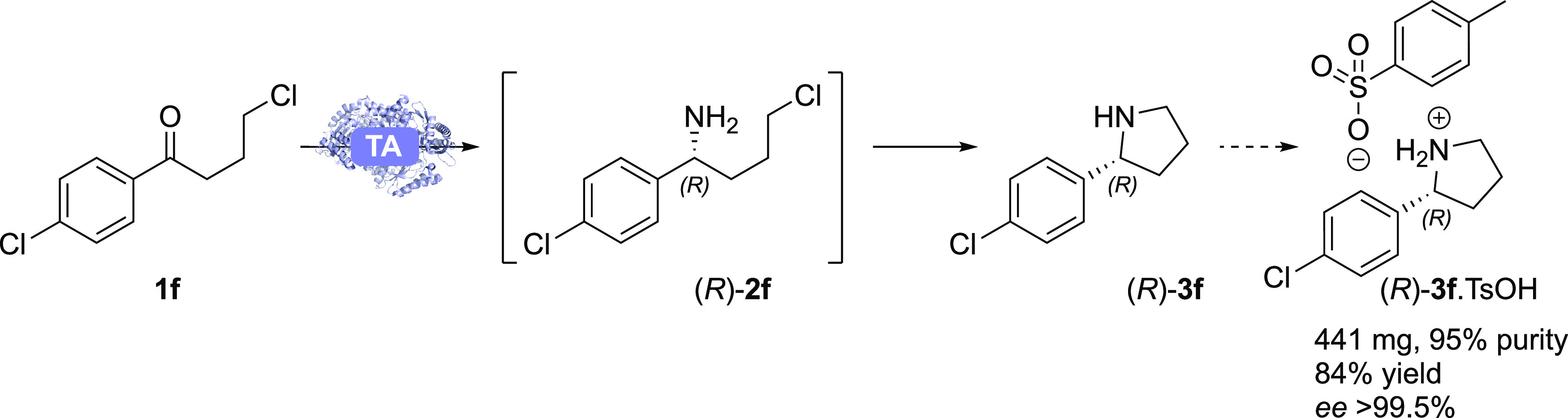
Preparative-Scale Synthesis of (*R*)-**3f** from **1f**, Catalyzed by ATA-117-Rd6 Conditions: ATA-117-Rd6 (250 mg), **1f** (300 mg), PLP (1 mM), IPA (1 M), DMSO (20% v/v), KP_*i*_-buffer (100 mM), pH 8, 40 °C, vigorous stirring (magnetic stirrer), final volume 25 mL. Reaction time: 72 h.

The synthesis of **3f** was then scaled up to 300 mg of starting material using ATA-117-Rd6 (Scheme 3), giving the tosylate salt of (*R*)-**3f** in 84% isolated yield (95% NMR purity, main impurities being water and DMSO), with >99.5% *ee*. As the substrate **1f** is only partially soluble under the reaction conditions, vigorous stirring, higher temperature, and prolonged reaction times were needed to achieve high conversions at this scale due to the lower relative surface area of the aggregated starting material, resulting in reduced mass transfer compared to the analytical scale. The specific rotation (see below) of the free amine of (*R*)-**3f** was consistent with the literature.^42^

## Conclusions

A panel of chiral 2-substituted pyrrolidine and piperidines was synthesized starting from commercially available ω-chloroketones. Both enantiomers could be accessed by choosing the corresponding transaminase, with *ee*s >95% in all cases, and analytical yields ranging from 10% to 90%. Chiral azetidines could not be accessed using this strategy as the starting material was not sufficiently stable under the reaction conditions. Isolation of the product on preparative scale was also possible, the product amine being easily precipitated from MTBE using tosic acid. Thus, the strategy described herein represents a powerful and straightforward way to access chiral pyrrolidine and piperidines on a laboratory scale, with potential applications in drug discovery.^43−45^ The long reaction times, as well as high enzyme loading (83 wt %) require further improvement of the enzymes identified, for example, by directed evolution. Best results for the generation of (*R*)-2-aryl pyrrolidines and piperidines were obtained with ATA-117-Rd6 rather than the usual choice of ATA-117-Rd11 for bulky-bulky ketones,^46^ highlighting that the most engineered enzyme for one specific substrate does not necessarily have the broadest substrate scope.

## Methods

Chemicals were purchased from Merck KGaA (Darmstadt, Germany), TCI Europe N. V. (Zwijndrecht, Belgium), Biosynth s.r.o. (Bratislava, Slovakia), Fluorochem Ltd. (Hadfield, UK), Activate Scientific GmbH (Prien, Germany), abcr GmbH (Karlsruhe, Germany), and Thermo Fisher GmbH (Kandel, Germany). 5-Chloropentan-2-one and 6-chlorohexan-2-one were distilled prior to use, and all other chemicals were used without additional purification. Criterion TGX Stain-Free Precast Gels were used for SDS-PAGE and visualized using a Bio-Rad ChemiDoc Imaging System. Plasmids pET-28a-ATA-117,1 pCH93b-TsRTA,2 pCH93b-AtRTA,3 pMP89a-HEwT,4 and pMP89b-CvSTA5 were received from the Paradisi group, pET-21a-ATA-117-Rd111 was received from the Kroutil group, and pET-20b-PjSTA-R6-86 was received from the Janssen/Fraaije group. pET-28a-ATA-117-Rd61 and pET-28a-3fcr-4M7 were purchased from Synbio Technologies, LLC. (Monmouth Junction, NJ). pMP89a-HEwT W56G8 was prepared using the NEBaseChanger kit (New England Biolabs, Ipswich, MA).

### Transaminase Production

HEwT, HEwT W56G, and *Pj*STA-R6-8 were produced by recombinant expression in *E. coli* BL21(DE3) in ZYP autoinduction medium at 30 °C (24 °C for *Pj*STA-R6-8) overnight.^27^ All other transaminases were produced by recombinant expression in *E. coli* BL21(DE3) in TB-lac medium at 25 °C overnight.^32^ Lyophilized cell-free extracts were then prepared. Further details can be found in the SI.

### Analytical Scale Reactions

Reactions were set up by combining in the following order: potassium phosphate buffer (100 mM, pH 8), PLP (10 mM stock in buffer) isopropylamine (2 M stock (pH adjusted with HCl) in buffer), substrate (250 or 1 M stock in DMSO), and transaminase (50 mg/mL stock in buffer), to give biotransformations (500 μL) containing the desired concentrations of components as referred to in the manuscript (Table S2). Biotransformations were then incubated at 30 or 37 °C, 700 rpm (Eppendorf ThermoMixer C), for 22–48 h, as indicated in the manuscript. For 6-chlorohexan-2-one (**1b**), sodium hydroxide (100 μL, 10 M) was added, followed by further incubation for 3 h. For *o*-fluoro-5-chlorovalerophenone (**1l**), sodium hydroxide (50 μL, 10 M) was added, followed by further incubation for 1 h.

For 5-chloropentan-2-one (**1a**) and 6-chlorohexan-2-one (**1b**), the reactions were allowed to cool and extracted by adding EtOAc (1 mL), followed by NaOH (10 M, 100 μL; 5-chloropentan-2-one (**1a**) only; adding base after adding solvent suppresses cyclopropanation). The extract was further diluted (400 + 600 μL EtOAc), acetylated (20 μL each of Et_3_N + Ac_2_O), and analyzed by both achiral and chiral gas chromatography (GC).

For all other substrates, the reactions were allowed to cool and quenched with acetonitrile (1 vol). An aliquot was further diluted (40 μL + 280 μL H_2_O + TFA (0.2%) + 280 μL MeCN), centrifuged, and analyzed by high-performance liquid chromatography (HPLC). (Alternatively, 100 μL sample + 450 μL H_2_O + HCl (0.2%) + 280 μL MeCN for LC-MS.) The remainder was alkalized with NaOH (10 M, 80 μL; except *o*-fluoro-5-chlorovalerophenone (**1l**)) and extracted into EtOAc (1 mL), acetylated (20 μL each of Et_3_N + Ac_2_O), and analyzed by chiral GC.

### Preparative Scale Reaction—Synthesis of (*R*)-3f·TsOH

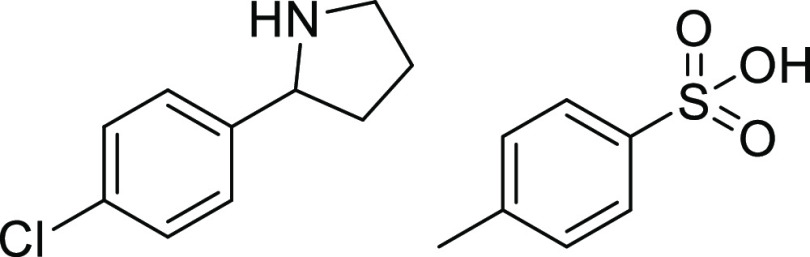



In a round-bottom flask equipped with a magnetic stirrer bar, **1f** (300 mg, 1.38 mmol) was dissolved in DMSO (5 mL) after which IPA (12.5 mL of 2 M stock pH adjusted (HCl) stock in KP_*i*_ -buffer (100 mM, pH 8)) was added. To the cloudy suspension was then added ATA-117-Rd6 (250 mg), which had been rehydrated in buffer (7.5 mL of PLP (3.33 mM) in KP_*i*_ -buffer (100 mM, pH 8)). The reaction was then protected from light (aluminum foil) and stirred vigorously (just below vortex formation) at 40 °C (oil bath) for 72 h. The reaction was allowed to cool, acidified to pH < 3 (320 μL of conc. HCl), and extracted with EtOAc (2 × 10 mL), breaking the emulsion with centrifugation (5000*g* for 1 min). The aqueous phase was then basified to pH >11 (3 mL of 10 M NaOH) and extracted with EtOAc (3 × 10 mL), breaking the emulsion with centrifugation (5000*g* for 1 min). The organic extracts were combined, dried with MgSO_4_, and concentrated *in vacuo*. The oily residue was then re-dissolved in methyl *tert*-butyl ether (MTBE; 3 mL), and tosic acid monohydrate (270 mg, 1.42 mmol), pre-dissolved in MTBE (5 mL), was slowly added, causing immediate precipitation. After incubation at −20 °C for 30 min, the solid was separated by vacuum filtration, washed with ice-cold MTBE (2 × 2 mL), and dried overnight at 0.34 mbar, giving fine free-flowing white crystals of (*R*)-**3f**.TsOH (441 mg, 95% purity (^1^H-NMR), 80% purity-adjusted yield). The main impurities detected were DMSO and water (overlapping with the 2-hydrogen of the pyrrolidine ring). [α]_*D*_^21^ = +51° (free amine, *c* 0.883, CH_2_Cl_2_); ^1^H-NMR (400 MHz, CDCl_3_, referenced relative to TMS) δ 1.92–2.18 (3 H, m), 2.19–2.31 (1 H, m), 2.38 (3 H, s), 3.24–3.51 (2 H, m), 4.35–4.52 (m overlapping with water), 7.10–7.17 (4 H, m), 7.26–7.32 (2 H, m), 7.48–7.53 (2 H, m), 8.59 (1 H, br s), 9.35 (1 H, br s); ^13^C-NMR (101 MHz, CDCl_3_, absolute referencing relative to ^1^H-NMR) δ 21.4 (CH_3_), 23.5 (CH_2_), 31.3 (CH_2_), 45.5 (CH_2_), 62.7 (CH), 125.8 (CH), 129.0 (CH), 129.1 (CH), 129.4 (CH), 132.6 (C), 135.1 (C), 140.8 (C), 140.9 (C); Retention times on GC and HPLC match those of commercial **3f**.

### Analytical methods

Detailed analytical methods are found in the SI.

## Supporting Information Available

Production of enzymes, protocols, analytical measurements, dockings, GC and HPLC chromatograms, and NMR spectra (PDF)
