# Supplementary material for: Enantio-Complementary Synthesis of 2-Substituted Pyrrolidines and Piperidines via Transaminase-Triggered Cyclizations
Source: JACS Au. 2023 May 12;3(6):1642–9. doi: 10.1021/jacsau.3c00103 (PMC10301811; doi:10.1021/jacsau.3c00103)
Supplement: Supplementary file 1 — au3c00103_si_001.pdf [file au3c00103_si_001.pdf]

Electronic Supporting Information

Enantio-complementary synthesis of 2-substituted pyrrolidines and piperidines via transaminase-triggered cyclizations

Christian M. Heckmann,[a]* Caroline E. Paul[a]*

[a] *Biocatalysis section, Department of Biotechnology, Delft University of Technology, Van der Maasweg 9, 2629 HZ Delft, The Netherlands*
* Corresponding authors: c.m.heckmann@tudelft.nl, c.e.paul@tudelft.nl

Contents

Materials and Methods 3
  Transaminase production 3
    Production of HEwT, HEwT W56G, and PjSTA-R6-8 3
    Production of all other transaminases 3
    Preparation of lyophilized cell-free extracts (CFEs) 3
    Specific activity measurements 3
  Analytical scale reactions 4
  Synthesis of **3d**·2TsOH 4
  Analytical methods 5
    HPLC 5
    GC-FID 6
    LC-MS 7
  Docking studies 7
Supplementary Figures 8
Chromatograms 28
  GC-FID chromatogram of biotransformations of **1a** 28
  GC-FID chromatogram of biotransformations of **1b** 28
  HPLC chromatogram of biotransformations of **1c** 28
  HPLC chromatogram of biotransformations of **1d** 29
  HPLC chromatogram of biotransformations of **1e** 29
  HPLC chromatogram of biotransformations of **1f** 29
  HPLC chromatogram of biotransformations of **1g** 30
  HPLC chromatogram of biotransformations of **1h** 30
  HPLC chromatogram of biotransformations of **1i** 30
  HPLC chromatogram of biotransformations of **1j** 31
  HPLC chromatogram of biotransformations of **1k** 31
  HPLC chromatogram of biotransformations of **1l** 31
  HPLC chromatogram of biotransformations of **1m** 32
  Extracted ion count (**3d**) LC-MS trace of biotransformations of **1d** 36
  Extracted ion count (**3e**) LC-MS trace of biotransformations of **1e** 37
  Extracted ion count (**3f**) LC-MS trace of biotransformations of **1f** 37
  Extracted ion count (**3g**) LC-MS trace of biotransformations of **1g** 38
  Extracted ion count (**3h**) LC-MS trace of biotransformations of **1h** 38
  Extracted ion count (**3i**) LC-MS trace of biotransformations of **1i** 39
  Extracted ion count (**3j**) LC-MS trace of biotransformations of **1j** 39
  Extracted ion count (**3k**) LC-MS trace of biotransformations of **1k** 40
  Extracted ion count (**3l**) LC-MS trace of biotransformations of **1l** 40
  Extracted ion count (**3m**) LC-MS trace of biotransformations of **1m** 41

Extracted ion count (ω-isopropylamine-ketone) LC-MS trace of biotransformations of **1m** 41
Chiral GC-FID chromatogram of biotransformations of **1a** 42
Chiral GC-FID chromatogram of biotransformations of **1b** 42
Chiral GC-FID chromatogram of biotransformations of **1c** 42
Chiral GC-FID chromatogram of biotransformations of **1d** 43
Chiral GC-FID chromatogram of biotransformations of **1e** 43
Chiral GC-FID chromatogram of biotransformations of **1f** 43
Chiral GC-FID chromatogram of biotransformations of **1g** 44
Chiral GC-FID chromatogram of biotransformations of **1h** 44
Chiral GC-FID chromatogram of biotransformations of **1i** 44
Chiral GC-FID chromatogram of biotransformations of **1j** 45
Chiral GC-FID chromatogram of biotransformations of **1k** 45
Chiral GC-FID chromatogram of biotransformations of **1l** 45
Chiral GC-FID chromatogram of isolated (R)-**3f** from preparative biotransformation 46
NMR spectra 47
References 53

Materials and Methods

Transaminase production

Production of HEwT, HEwT W56G, and PjSTA-R6-8

E. coli BL21(DE3) chemically competent cells were transformed with the vector baring the gene and grown on selective LB–agar plates (ampicillin 100 µg/mL) at 37 °C overnight. ZYP–AI medium[9] (500 mL in either a 5 L non-baffled or 2 L baffled flask; ampicillin 100 µg/mL) was inoculated with a single colony (or 5% of overnight LB preculture) of transformed *E. coli* and incubated for 2-6 h at 37 °C, 170 rpm (1 inch throw) followed by 19-22 h at 30 °C (24 °C for *Pj*STA-R6-8), 170 rpm (1 inch throw). Cells were harvested (4500 × *g*, 20 min, 4 °C) and stored at –20 °C.

Production of all other transaminases

The plasmid containing the gene was transformed into *E. coli* BL21(DE3) chemically competent cells. 500 mL of TB-lac (ampicillin 100 µg/mL or kanamycin 50 µg/mL; lactose monohydrate 5 g/L; either in a 5 L non-baffled or 2 L baffled flask) were inoculated with a single colony (or 2% of overnight LB preculture) and incubated at 37 °C, with shaking (170 rpm, 1 inch throw) for 4-6 h followed by 25 °C with shaking (170 rpm, 1 inch throw) for 19-21 h. Cells were harvested by centrifugation (4500 × *g*, 20 min, 4 °C) and stored at -20 °C.

Preparation of lyophilized cell-free extracts (CFEs)

Pellets were resuspended in buffer (potassium phosphate (50 mM), PLP (0.1 mM), pH 8.0; approx. 3:1 v:w) with the aid of sonication, and lysed using a Constant Systems Continuous Flow Cell Disrupter CF1 (two cycles, 21 kpsi) and clarified by centrifugation (48,000 × *g*, 30 min, 4 °C). The CFE was filtered (0.2 µm), frozen (liquid nitrogen), and lyophilized to obtain a beige powder, which was stored at -20 °C.

Specific activity measurements

Activity assays were based on the method by Schätzle et al.,[10] using (*S*)-methylbenzylamine for STAs[4] and (*R*)-methylbenzylamine for RTAs.[2] Assays contained methylbenzylamine (2.5 mM), pyruvate (2.5 mM), PLP (0.01 mM), DMSO (0.25%), and potassium phosphate buffer (50 mM); pH 8. Activities were measured in UV-free 96-well plates using a BioTek Synergy plate reader at 30 °C (path length correction calculated according to $(A_{977}-A_{900})/0.18$), $\varepsilon_{acetophenone} = 12.6\ mM^{-1}\ cm^{-1}$). One unit of activity was defined as the production of 1 µmol of acetophenone per minute.

Table S1: Specific activities of the TA CFEs ± 1 standard error, measured in triplicate.

TA	Specific activity (U/mg)
HEwT	0.28 ± 0.02
HEwT W56G	0.040 ± 0.001
*Cv*STA	0.157 ± 0.009
3FCR_4M	0.030 ± 0.001
*Pj*STA	0.0010 ± 0.0002
*At*RTA	0.142 ± 0.004
*Ts*RTA	0.254 ± 0.003
ATA-117	0.142 ± 0.005
ATA-117 Rd6	0.0009 ± 0.0001
ATA-117 Rd11	0.0017 ± 0.0003

Analytical scale reactions

Table S2: Volumes of stock solutions added to reach reaction conditions as indicated in manuscript.

	Volume added to reaction	
Stock solution (concentration)	Reactions with **1a** and **1b**	All other substrates
potassium phosphate buffer (100 mM, pH 8)	200 µL or 75 µL	0 µL
PLP (10 mM) in buffer	50 µL	50 µL
IPA (2 M) in buffer	125 µL or 250 µL	250 µL
Substrate (1 M) in DMSO	25 µL	-
Substrate (250 mM) in DMSO	-	100 µL
TA (50 mg/mL) in buffer	100 µL	100 µL

Synthesis of **3d**·2TsOH

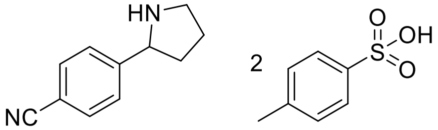

Sodium cyanoborohydride releases highly toxic hydrogen cyanide gas under acidic conditions. All waste streams were treated with bleach before disposal and all glassware was rinsed with bleach after use.

To methanol (1 mL) were added under nitrogen atmosphere (balloon) *p*-cyanobutyrophenone **1d** (99.7 mg, 480 µmol) and ammonium acetate (196 mg, 2.52 mmol) and the resulting suspension was stirred at ambient temperature for 17 h. Sodium cyanoborohydride (49 mg, 0.78 mmol) was dissolved in methanol (200 µL) and added to the reaction mixture via syringe and stirred for 24 h, during which the suspension fully dissolved. A second portion of sodium cyanoborohydride (50 mg, 0.80 mmol) was added as before, and the suspension stirred for an additional 24 h. The reaction was monitored by TLC and had not gone to completion. Water (3 mL) and sodium hydroxide (10 N, 0.4 mL) was added, and the mixture was extracted with ethyl acetate (3×2 mL). The organic extracts were combined, dried ($MgSO_4$), and concentrated *in vacuo*. The resulting sticky clear residue was re-dissolved in MTBE (4 mL) and methanol (2.5 mL), and tosic acid monohydrate (105 mg, 0.552 mmol), pre-dissolved in MTBE (1 mL; dried with $MgSO_4$), was slowly added, causing immediate precipitation. After incubation at -20 °C for 3 days, the solid was separated by vacuum filtration, washed with ice-cold MTBE (2×2mL), and dried for 6 h at 0.47 mbar, giving fine free-flowing white crystals of **3d**·2TsOH (23.3 mg, 9% yield).

^{1}H-NMR (400 MHz, MeCN-d_3 + DMSO-d_6 (5:1), referenced relative to the residual proton peak of MeCN-d_3 (1.94 ppm)) δ 1.98–2.18 (3 H, m), 2.31 (6 H, s), 2.33–2.44 (1 H, m), 3.28–3.46 (2 H, m), 4.54–4.64 (1 H, m), 7.11–7.16 (4 H, m), 7.55 (4 H, dt, *J* 8.0, 2.0 Hz), 7.62 (2 H, dtd, *J* 8.5, 2.0, 0.5 Hz), 7.75 (2 H, dt, *J* 8.5, 2.0 Hz), 8.98 (1 H, br s),

9.61 (1 H, br s); ^{13}C-NMR (101 MHz MeCN-d_3 + DMSO-d_6 (5:1), absolute referencing relative to ^{1}H-NMR) δ 19.6 (CH_3), 22.5 (CH_2), 29.8 (CH_2), 44.6 (CH_2), 61.4 (CH), 111.6 (C), 117.5 (C), 124.9 (CH), 127.6 (CH), 128.0 (CH), 131.9 (CH), 138.1 (C), 139.7 (C), 144.1 (C).

Analytical methods

HPLC

Conversions of reactions with 4-chlorobutyrophenones, 5-chlorovalerophenones, and 3-chloropropiophenones were determined by RP-HPLC-DAD on a Shimadzu Prominence HPLC equipped with a SIL-20A auto-sampler and SPD-M20A DAD detector, using a Restek Raptor ARC-18 column (150 mm × 4.6 mm × 2.7 µm). Oven temperature 30 °C, injection volume 2 µL, flow rate 1 mL/min.

Gradient:

**Time (min)**	**H_2O, TFA (0.1 %)**	**MeCN, TFA (0.1 %)**
0	95	5
2	95	5
12	0	100
14	0	100
14.1	95	5
21	95	5

GC-FID

Conversions of reactions with 5-chloropentan-2-one or 6-chlorohexan-2-one were determined by GC-FID on a GC-2010 Plus (Shimadzu, Japan) equipped with an AOC-20i auto injector and a flame ionization detector (FID), using an Agilent CP-Sil 8 CB column (25 m × 0.25 mm × 1.2 µm). 1 µL of sample was injected with a split ratio of 25:1 and injector temperature of 340 °C. The FID was maintained at 360 °C. Nitrogen was used as the carrier gas, with an initial linear velocity of 30 cm/s.

Temperature program:

**Ramp (°C/min)**	**Temperature (°C)**	**Hold (min)**
	50	1
20	345	1

Enantiomeric excess was determined by GC-FID on a GC-2010 Plus (Shimadzu, Japan) equipped with an AOC-20i auto injector and a flame ionization detector (FID), using either a Hydrodex β-6TBDM column (50 m × 0.25 mm), or Agilent J&W CP-Chirasil-Dex CB column (25m × 0.32 mm × 0.25 µm). Samples were acetylated prior to analysis (20 µL each of Et_3N+Ac_2O in 1 mL). 1 µL of sample was injected with a split ratio of 50:1 or 25:1 and injector temperature of 250 °C. The FID was maintained at 275 °C. Helium was used as the carrier gas, with an initial linear velocity of 38 cm/s (Hydrodex β-6TBDM) or 30 cm/s (CP-Chirasil-Dex).

Temperature programs:

2-methylpyrrolidine: Hydrodex β-6TBDM

**Ramp (°C/min)**	**Temperature (°C)**	**Hold (min)**
	50	1
7.5	180	2
7.5	245	1

2-methylpiperidine: CP-Chirasil-Dex

**Ramp (°C/min)**	**Temperature (°C)**	**Hold (min)**
	50	1
7.5	200	1

2-(*p*-cyanophenyl)pyrrolidine: Hydrodex β-6TBDM

**Ramp (°C/min)**	**Temperature (°C)**	**Hold (min)**
	150	2
3	245	10

2-(2′,5′-difluorophenyl)pyrrolidine: Hydrodex β-6TBDM

**Ramp (°C/min)**	**Temperature (°C)**	**Hold (min)**
	130	2
2	155	35
20	245	2

2-(*o*-fluorophenyl)piperidine: Hydrodex β-6TBDM

**Ramp (°C/min)**	**Temperature (°C)**	**Hold (min)**
	140	2
2	170	30
20	245	2

All other 2-arylpyrrolidines: Hydrodex β-6TBDM

**Ramp (°C/min)**	**Temperature (°C)**	**Hold (min)**
	100	2
5	245	5

LC-MS

High-resolution mass analysis was performed using a Q Exactive™ Focus Hybrid Quadrupole-Orbitrap™ Mass Spectrometer (Thermo Scientific, Germany) connected to an Acquity M Class liquid chromatography system (Waters, UK). The chromatographic separation was performed using a 100 × 1.0 mm BEH C18 column (1.7 µm, Acquity, UPLC column) at a constant flow rate of 25 µL/min. Solvent A consisted of H_2O plus 0.1% formic acid and a solvent B was acetonitrile + 0.1% formic acid. After 2.5 minutes constant at 90% solvent A (10% solvent B), a linear gradient from 10% B to 80% B was applied over 22.5 min. High-resolution mass analysis was performed in positive ionisation mode over a mass range of 100–500 m/z. The resolution was set to 70K, the AGC target to 1.0e6 and the maximum injection time of 75 ms. The mass spectrometric analysis included additional all ion fragmentation scans which were acquired between 75-250 m/z using a NCE of 24 and an AGC target of 3e6. Samples were diluted 1:25 with solvents A and B (mixed in starting condition ratio) before injection. An aliquot of 5 µL was injected to LC-MS analysis system. The mass spectrometric raw data were analysed manually using the Xcalibur software tool (Thermo Scientific, Germany), and after conversion to mzXML by msconvert (ProteoWizard),[11] using the GNPS dashboard https://gnps-lcms.ucsd.edu/.[12]

Docking studies

Homology models of HEwT W56G and ATA-117-Rd6 were generated using SWISS-MODEL[13] based on crystal structures of HEwT wt (PDB 6gwi) and ATA-117-Rd11 (PDB 5fr9), respectively. Structures for *Pj*STA-R6-8 (PDB 7b4j), HEwT wt (PDB 6gwi), and ATA-117-Rd11 (PDB 5fr9) were retrieved from the protein databank. Structures were loaded into YASARA 20.12.24 and the force-field AMBER03 (cut-off 10.5 Å, PME for long-range electrostatics) was applied. Structures were cleaned (CleanObj All) and charged (pH 8, ChargeObj All). A simulation cell around the cofactor was generated, and the co-factor deleted. Quinonoid intermediates were generated using IQmol. Docking was carried out using the macro dock_run.mcr. Best poses were selected based on the resemblance of the PLP binding in comparison with PLP in the crystal structures or homology model. Figures were generated using Open Source PyMOL 2.5.0.

Supplementary Figures

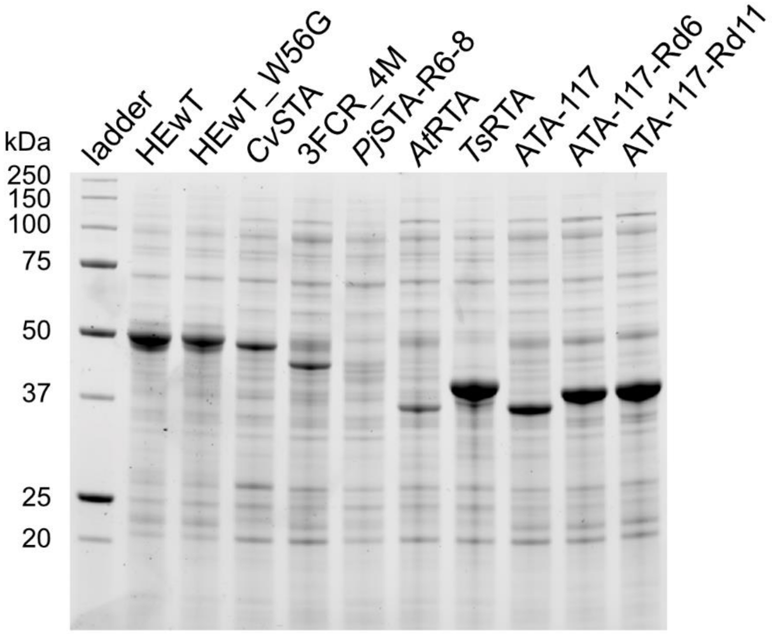

Figure S1: SDS-PAGE gels of the TAs used in this study, 15 µg of lyophilized CFE per well. Expected molecular weights: HEwT (54.2 kDa), HEwT_W56G (54.1 kDa), *Cv*STA (55.2 kDa), 3FRC_4M (50.9 kDa), *Pj*STA-R6-8 (50.4 kDa), *At*RTA (39.8 kDa), *Ts*RTA (40.5 kDa), ATA-117 (38.7 kDa), ATA-117-Rd6 (37.7 kDa), ATA-117-Rd11 (37.7 kDa). Expression of *Pj*STA-R6-8 was predominantly insoluble (not shown).

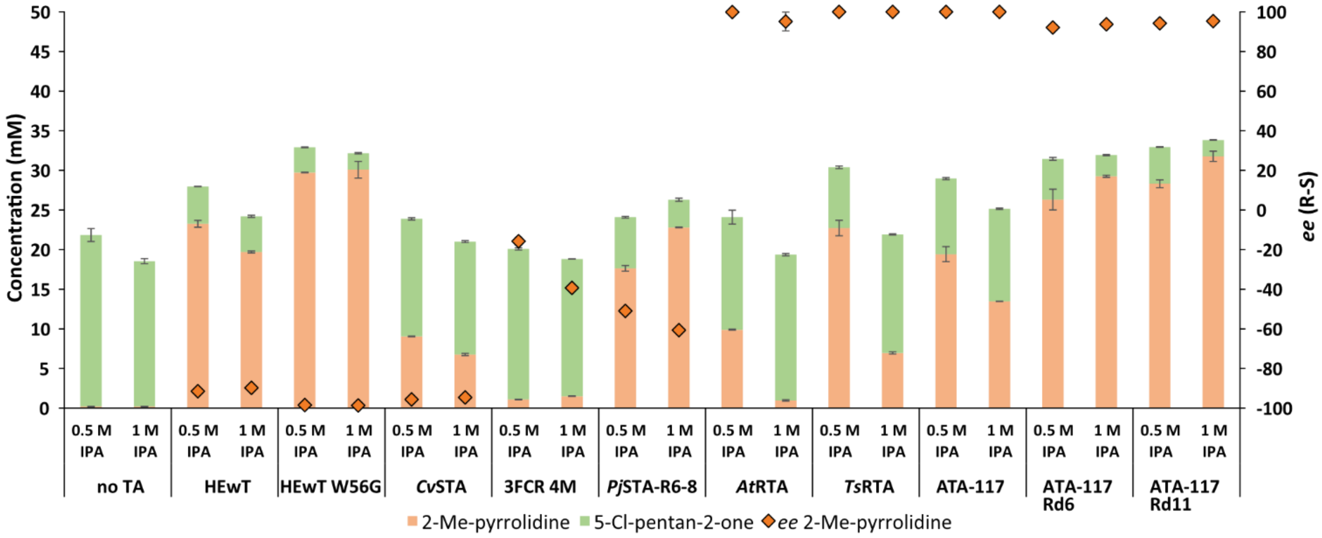

Figure S2: Screening of the transaminase enzyme panel against 5-chloropentan-2-one **1a**. Conditions: TA (10 mg/mL), 5-chloropentan-2-one (50 mM), PLP (1 mM), DMSO (5% v/v), KP_i-buffer (100 mM), pH 8, 30 °C, 700 rpm, 22h (*Pj*STA-R6-8: 24 h). Reaction volume 0.5 mL. Concentrations determined by GC using a calibration curve. Data are the average of duplicates; error bars are standard errors.

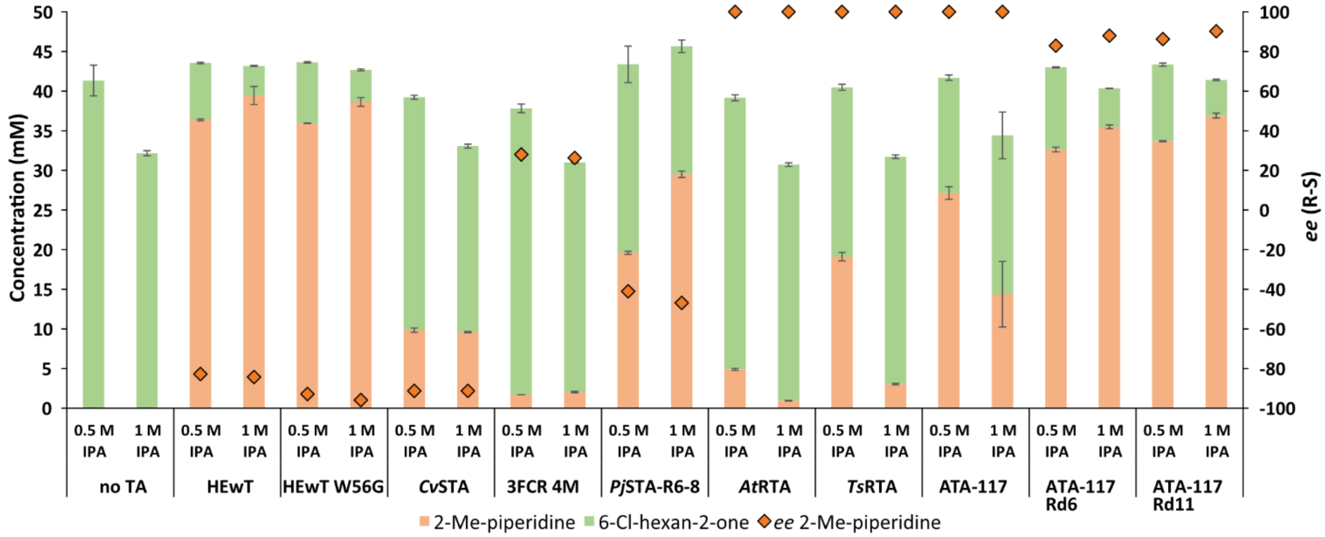

Figure S3: Screening of the transaminase enzyme panel against 6-chlorohexan-2-one **1b**. Conditions: TA (10 mg/mL), 5-chloropentan-2-one (50 mM), PLP (1 mM), DMSO (5% v/v), KP$_i$-buffer (100 mM), pH 8, 30 °C, 700 rpm, 24 h. Reaction volume 0.5 mL. Addition of NaOH (100 µL, 10 M) followed by further incubation for 3 h. Concentrations determined by GC using a calibration curve. Data are the average of duplicates; error bars are standard errors.

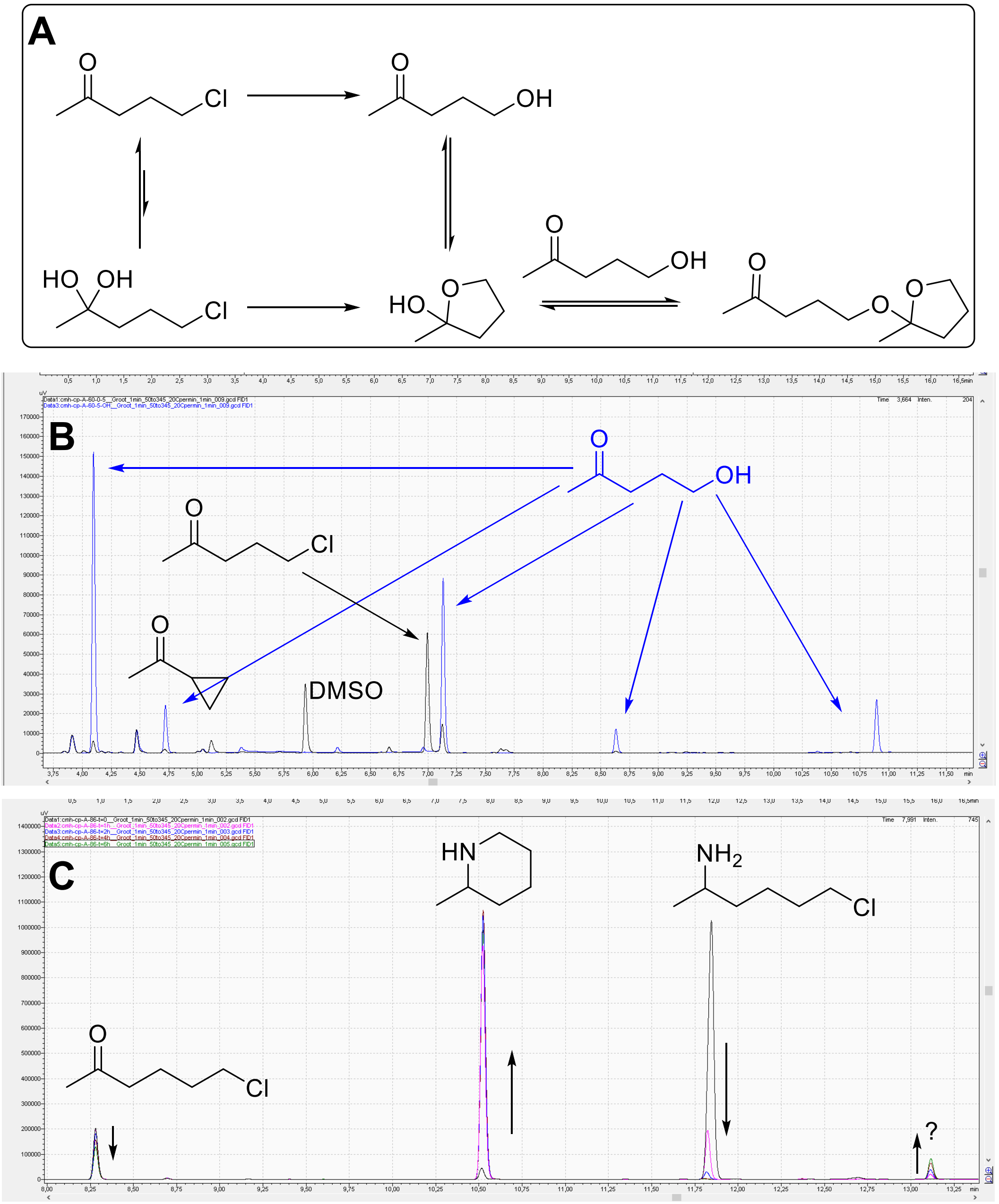

Figure S4: **A**: Hydrolysis of 5-chloropentan-2-one **1a**, as well as its equilibria with the hemiacetal and dimer form, and a putative route via the hydrate which may explain why hydrolysis is only observed with **1a** but not **1b** (the hydrate is expected only in trace amounts[14] and was never detected). **B**: GC chromatogram overlay of commercial standard of 5-hydroxypentan-2-one (blue) with 5-chloropentan-2-one **1a** after incubation under reaction conditions (black) (analyzed without acetylation). In addition to the three expected peaks (hydroxy ketone, hydrate, and dimer), the commercial standard also showed two extra peaks, one of which matches methyl cyclopropyl ketone. Thus, quantification of the hydrolysis is challenging but its presence was clearly detected and likely accounts for most of the loss of material. **C**: GC chromatogram overlay of the time-course of the incubation with excess sodium hydroxide for 0 h (black), 1 h (pink), 2 h (blue), 4 h (brown), and 6 h (green) following a 24 h biotransformation with HEwT. The unidentified side-product forming from the unreacted starting material is likely the hydrolysis, E2-elimation, or aminolysis product.

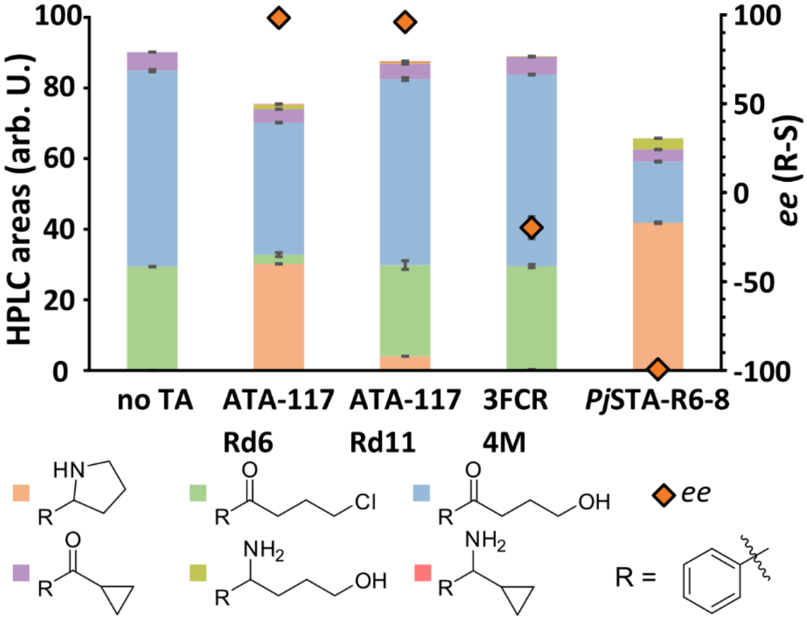

Figure S5: HPLC areas and *ee*s for the synthesis of **3c**. Conditions: TA (10 mg/mL), **1c** (50 mM), PLP (1 mM), IPA (1 M), DMSO (20% v/v), KPi-buffer (100 mM), pH 8, 37 °C, 700 rpm, final volume 0.5 mL. Reaction time: 48 h. Data are the average of duplicates; error bars represent standard errors.

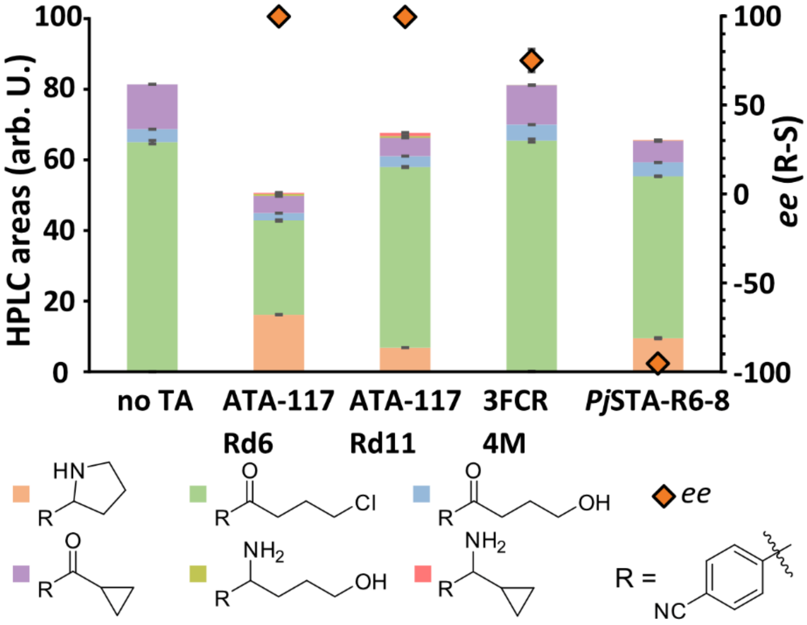

Figure S6: HPLC areas and *ee*s for the synthesis of **3d**. Conditions: TA (10 mg/mL), **1d** (50 mM), PLP (1 mM), IPA (1 M), DMSO (20% v/v), KPi-buffer (100 mM), pH 8, 37 °C, 700 rpm, final volume 0.5 mL. Reaction time: 48 h. Data are the average of duplicates; error bars represent standard errors.

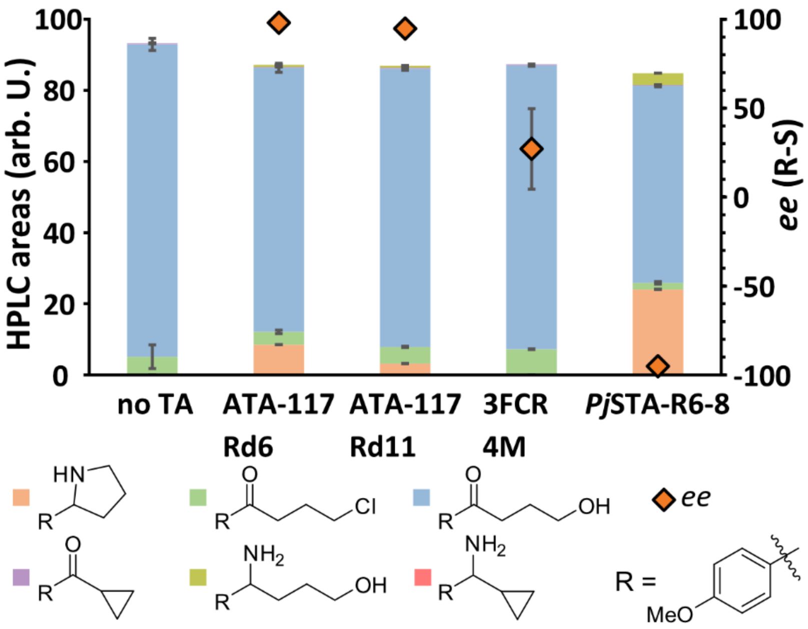

Figure S7: HPLC areas and *ee*s for the synthesis of **3e**. Conditions: TA (10 mg/mL), **1e** (50 mM), PLP (1 mM), IPA (1 M), DMSO (20% v/v), KPi-buffer (100 mM), pH 8, 37 °C, 700 rpm, final volume 0.5 mL. Reaction time: 48 h. Data are the average of duplicates; error bars represent standard errors.

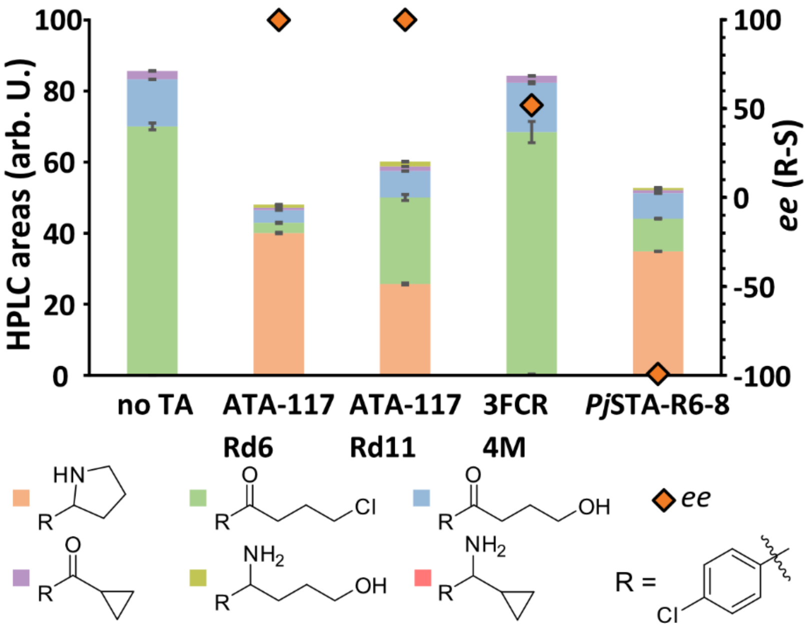

Figure S8: HPLC areas and *ee*s for the synthesis of **3f**. Conditions: TA (10 mg/mL), **1f** (50 mM), PLP (1 mM), IPA (1 M), DMSO (20% v/v), KPi-buffer (100 mM), pH 8, 37 °C, 700 rpm, final volume 0.5 mL. Reaction time: 48 h. Data are the average of duplicates; error bars represent standard errors.

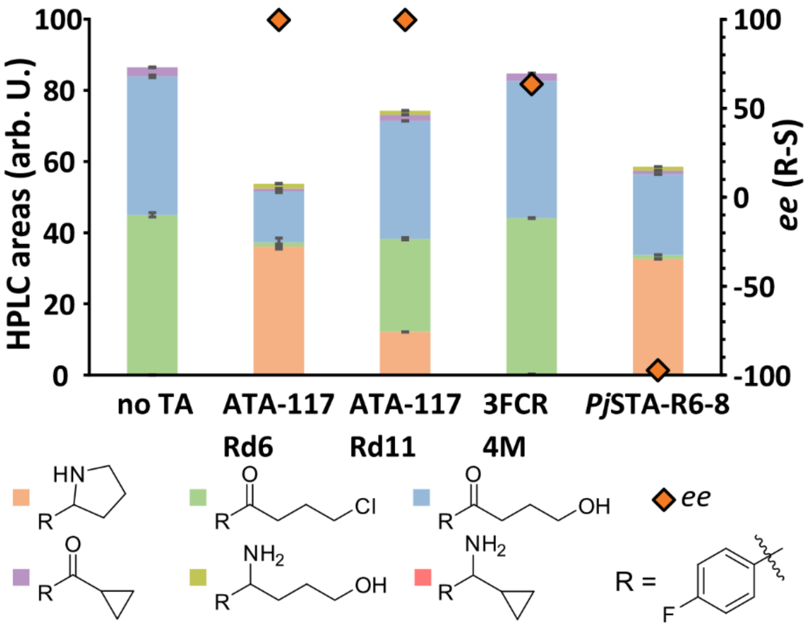

Figure S9: HPLC areas and *ee*s for the synthesis of **3g**. Conditions: TA (10 mg/mL), **1g** (50 mM), PLP (1 mM), IPA (1 M), DMSO (20% v/v), KPi-buffer (100 mM), pH 8, 37 °C, 700 rpm, final volume 0.5 mL. Reaction time: 48 h. Data are the average of duplicates; error bars represent standard errors.

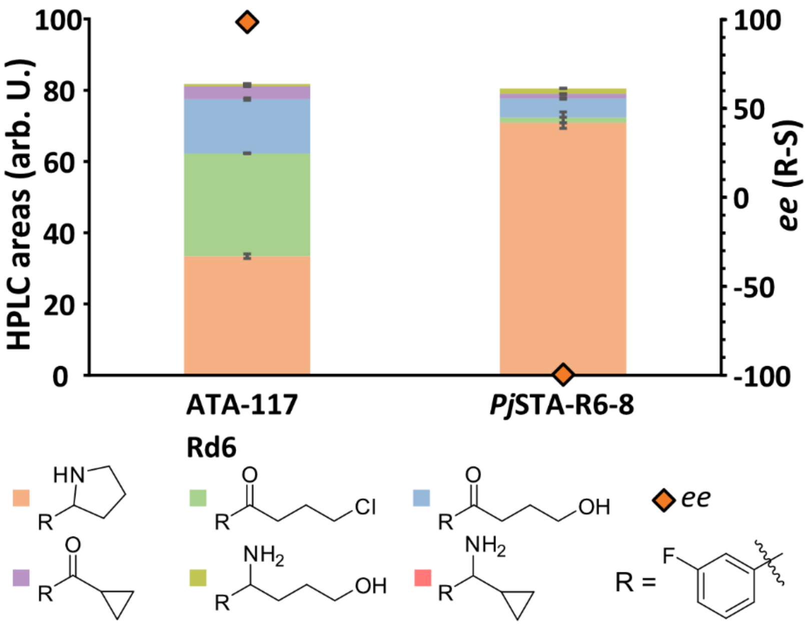

Figure S10: HPLC areas and *ee*s for the synthesis of **3h**. Conditions: TA (10 mg/mL), **1h** (50 mM), PLP (1 mM), IPA (1 M), DMSO (20% v/v), KPi-buffer (100 mM), pH 8, 37 °C, 700 rpm, final volume 0.5 mL. Reaction time: 48 h. Data are the average of duplicates; error bars represent standard errors.

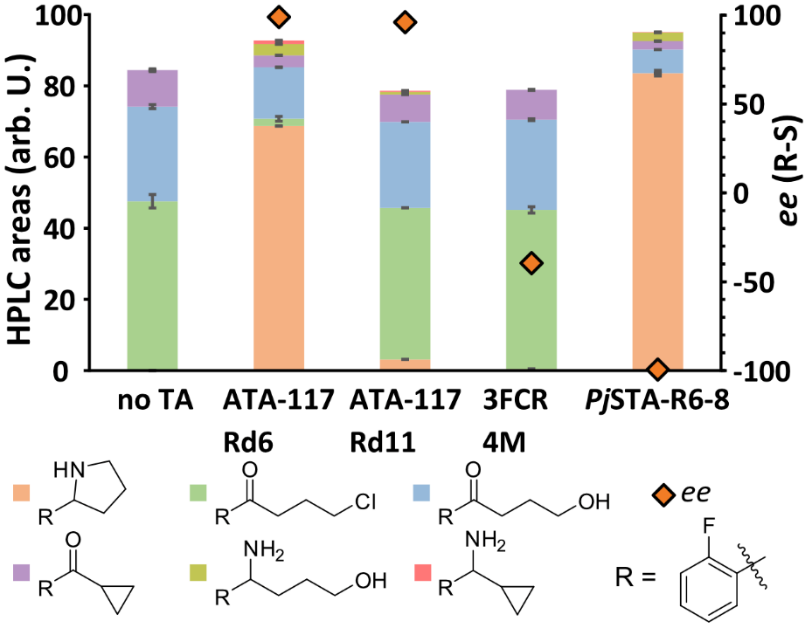

Figure S11: HPLC areas and *ee*s for the synthesis of **3i**. Conditions: TA (10 mg/mL), **1i** (50 mM), PLP (1 mM), IPA (1 M), DMSO (20% v/v), KPi-buffer (100 mM), pH 8, 37 °C, 700 rpm, final volume 0.5 mL. Reaction time: 48 h. Data are the average of duplicates; error bars represent standard errors.

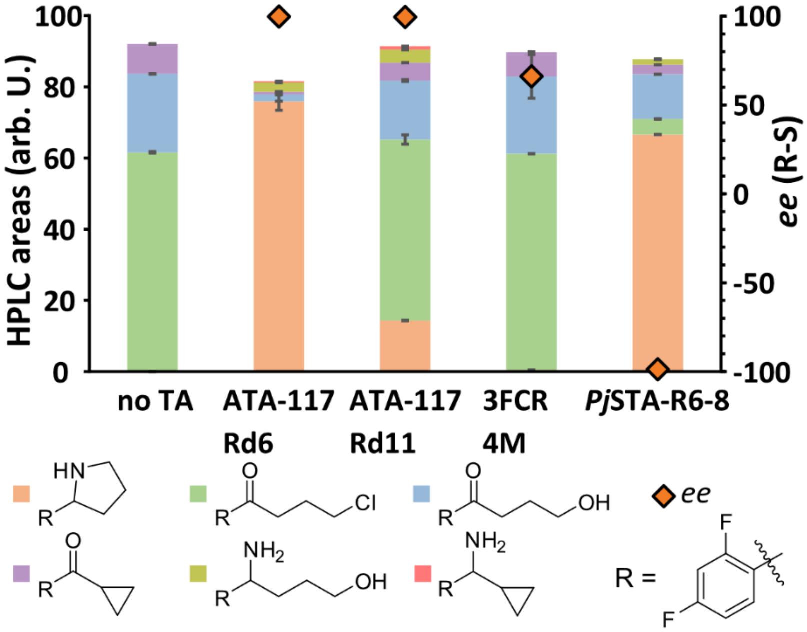

Figure S12: HPLC areas and *ee*s for the synthesis of **3j**. Conditions: TA (10 mg/mL), **1j** (50 mM), PLP (1 mM), IPA (1 M), DMSO (20% v/v), KPi-buffer (100 mM), pH 8, 37 °C, 700 rpm, final volume 0.5 mL. Reaction time: 48 h. Data are the average of duplicates; error bars represent standard errors.

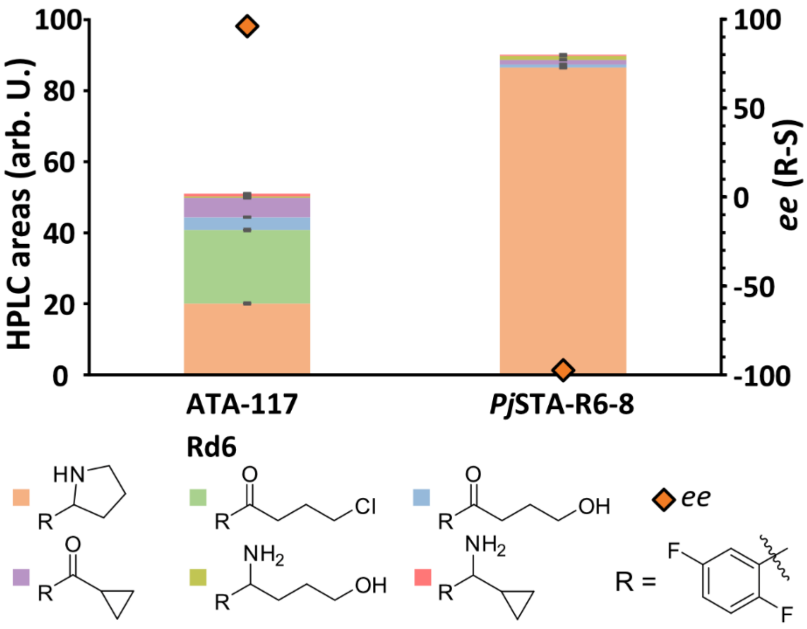

Figure S13: HPLC areas and *ee*s for the synthesis of **3k**. Conditions: TA (10 mg/mL), **1k** (50 mM), PLP (1 mM), IPA (1 M), DMSO (20% v/v), KPi-buffer (100 mM), pH 8, 37 °C, 700 rpm, final volume 0.5 mL. Reaction time: 48 h. Data are the average of duplicates; error bars represent standard errors.

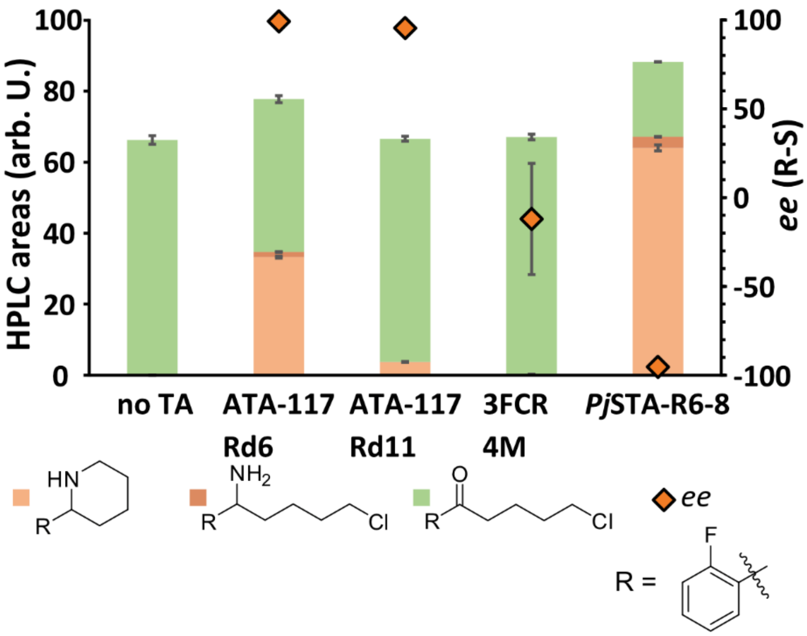

Figure S14: HPLC areas and *ee*s for the synthesis of **3l**. Conditions: TA (10 mg/mL), **1l** (50 mM), PLP (1 mM), IPA (1 M), DMSO (20% v/v), KPi-buffer (100 mM), pH 8, 37 °C, 700 rpm, final volume 0.5 mL. Reaction time: 48 h, followed by addition of NaOH (50 µL, 10 M) and further incubation for 1h. Data are the average of duplicates; error bars represent standard errors.

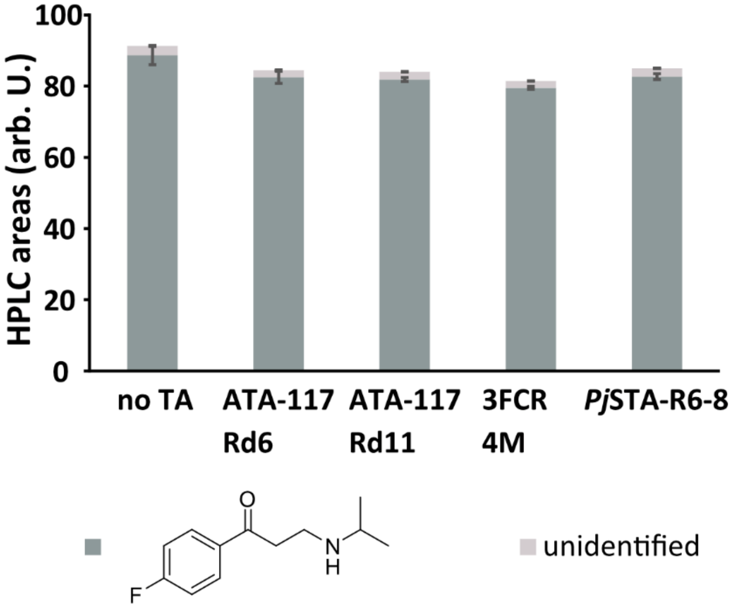

Figure S15: HPLC areas with **1m** as substrate. Conditions: TA (10 mg/mL), **1m** (50 mM), PLP (1 mM), IPA (1 M), DMSO (20% v/v), KPi-buffer (100 mM), pH 8, 37 °C, 700 rpm, final volume 0.5 mL. Reaction time: 48 h. Data are the average of duplicates; error bars represent standard errors.

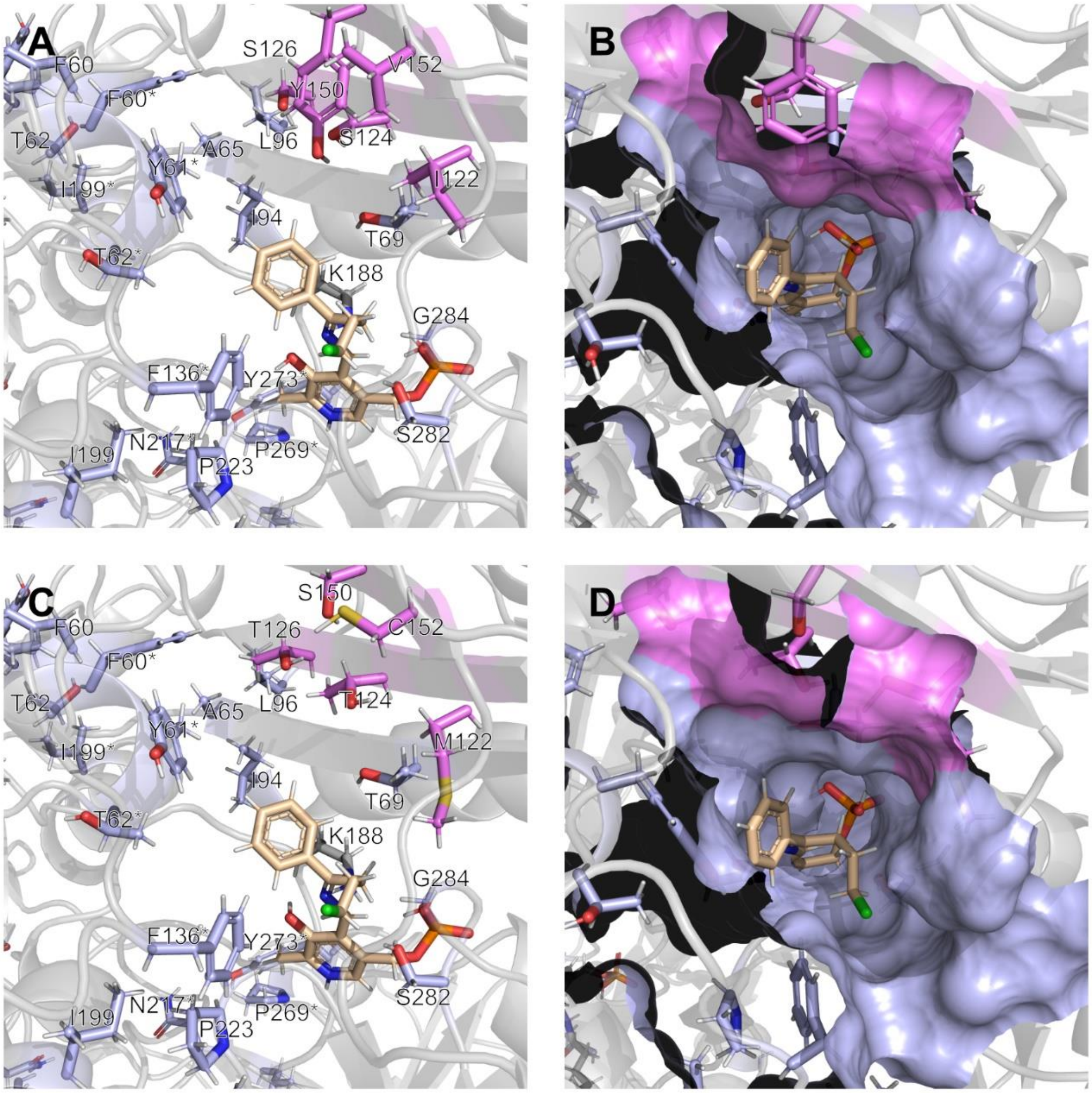

Figure S16: Docked quinonoid intermediate with 4-chlorobutyrophenone **1c** in the active sites of ATA-117-Rd6: **A**: showing residues mutated during the directed evolution; residues differing between ATA-117-Rd6 and -Rd11 are pink while identical residues are blue; **B**: surface of residues in contact with the substrate. ATA-117-Rd11: **C**: showing residues mutated during the directed evolution; residues differing between ATA-117-Rd6 and -Rd11 are pink while identical residues are blue; **D**: surface of residues in contact with the substrate. Residues in other subunits are denoted with an asterisk. Docking was carried out with the dock_run.mcr macro in YASARA 20.12.24, the figure was generated using Open Source PyMOL 2.5.0.

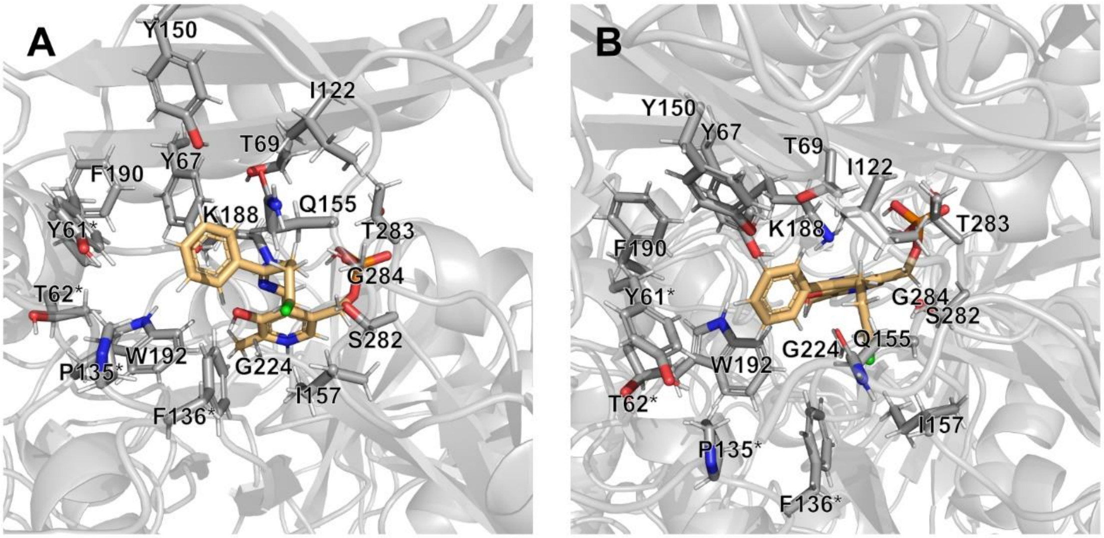

Figure S17: Docked quinonoid intermediate with 4-chlorobutyrophenone **1c** in the active site of ATA-117-Rd6, **A**: side view, **B**: top view. Residues within 4 Å of any of the docked substrates are shown as sticks. Residues in other subunits are denoted with an asterisk. Docking was carried out with the dock_run.mcr macro in YASARA 20.12.24, the figure was generated using Open Source PyMOL 2.5.0.

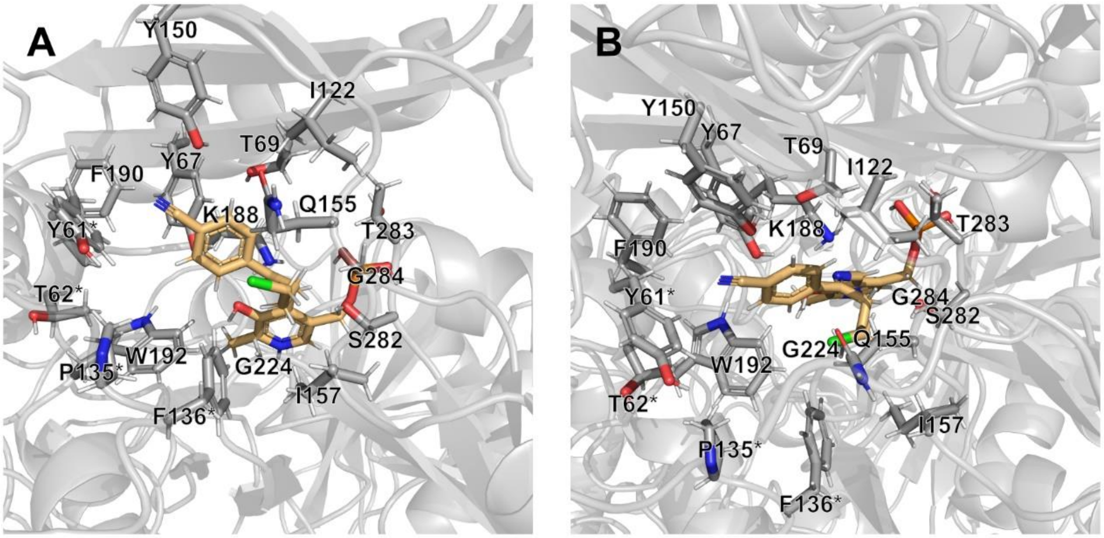

Figure S18: Docked quinonoid intermediate with 4-chlorobutyrophenone **1d** in the active site of ATA-117-Rd6, **A**: side view, **B**: top view. Residues within 4 Å of any of the docked substrates are shown as sticks. Residues in other subunits are denoted with an asterisk. Docking was carried out with the dock_run.mcr macro in YASARA 20.12.24, the figure was generated using Open Source PyMOL 2.5.0.

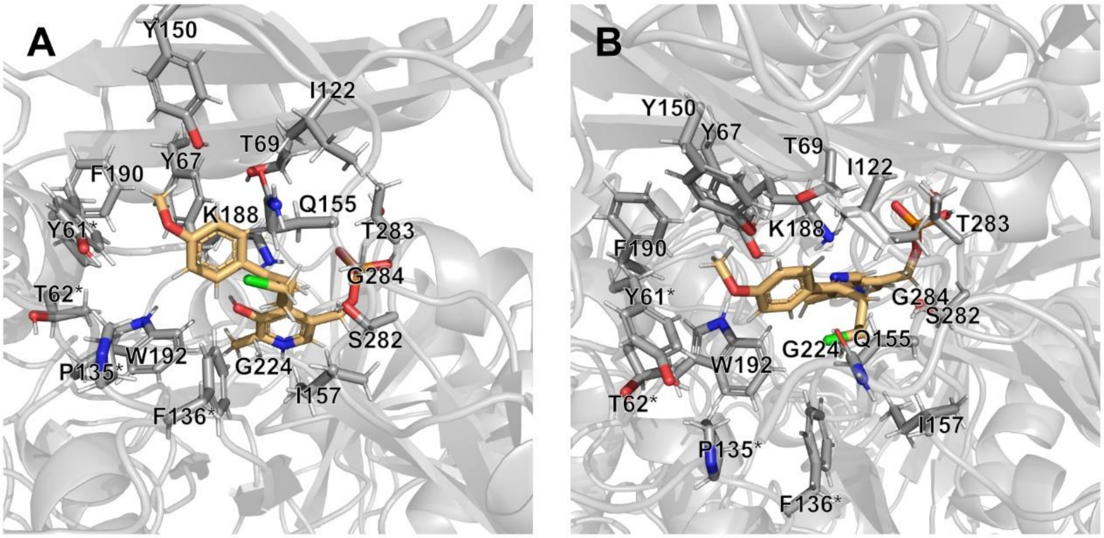

Figure S19: Docked quinonoid intermediate with 4-chlorobutyrophenone **1e** in the active site of ATA-117-Rd6, **A**: side view, **B**: top view. Residues within 4 Å of any of the docked substrates are shown as sticks. Residues in other subunits are denoted with an asterisk. Docking was carried out with the dock_run.mcr macro in YASARA 20.12.24, the figure was generated using Open Source PyMOL 2.5.0.

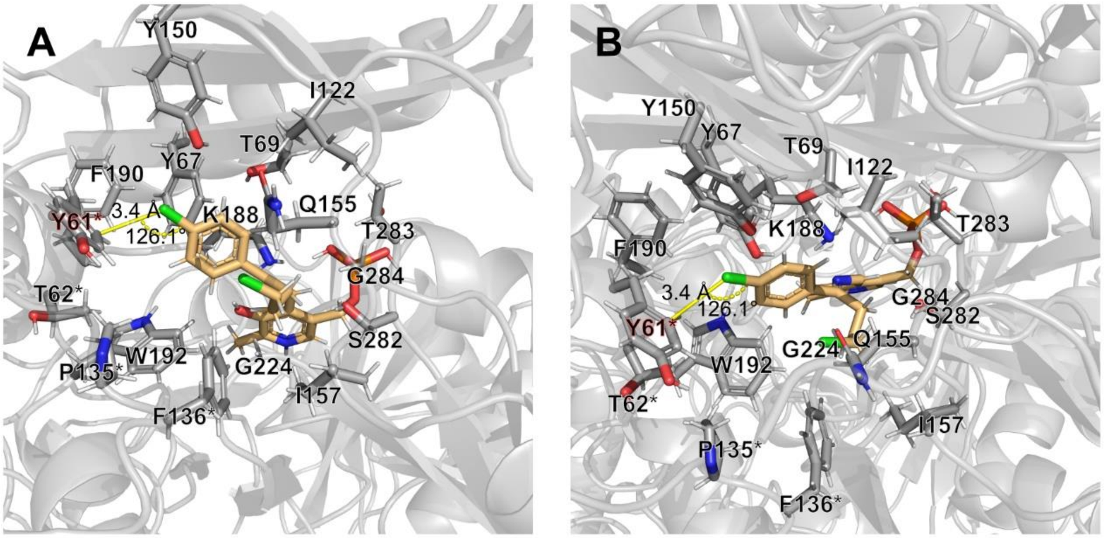

Figure S20: Docked quinonoid intermediate with 4-chlorobutyrophenone **1f** in the active site of ATA-117-Rd6, **A**: side view, **B**: top view. Residues within 4 Å of any of the docked substrates are shown as sticks. Residues in other subunits are denoted with an asterisk. Docking was carried out with the dock_run.mcr macro in YASARA 20.12.24, the figure was generated using Open Source PyMOL 2.5.0.

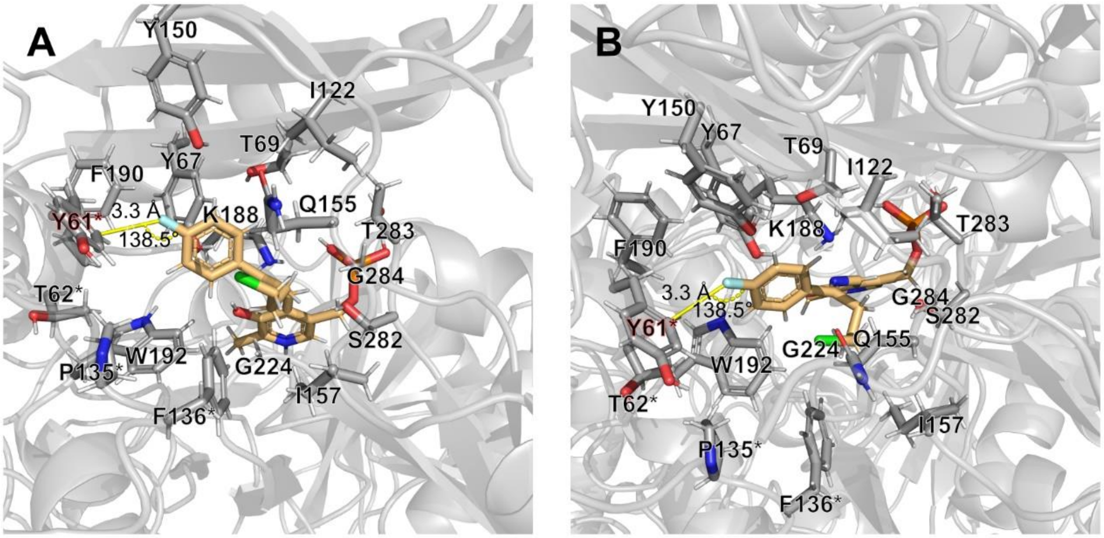

Figure S21: Docked quinonoid intermediate with 4-chlorobutyrophenone **1g** in the active site of ATA-117-Rd6, **A**: side view, **B**: top view. Residues within 4 Å of any of the docked substrates are shown as sticks. Residues in other subunits are denoted with an asterisk. Docking was carried out with the dock_run.mcr macro in YASARA 20.12.24, the figure was generated using Open Source PyMOL 2.5.0.

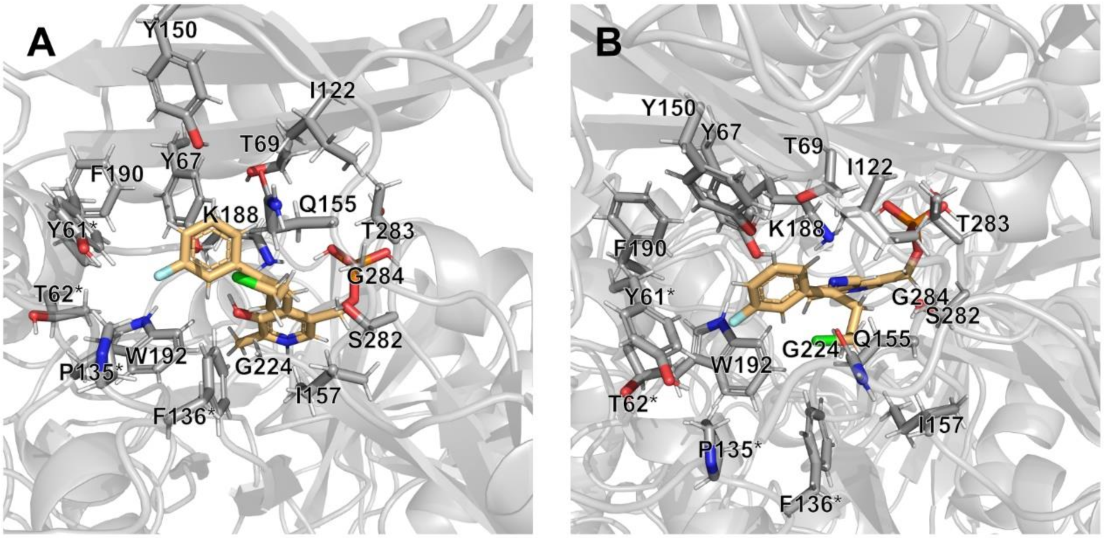

Figure S22: Docked quinonoid intermediate with 4-chlorobutyrophenone **1h** in the active site of ATA-117-Rd6, **A**: side view, **B**: top view. Residues within 4 Å of any of the docked substrates are shown as sticks. Residues in other subunits are denoted with an asterisk. Docking was carried out with the dock_run.mcr macro in YASARA 20.12.24, the figure was generated using Open Source PyMOL 2.5.0.

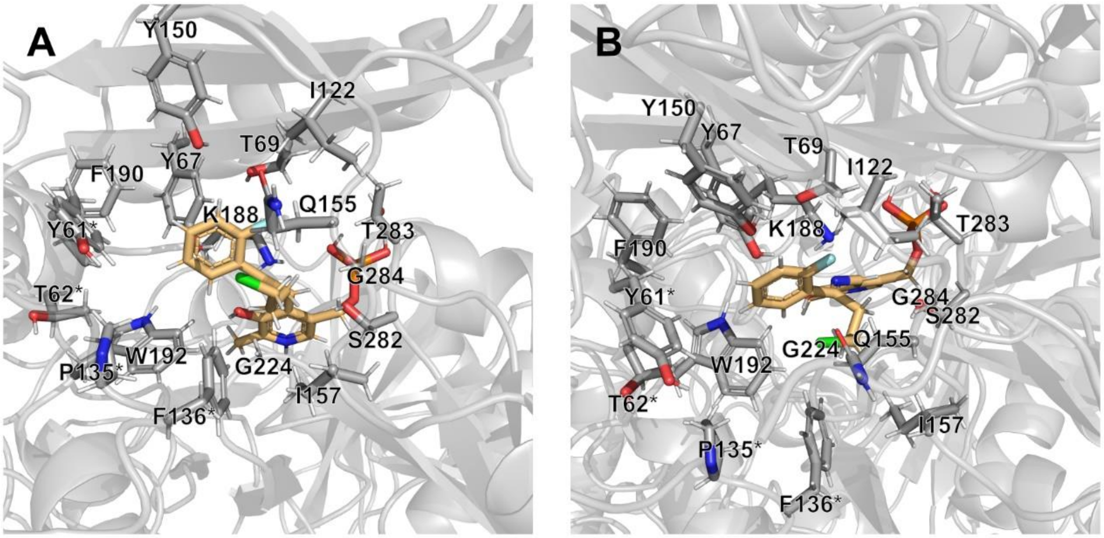

Figure S23: Docked quinonoid intermediate with 4-chlorobutyrophenone **1i** in the active site of ATA-117-Rd6, **A**: side view, **B**: top view. Residues within 4 Å of any of the docked substrates are shown as sticks. Residues in other subunits are denoted with an asterisk. Docking was carried out with the dock_run.mcr macro in YASARA 20.12.24, the figure was generated using Open Source PyMOL 2.5.0.

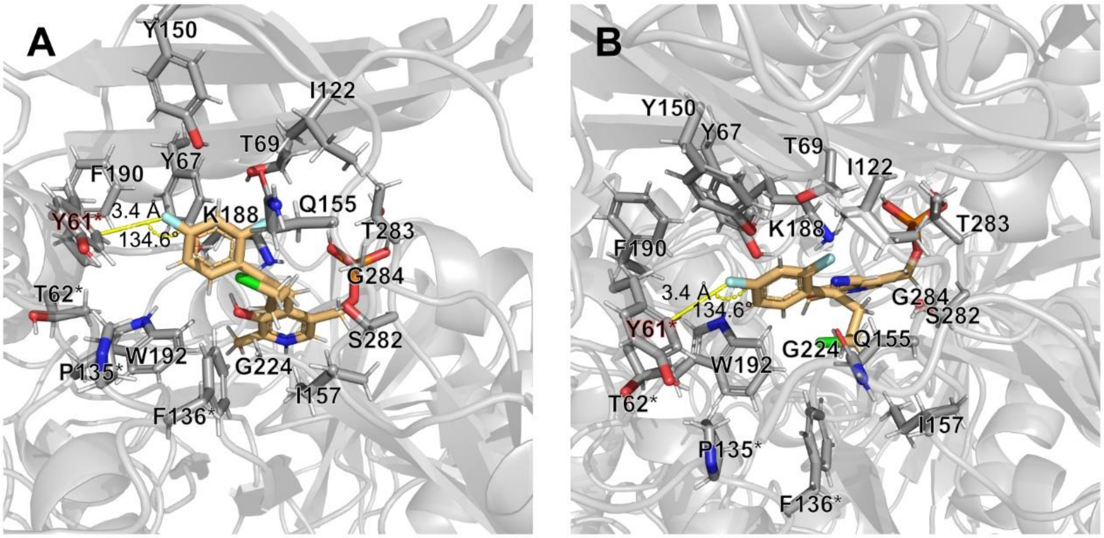

Figure S24: Docked quinonoid intermediate with 4-chlorobutyrophenone **1j** in the active site of ATA-117-Rd6, **A**: side view, **B**: top view. Residues within 4 Å of any of the docked substrates are shown as sticks. Residues in other subunits are denoted with an asterisk. Docking was carried out with the dock_run.mcr macro in YASARA 20.12.24, the figure was generated using Open Source PyMOL 2.5.0.

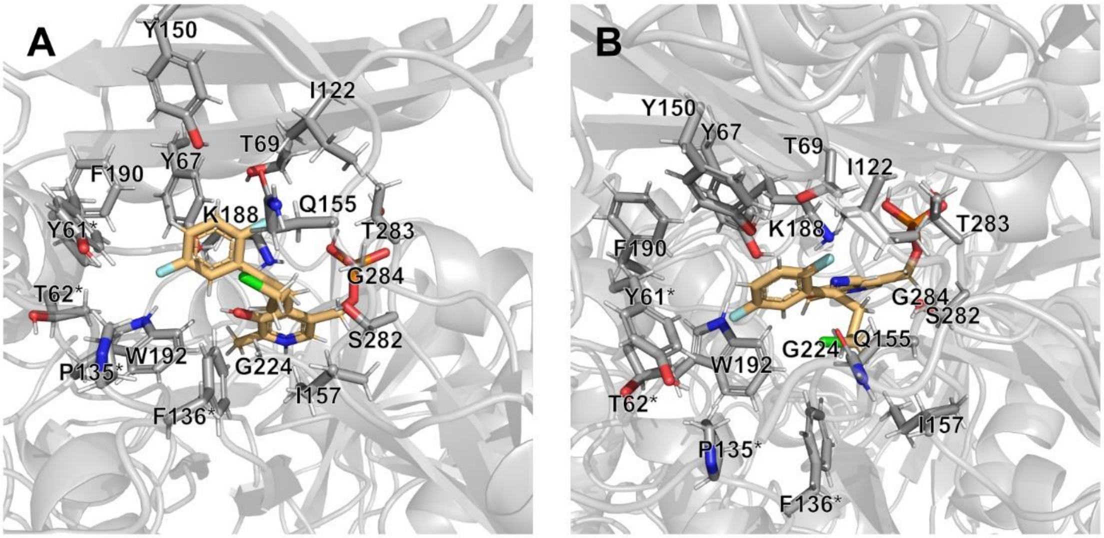

Figure S25: Docked quinonoid intermediate with 4-chlorobutyrophenone **1k** in the active site of ATA-117-Rd6, **A**: side view, **B**: top view. Residues within 4 Å of any of the docked substrates are shown as sticks. Residues in other subunits are denoted with an asterisk. Docking was carried out with the dock_run.mcr macro in YASARA 20.12.24, the figure was generated using Open Source PyMOL 2.5.0.

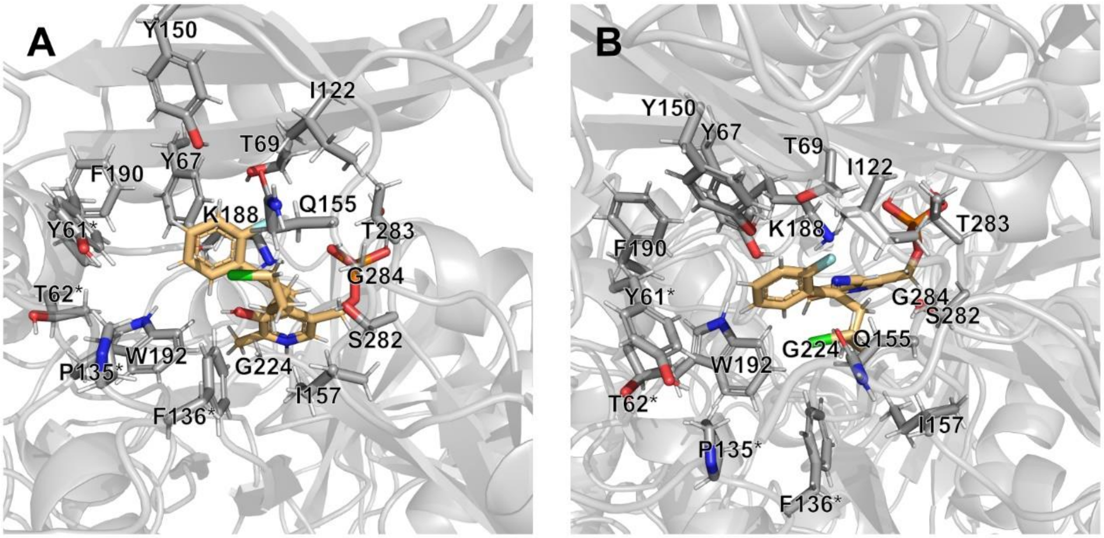

Figure S26: Docked quinonoid intermediate with 5-chlorovalerophenone **1l** in the active site of ATA-117-Rd6, **A**: side view, **B**: top view. Residues within 4 Å of any of the docked substrates are shown as sticks. Residues in other subunits are denoted with an asterisk. Docking was carried out with the dock_run.mcr macro in YASARA 20.12.24, the figure was generated using Open Source PyMOL 2.5.0.

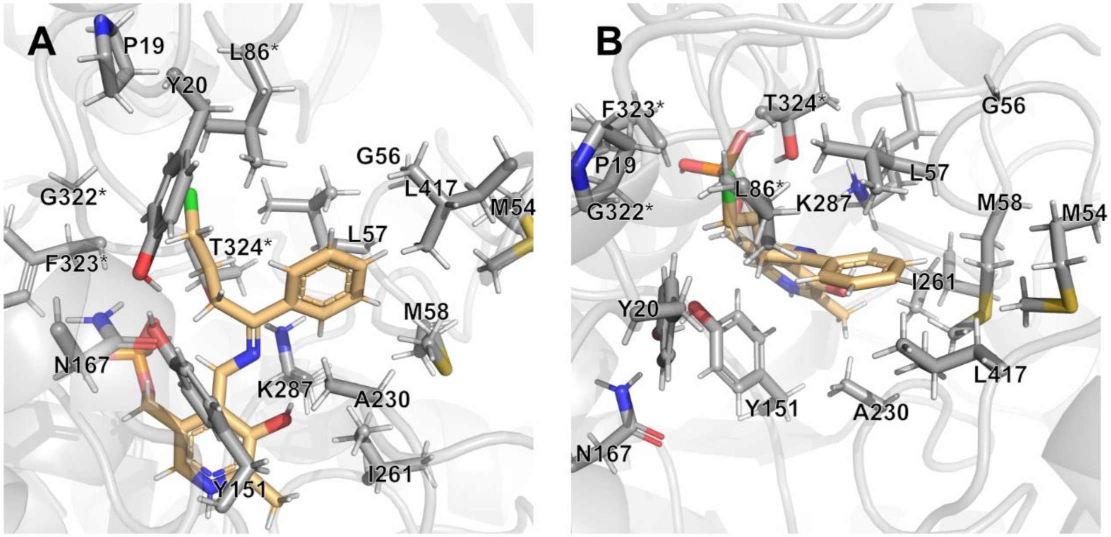

Figure S27: Docked quinonoid intermediate with 4-chlorobutyrophenone **1c** in the active site of *Pj*STA-R6-8, **A**: side view, **B**: top view. Residues within 4 Å of any of the docked substrates are shown as sticks. Residues in other subunits are denoted with an asterisk. Docking was carried out with the dock_run.mcr macro in YASARA 20.12.24, the figure was generated using Open Source PyMOL 2.5.0.

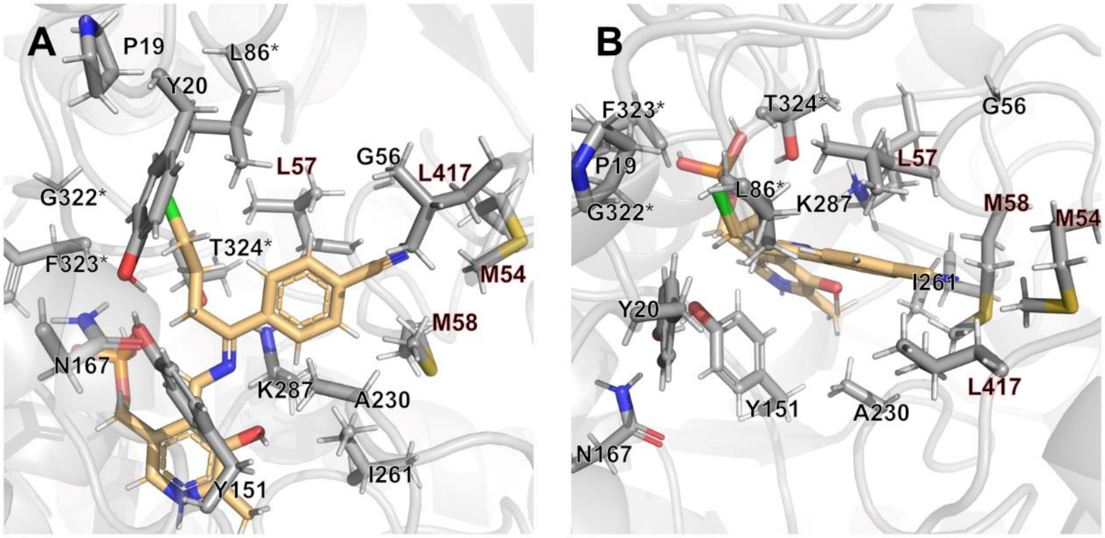

Figure S28: Docked quinonoid intermediate with 4-chlorobutyrophenone **1d** in the active site of *Pj*STA-R6-8, **A**: side view, **B**: top view. Residues within 4 Å of any of the docked substrates are shown as sticks. Residues in other subunits are denoted with an asterisk. Docking was carried out with the dock_run.mcr macro in YASARA 20.12.24, the figure was generated using Open Source PyMOL 2.5.0.

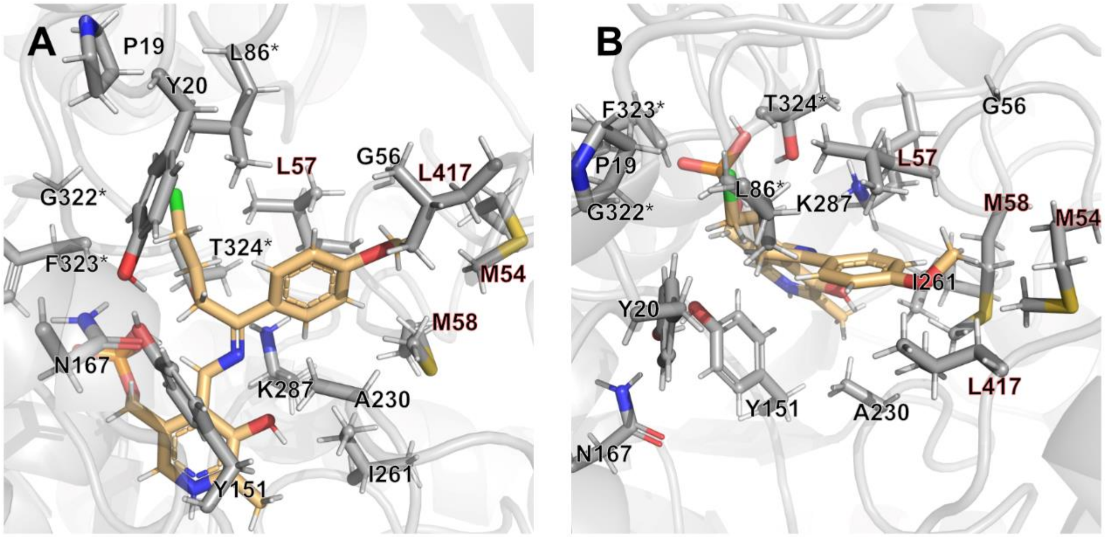

Figure S29: Docked quinonoid intermediate with 4-chlorobutyrophenone **1e** in the active site of *Pj*STA-R6-8, **A**: side view, **B**: top view. Residues within 4 Å of any of the docked substrates are shown as sticks. Residues in other subunits are denoted with an asterisk. Docking was carried out with the dock_run.mcr macro in YASARA 20.12.24, the figure was generated using Open Source PyMOL 2.5.0.

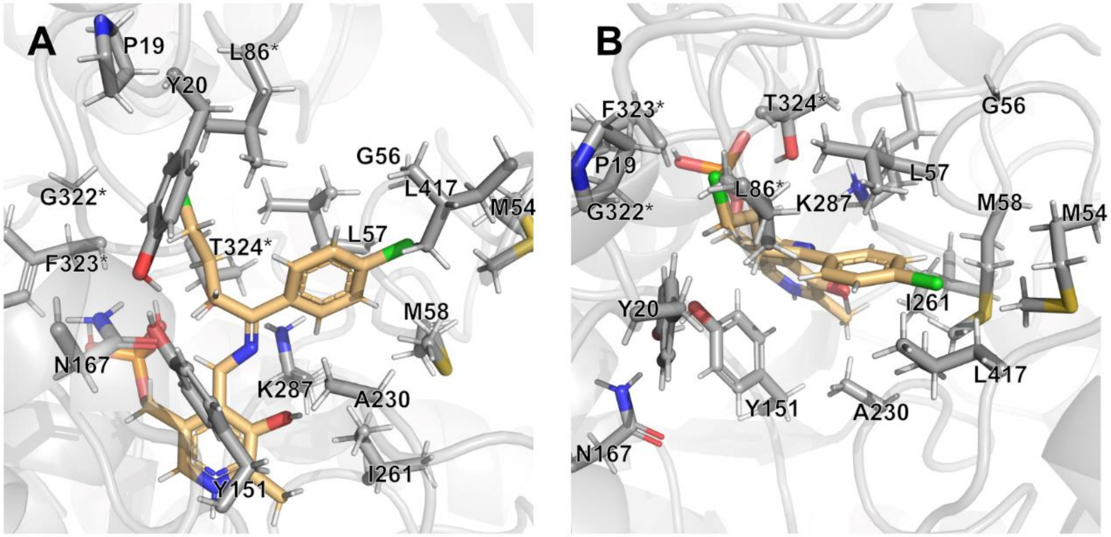

Figure S30: Docked quinonoid intermediate with 4-chlorobutyrophenone **1f** in the active site of *Pj*STA-R6-8, **A**: side view, **B**: top view. Residues within 4 Å of any of the docked substrates are shown as sticks. Residues in other subunits are denoted with an asterisk. Docking was carried out with the dock_run.mcr macro in YASARA 20.12.24, the figure was generated using Open Source PyMOL 2.5.0.

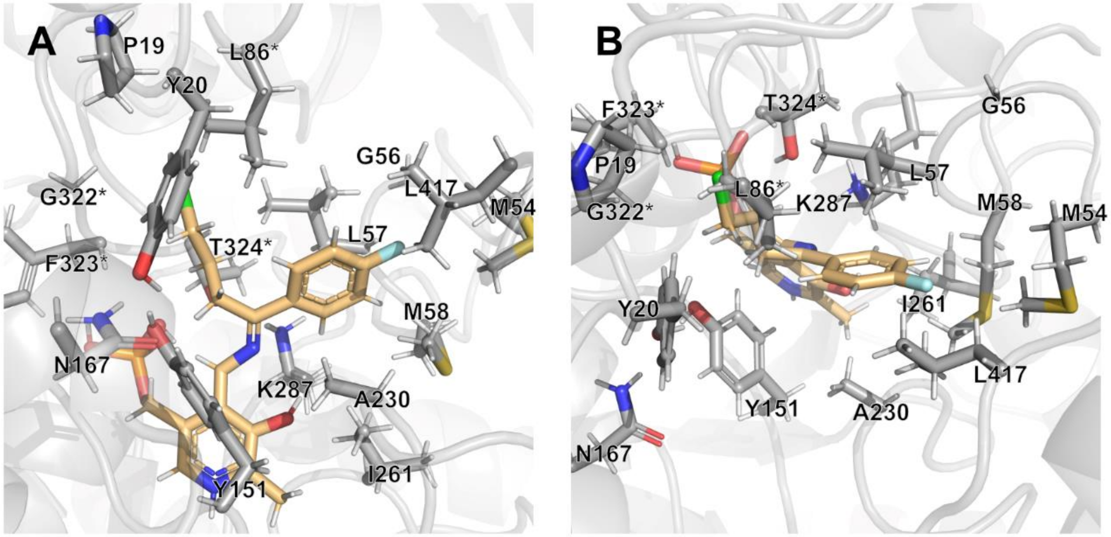

Figure S31: Docked quinonoid intermediate with 4-chlorobutyrophenone **1g** in the active site of *Pj*STA-R6-8, **A**: side view, **B**: top view. Residues within 4 Å of any of the docked substrates are shown as sticks. Residues in other subunits are denoted with an asterisk. Docking was carried out with the dock_run.mcr macro in YASARA 20.12.24, the figure was generated using Open Source PyMOL 2.5.0.

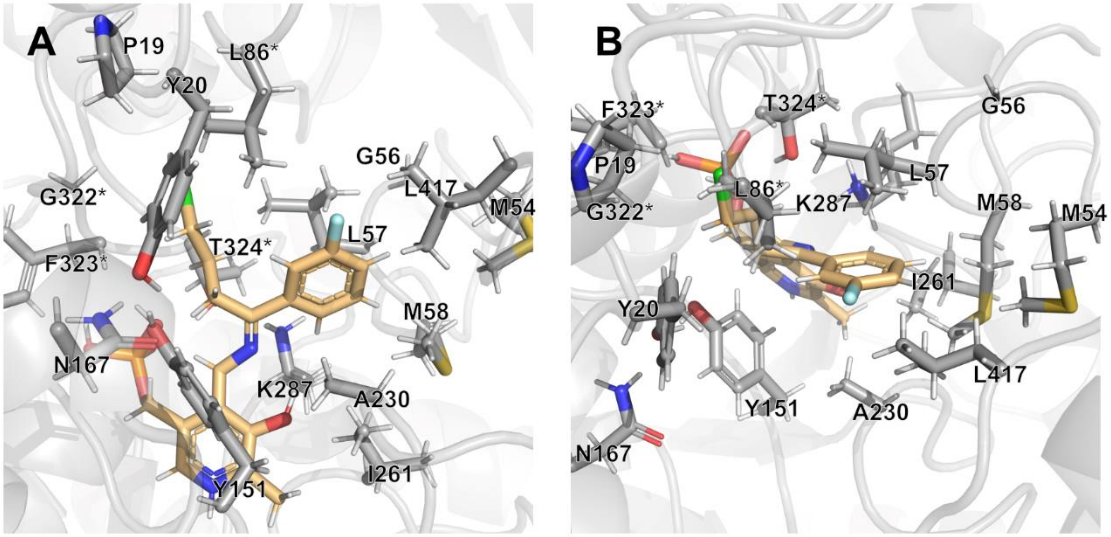

Figure S32: Docked quinonoid intermediate with 4-chlorobutyrophenone **1h** in the active site of *Pj*STA-R6-8, **A**: side view, **B**: top view. Residues within 4 Å of any of the docked substrates are shown as sticks. Residues in other subunits are denoted with an asterisk. Docking was carried out with the dock_run.mcr macro in YASARA 20.12.24, the figure was generated using Open Source PyMOL 2.5.0.

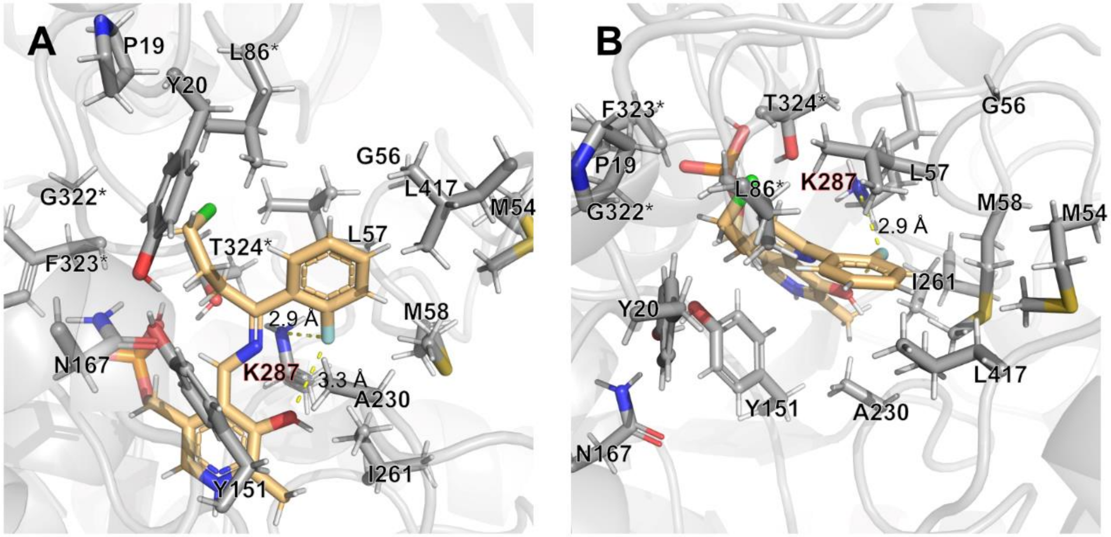

Figure S33: Docked quinonoid intermediate with 4-chlorobutyrophenone **1i** in the active site of *Pj*STA-R6-8, **A**: side view, **B**: top view. Residues within 4 Å of any of the docked substrates are shown as sticks. Residues in other subunits are denoted with an asterisk. Docking was carried out with the dock_run.mcr macro in YASARA 20.12.24, the figure was generated using Open Source PyMOL 2.5.0.

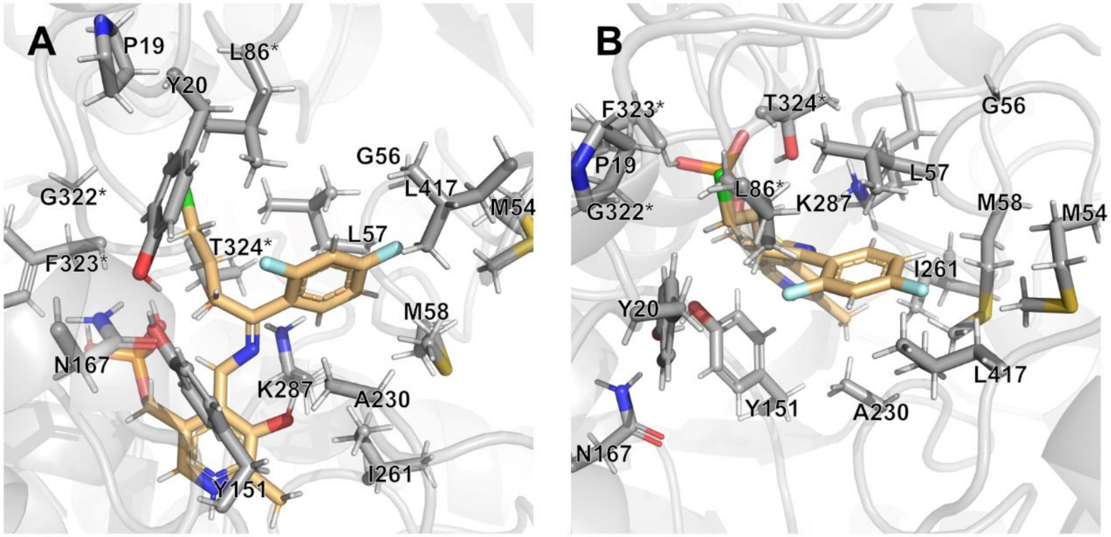

Figure S34: Docked quinonoid intermediate with 4-chlorobutyrophenone **1j** in the active site of *Pj*STA-R6-8, **A**: side view, **B**: top view. Residues within 4 Å of any of the docked substrates are shown as sticks. Residues in other subunits are denoted with an asterisk. Docking was carried out with the dock_run.mcr macro in YASARA 20.12.24, the figure was generated using Open Source PyMOL 2.5.0.

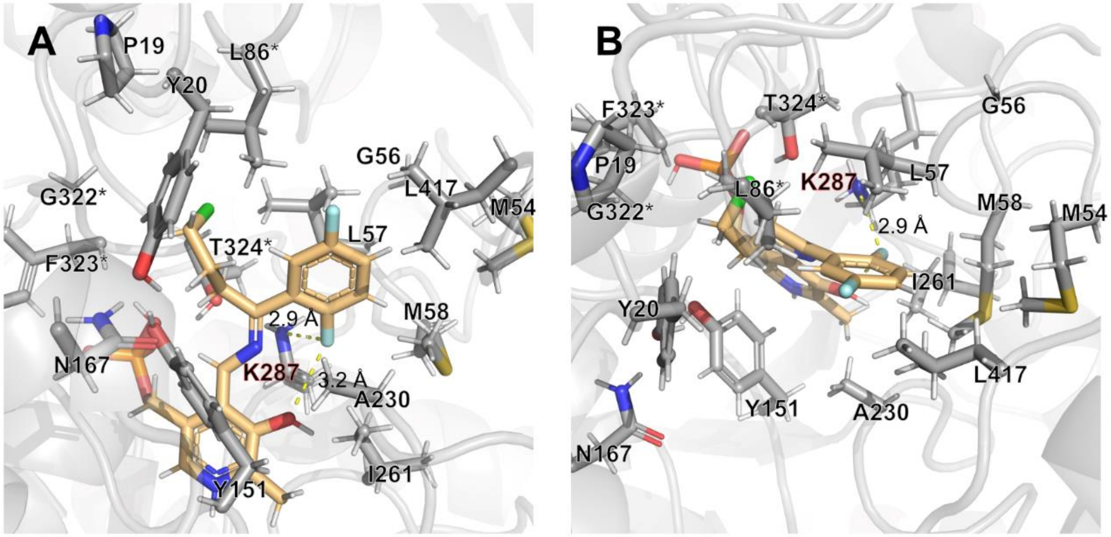

Figure S35: Docked quinonoid intermediate with 4-chlorobutyrophenone **1k** in the active site of *Pj*STA-R6-8, **A**: side view, **B**: top view. Residues within 4 Å of any of the docked substrates are shown as sticks. Residues in other subunits are denoted with an asterisk. Docking was carried out with the dock_run.mcr macro in YASARA 20.12.24, the figure was generated using Open Source PyMOL 2.5.0.

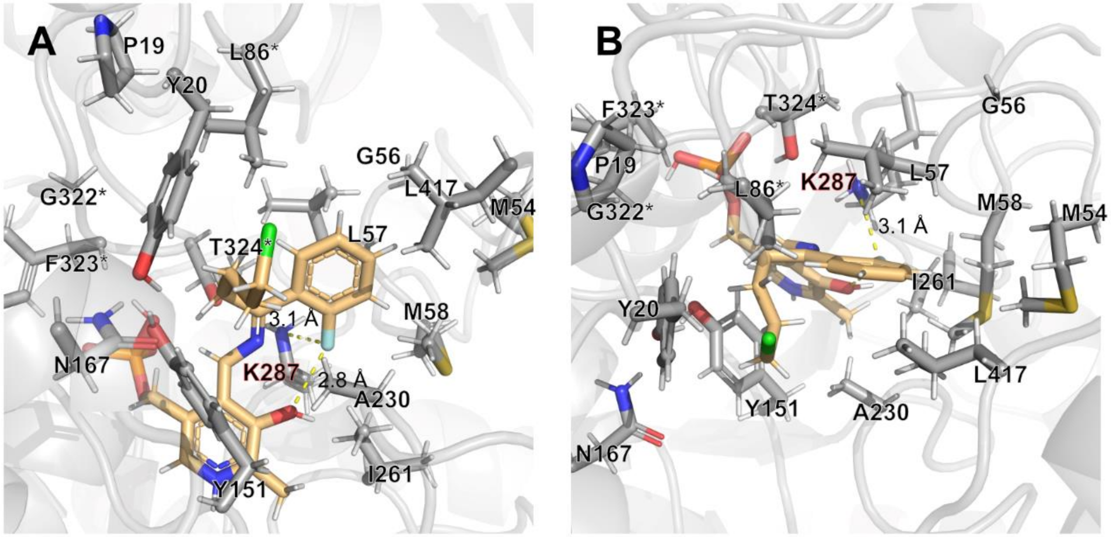

Figure S36: Docked quinonoid intermediate with 5-chlorovalerophenone **1l** in the active site of *Pj*STA-R6-8, **A**: side view, **B**: top view. Residues within 4 Å of any of the docked substrates are shown as sticks. Residues in other subunits are denoted with an asterisk. Docking was carried out with the dock_run.mcr macro in YASARA 20.12.24, the figure was generated using Open Source PyMOL 2.5.0.

Chromatograms

*GC-FID chromatogram of biotransformations of **1a***

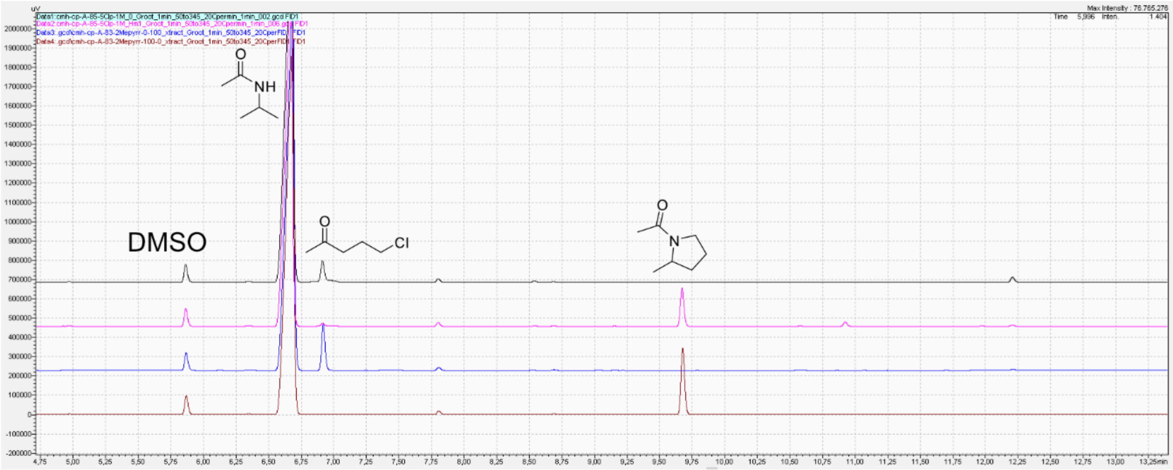

Black: no enzyme, pink: HEwT W56G, blue: commercial standard of **1a**, brown: commercial standard of (acetylated) **3a**

*GC-FID chromatogram of biotransformations of **1b***

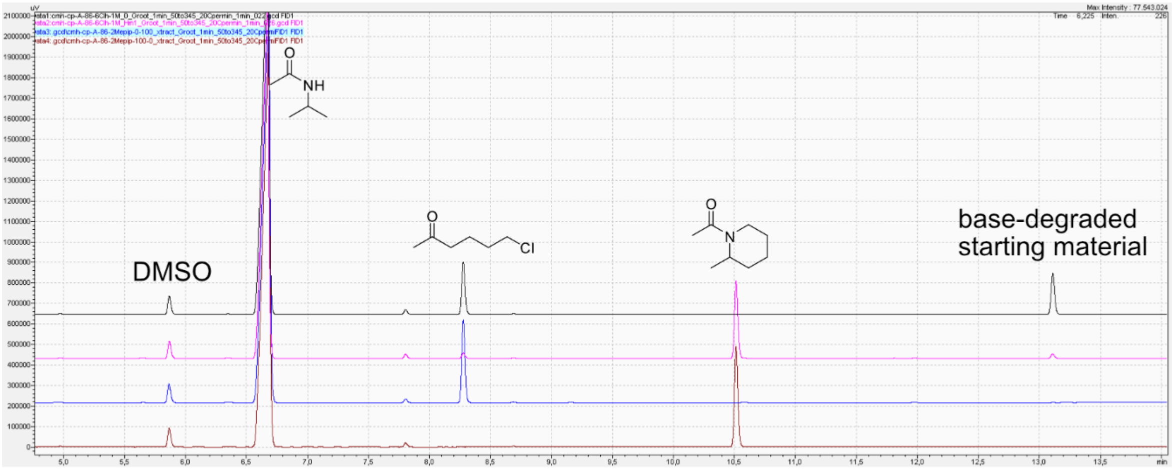

Black: no enzyme, pink: HEwT W56G, blue: commercial standard of **1b**, brown: commercial standard of (acetylated) **3b**

*HPLC chromatogram of biotransformations of **1c***

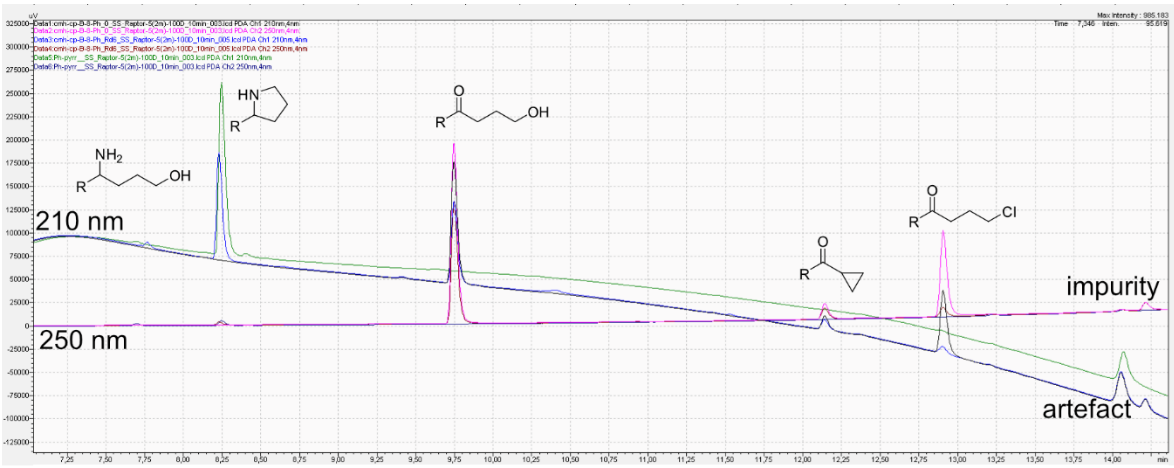

Black (210 nm), pink (250 nm): no enzyme, blue (210 nm), brown (250 nm): ATA-117-Rd6, Green (210 nm), dark blue (250 nm): commercial standard of **3c**

*HPLC chromatogram of biotransformations of **1d***

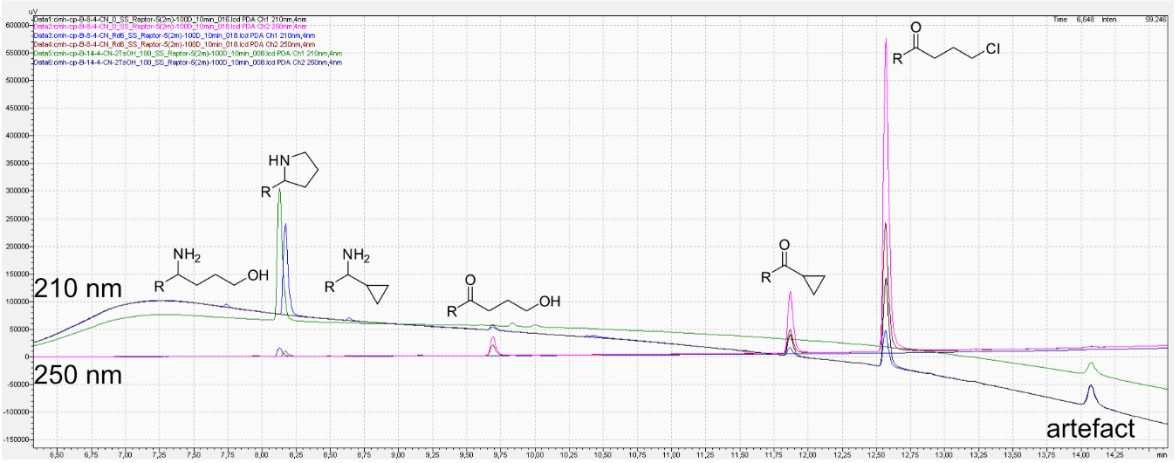

Black (210 nm), pink (250 nm): no enzyme, blue (210 nm), brown (250 nm): ATA-117-Rd6, Green (210 nm), dark blue (250 nm): synthesized standard of **3d**

*HPLC chromatogram of biotransformations of **1e***

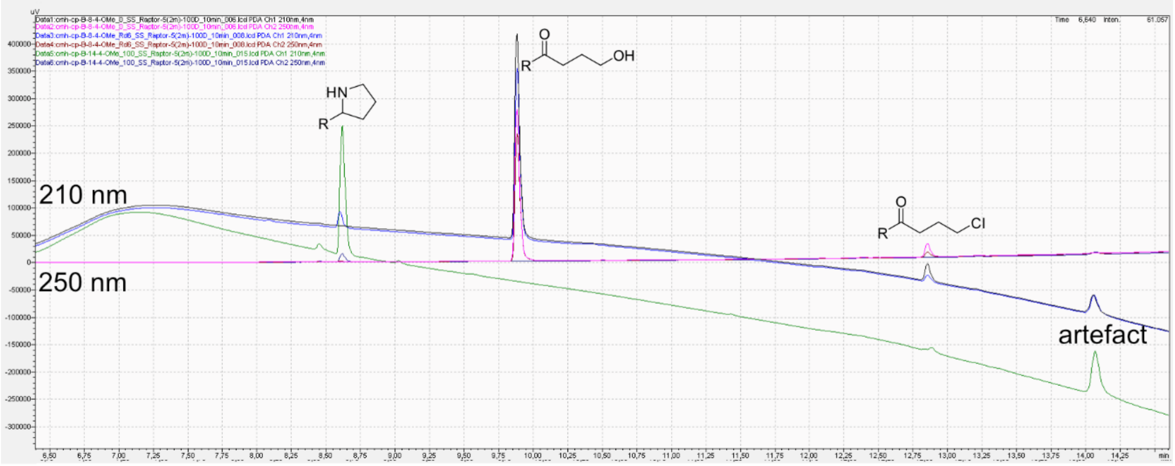

Black (210 nm), pink (250 nm): no enzyme, blue (210 nm), brown (250 nm): ATA-117-Rd6, Green (210 nm), dark blue (250 nm): commercial standard of **3e**

*HPLC chromatogram of biotransformations of **1f***

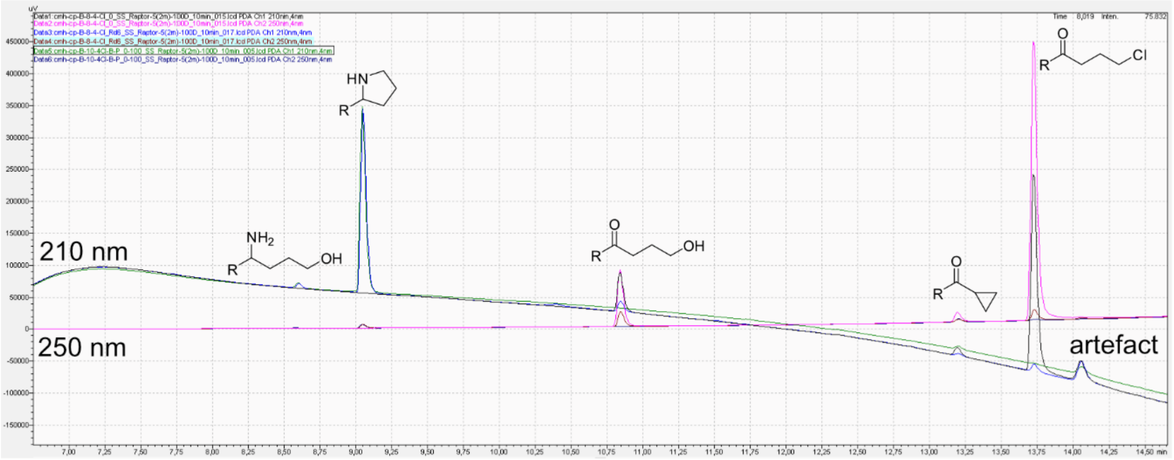

Black (210 nm), pink (250 nm): no enzyme, blue (210 nm), brown (250 nm): ATA-117-Rd6, Green (210 nm), dark blue (250 nm): isolated **3f** from preparative biotransformation

HPLC chromatogram of biotransformations of ***1g***

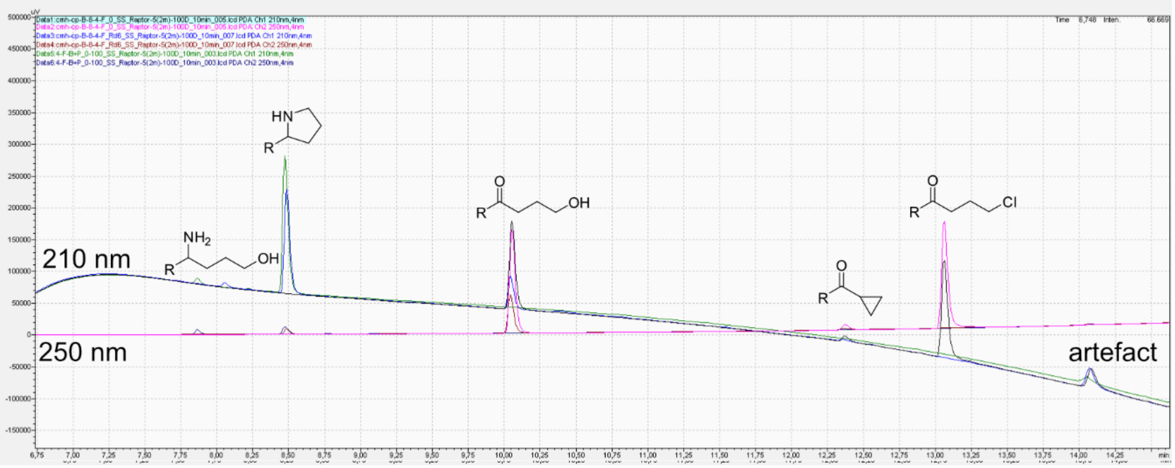

Black (210 nm), pink (250 nm): no enzyme, blue (210 nm), brown (250 nm): ATA-117-Rd6, Green (210 nm), dark blue (250 nm): commercial standard of **3g**

HPLC chromatogram of biotransformations of ***1h***

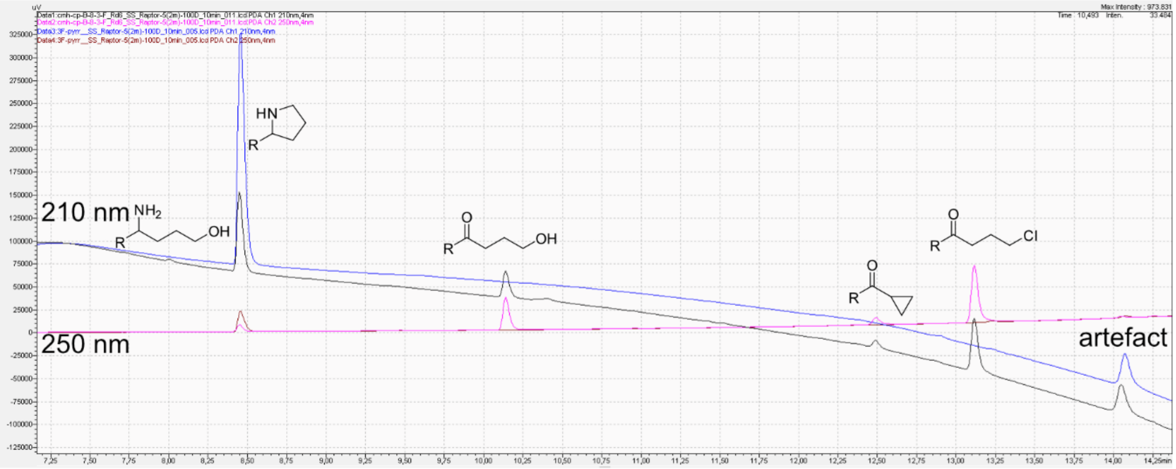

Black (210 nm), pink (250 nm): ATA-117-Rd6, blue (210 nm), brown (250 nm): commercial standard of **3h**

HPLC chromatogram of biotransformations of ***1i***

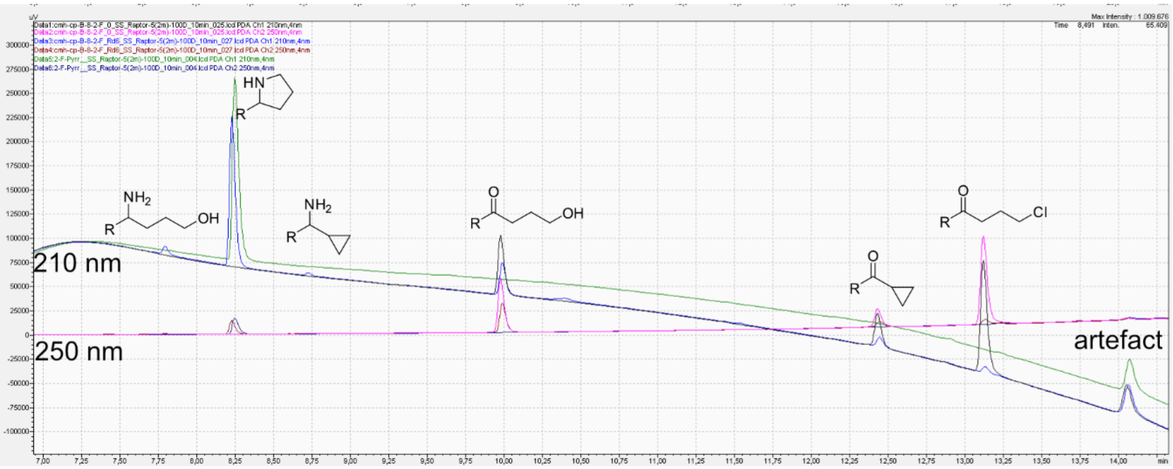

Black (210 nm), pink (250 nm): no enzyme, blue (210 nm), brown (250 nm): ATA-117-Rd6, Green (210 nm), dark blue (250 nm): commercial standard of **3i**

*HPLC chromatogram of biotransformations of **1j***

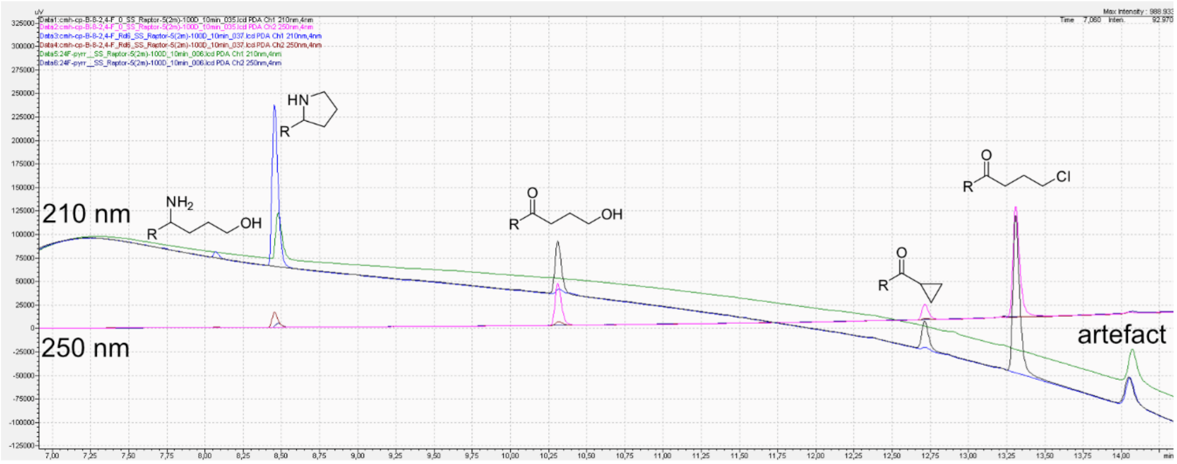

Black (210 nm), pink (250 nm): no enzyme, blue (210 nm), brown (250 nm): ATA-117-Rd6, Green (210 nm), dark blue (250 nm): commercial standard of **3j**

*HPLC chromatogram of biotransformations of **1k***

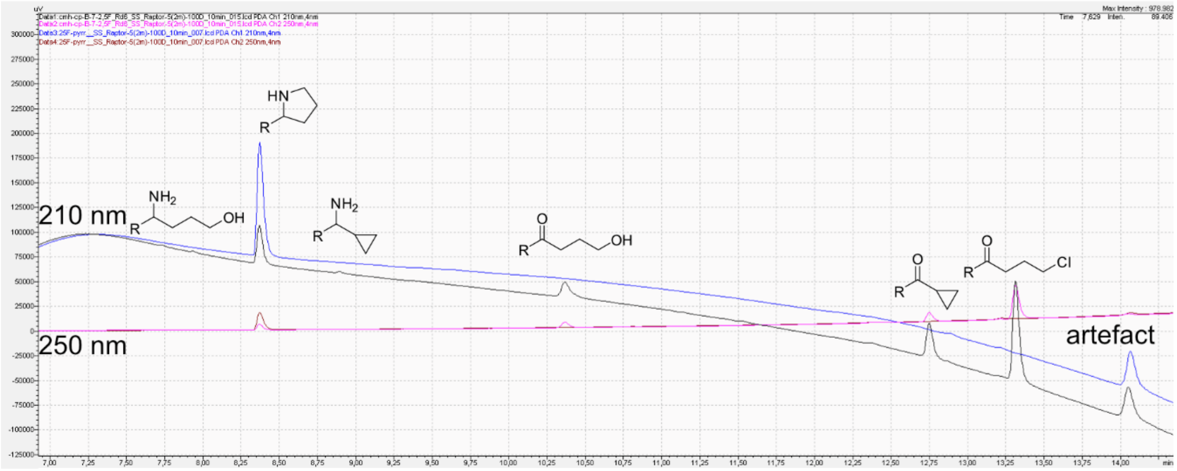

Black (210 nm), pink (250 nm): ATA-117-Rd6, blue (210 nm), brown (250 nm): commercial standard of **3k**

*HPLC chromatogram of biotransformations of **1l***

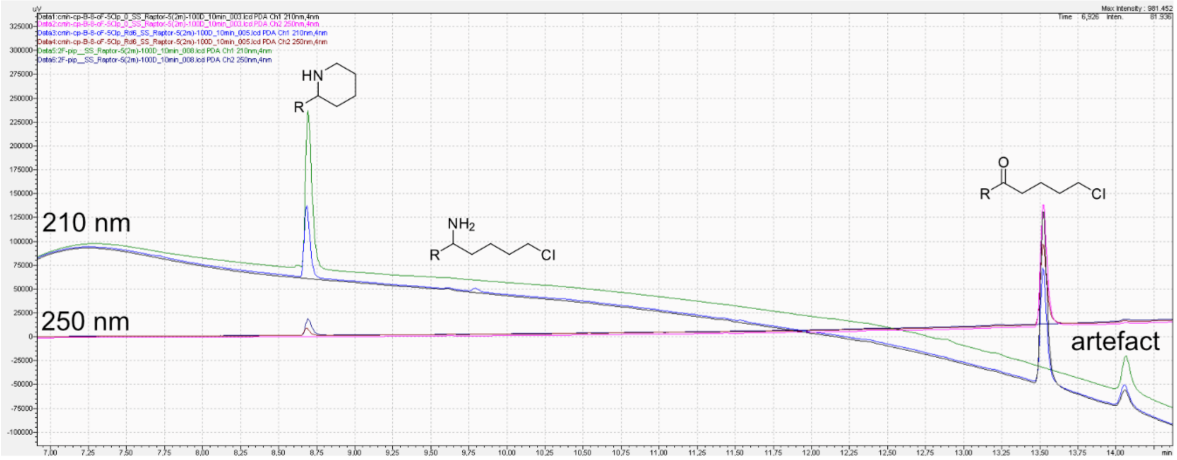

Black (210 nm), pink (250 nm): no enzyme, blue (210 nm), brown (250 nm): ATA-117-Rd6, Green (210 nm), dark blue (250 nm): commercial standard of **3l**

HPLC chromatogram of biotransformations of ***1m***

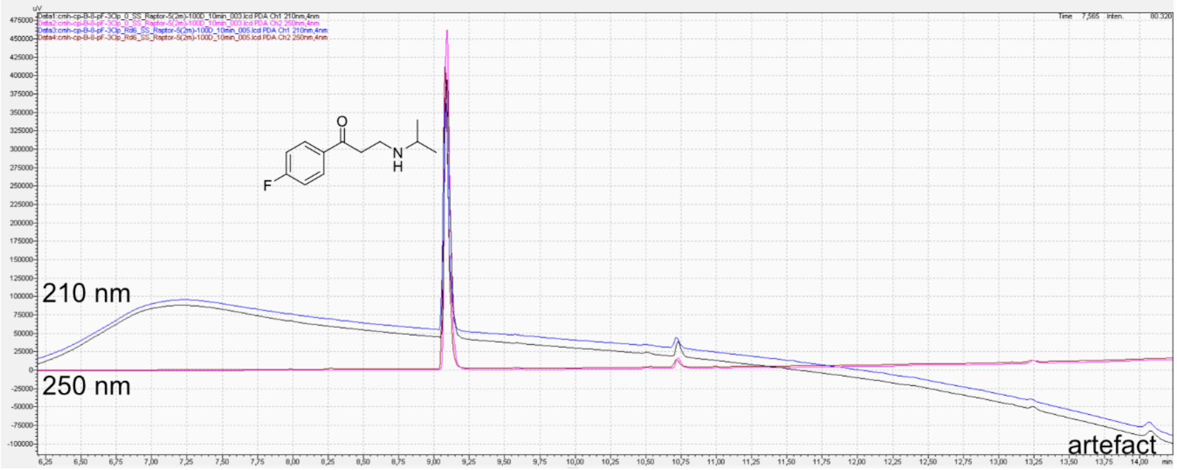

Black (210 nm), pink (250 nm): no enzyme, blue (210 nm), brown (250 nm): ATA-117-Rd6

Extracted ion count (***3c***) LC-MS trace of biotransformations of **1c**

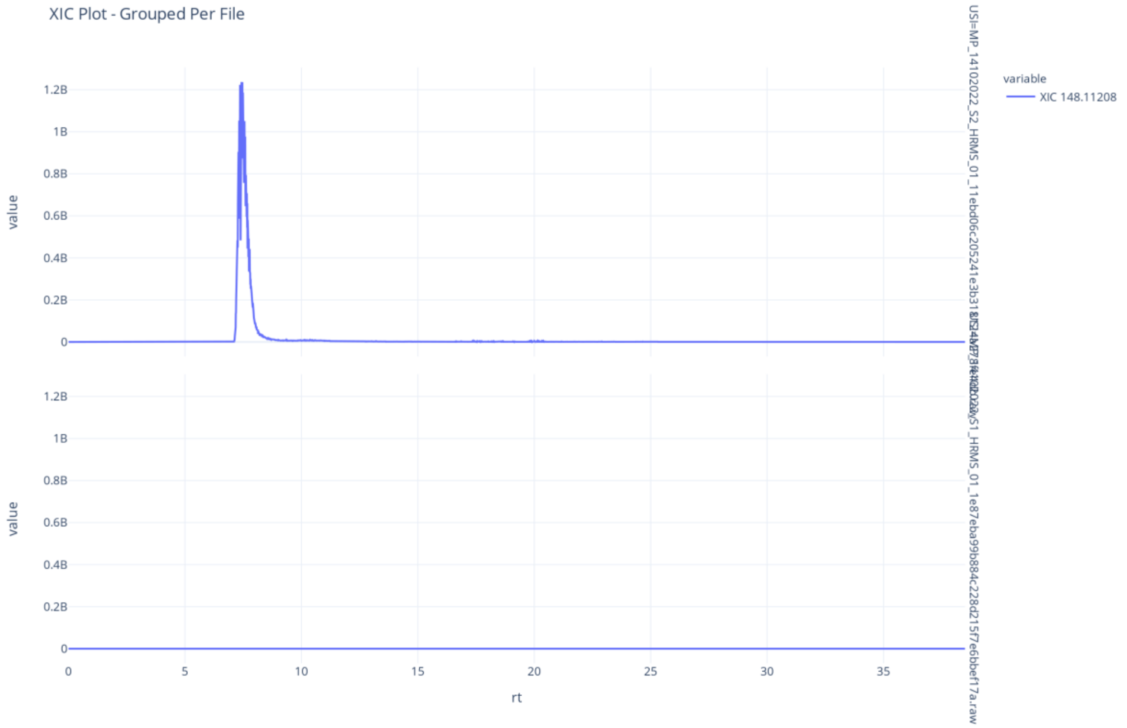

Top: ATA-117-Rd6, Bottom: no enzyme

Extracted ion count (*cyclopropyl aryl ketone*) LC-MS trace of biotransformations of **1c**

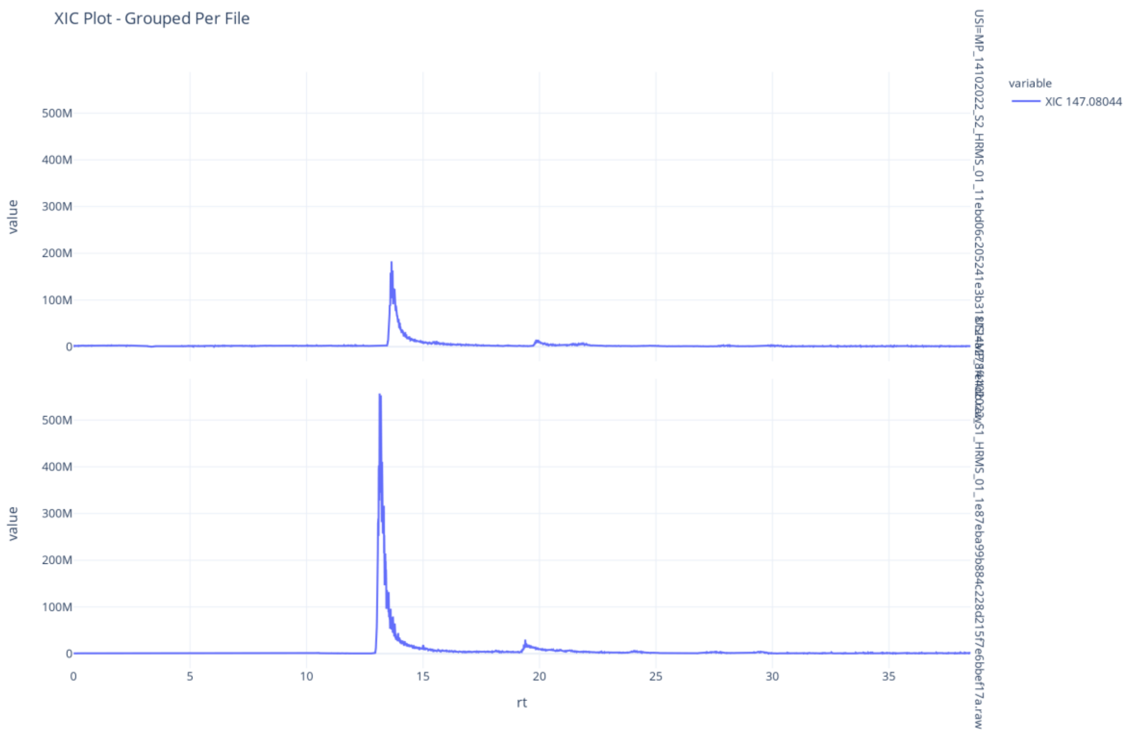

Top: ATA-117-Rd6, Bottom: no enzyme

Extracted ion count (*ω-hydroxy ketone*) LC-MS trace of biotransformations of **1c**

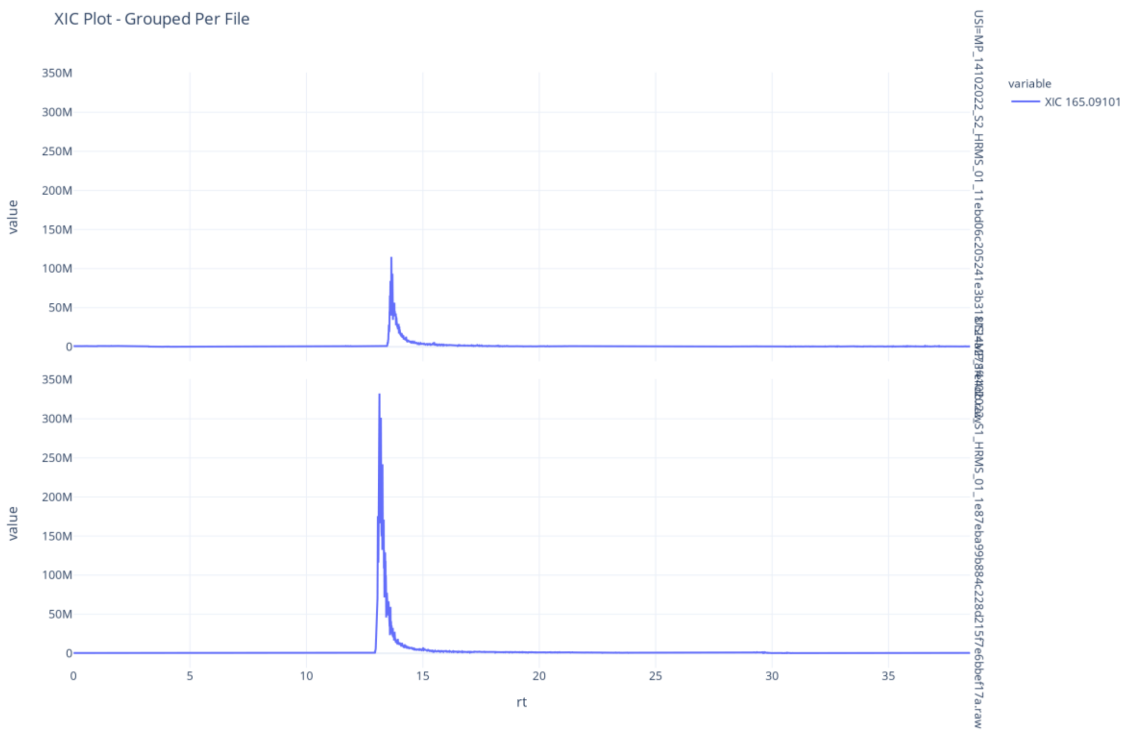

Top: ATA-117-Rd6, Bottom: no enzyme

Extracted ion count (**1c**) LC-MS trace of biotransformations of **1c**

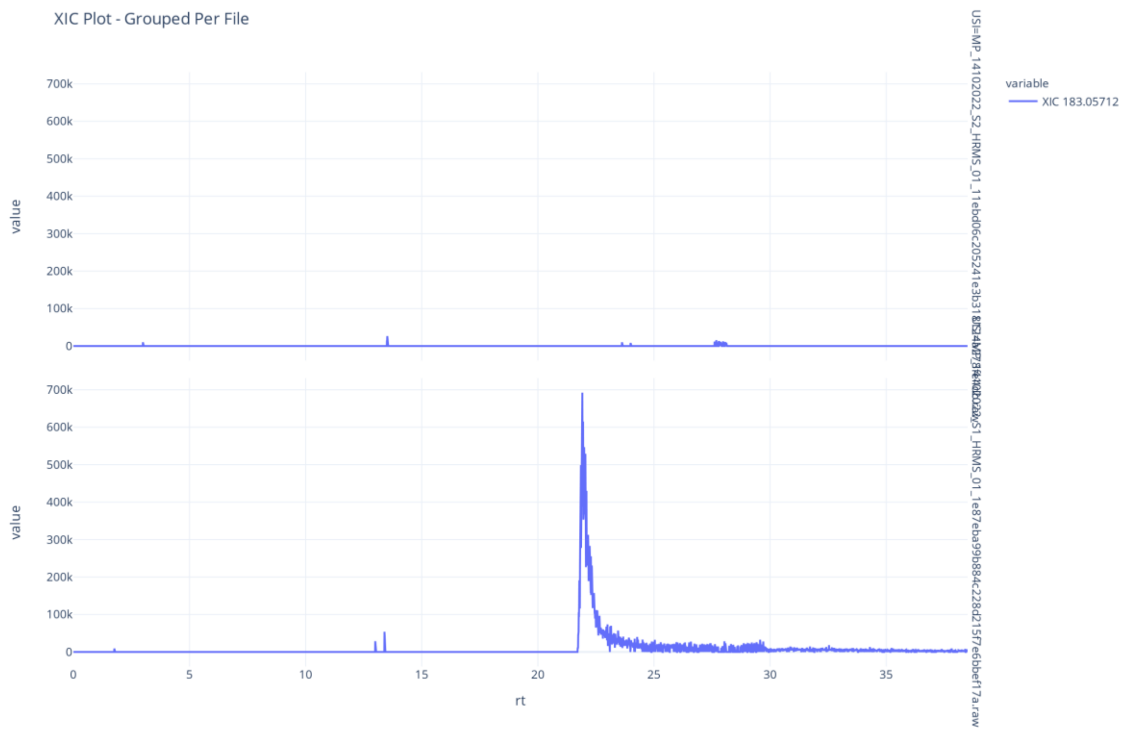

Top: ATA-117-Rd6, Bottom: no enzyme

Extracted ion count (**2c**) LC-MS trace of biotransformations of **1c**

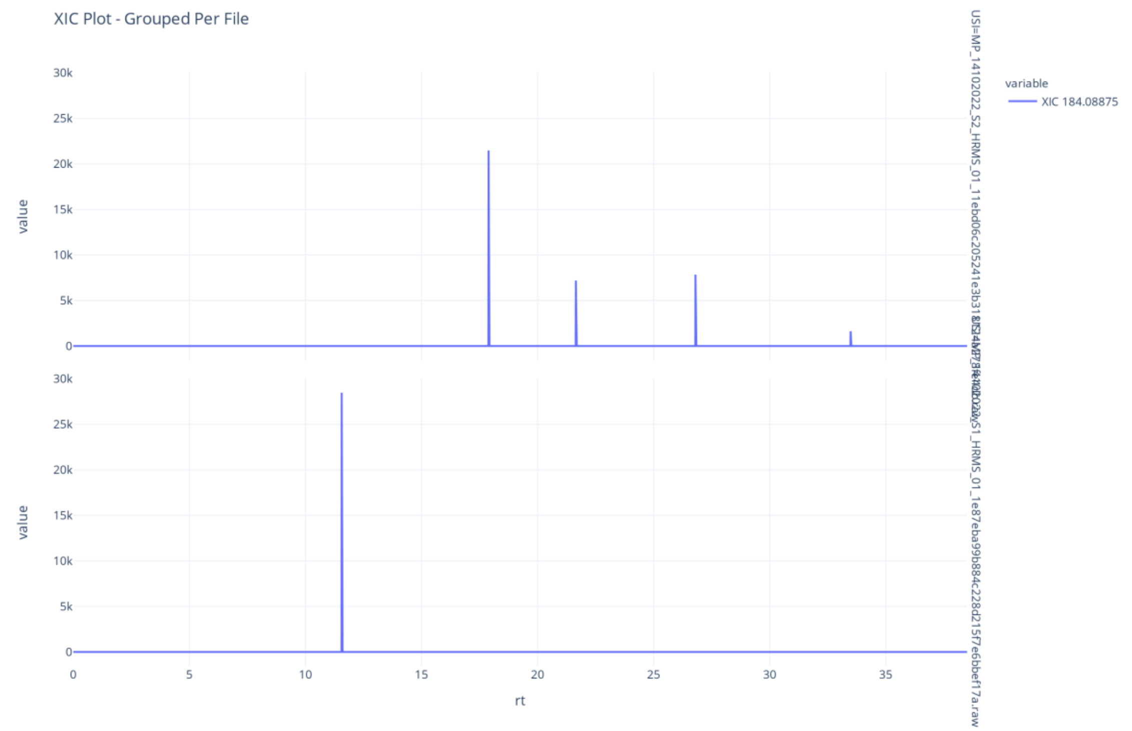

Top: ATA-117-Rd6, Bottom: no enzyme

Extracted ion count (*ω-hydroxy amine*) LC-MS trace of biotransformations of **1c**

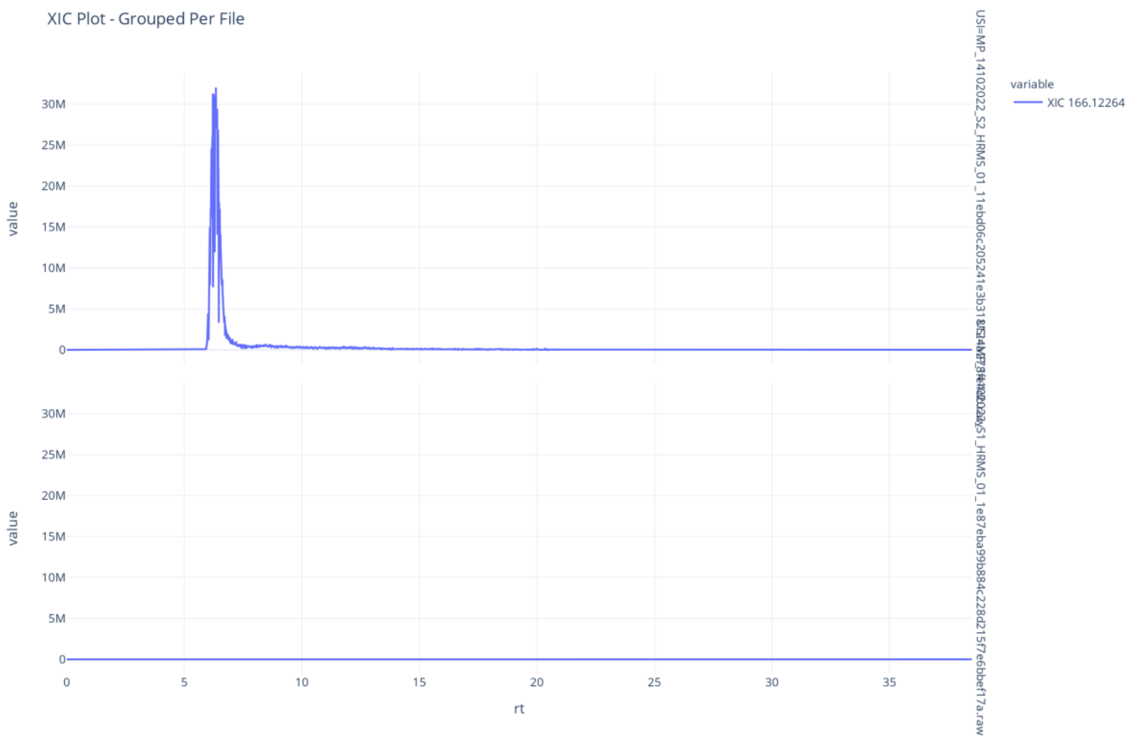

Top: ATA-117-Rd6, Bottom: no enzyme

*Extracted ion count (**3d**) LC-MS trace of biotransformations of **1d***

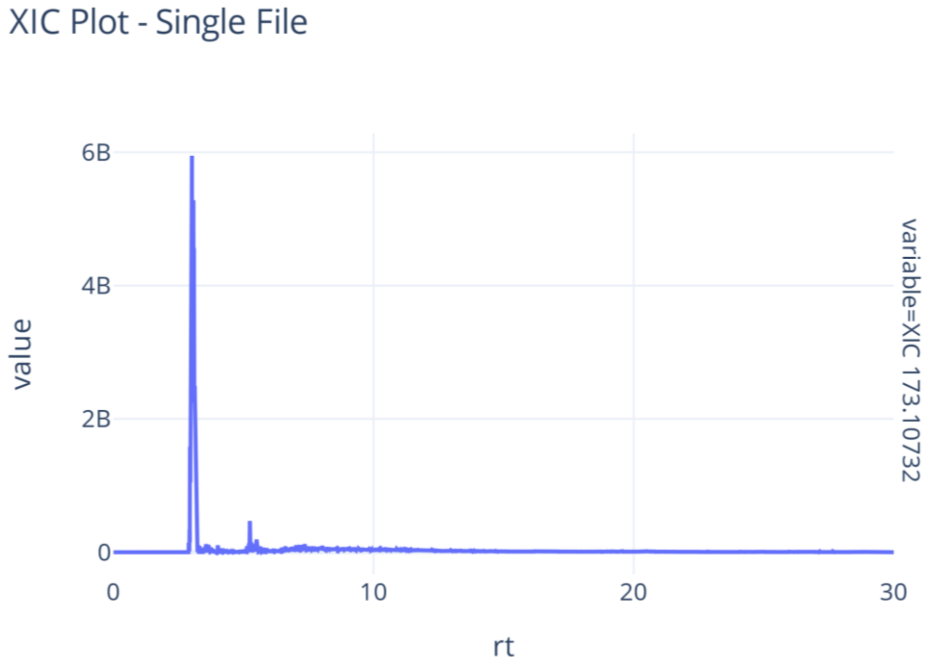

ATA-117-Rd6

*Extracted ion count (**3e**) LC-MS trace of biotransformations of **1e***

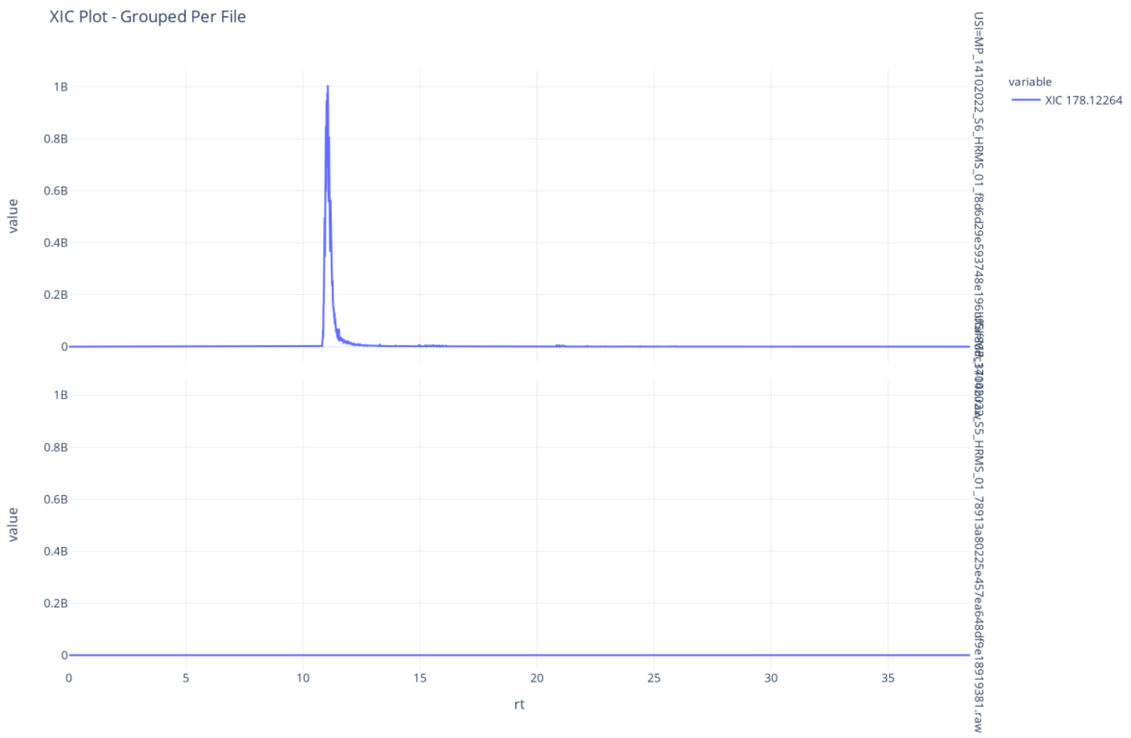

Top: ATA-117-Rd6, Bottom: no enzyme

*Extracted ion count (**3f**) LC-MS trace of biotransformations of **1f***

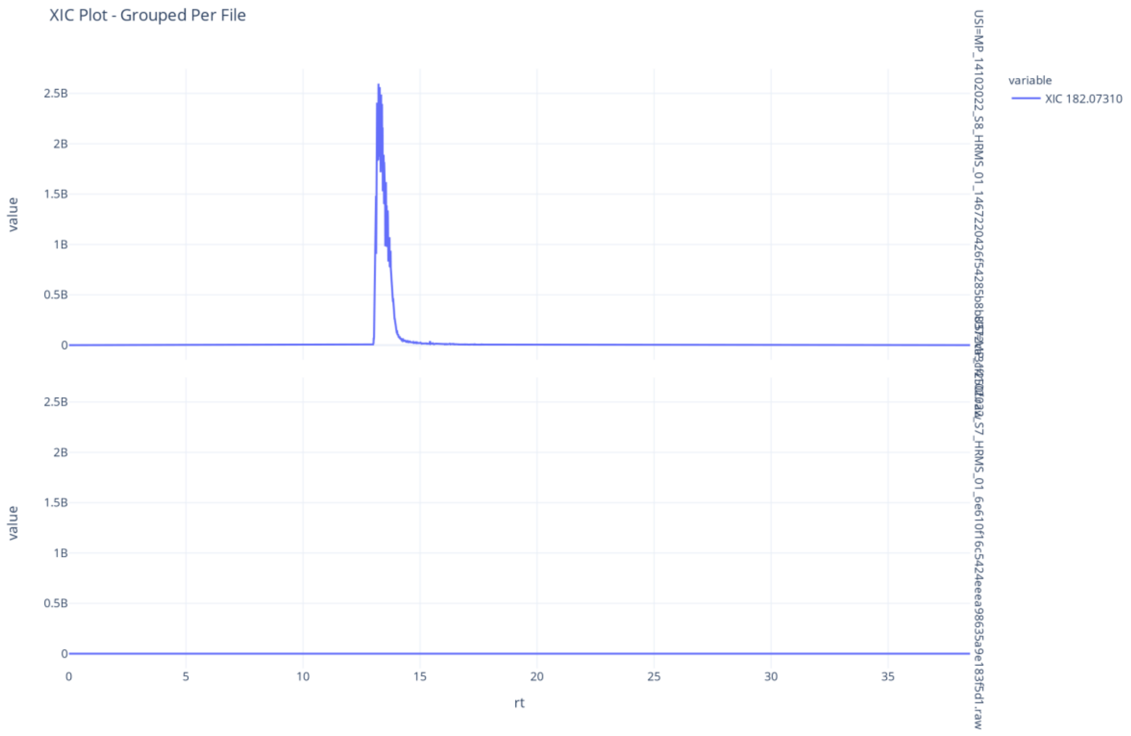

Top: ATA-117-Rd6, Bottom: no enzyme

*Extracted ion count (**3g**) LC-MS trace of biotransformations of **1g***

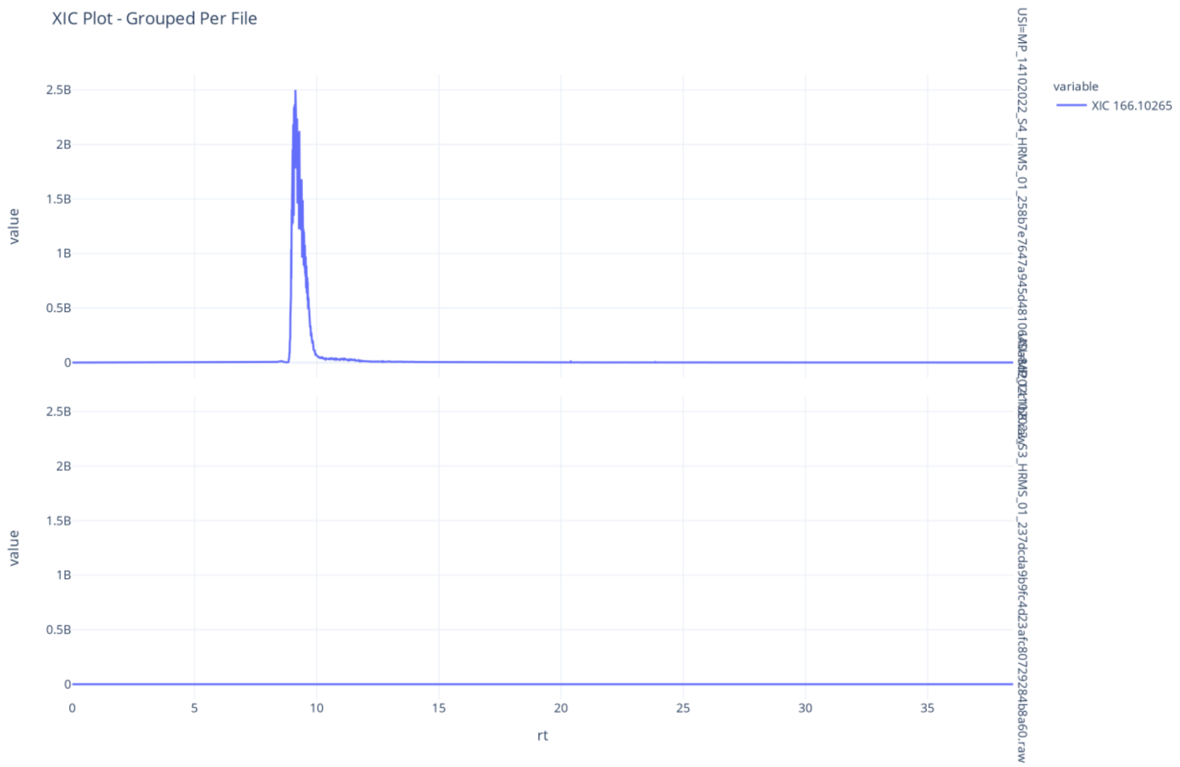

Top: ATA-117-Rd6, Bottom: no enzyme

*Extracted ion count (**3h**) LC-MS trace of biotransformations of **1h***

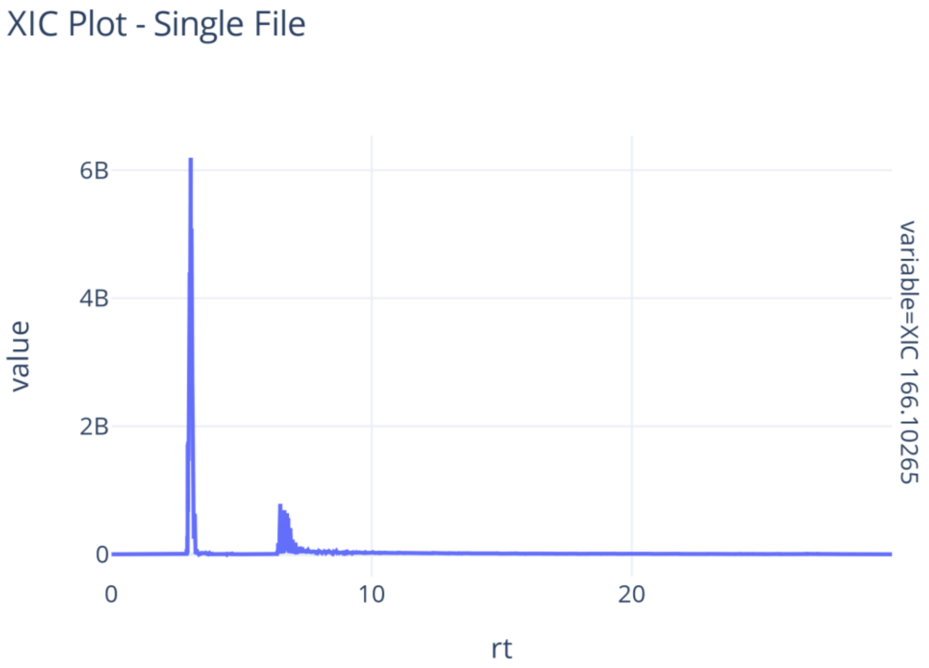

ATA-117-Rd6

*Extracted ion count (**3i**) LC-MS trace of biotransformations of **1i***

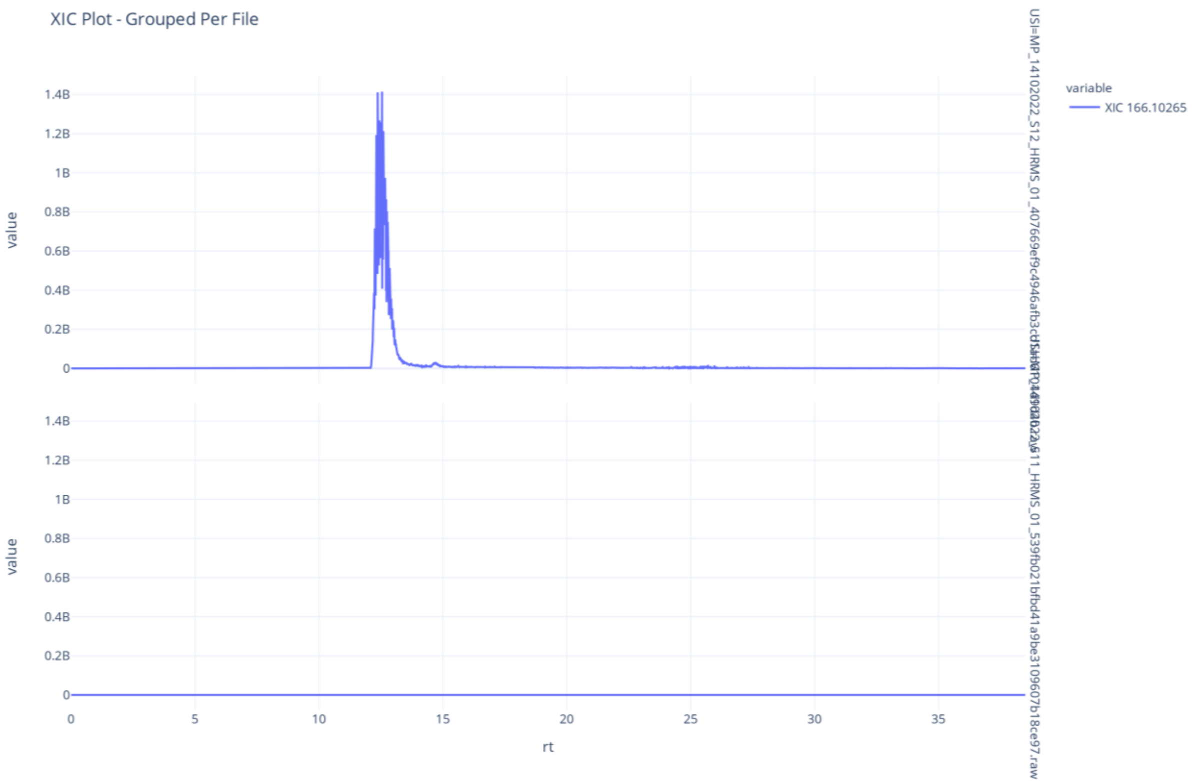

Top: ATA-117-Rd6, Bottom: no enzyme

*Extracted ion count (**3j**) LC-MS trace of biotransformations of **1j***

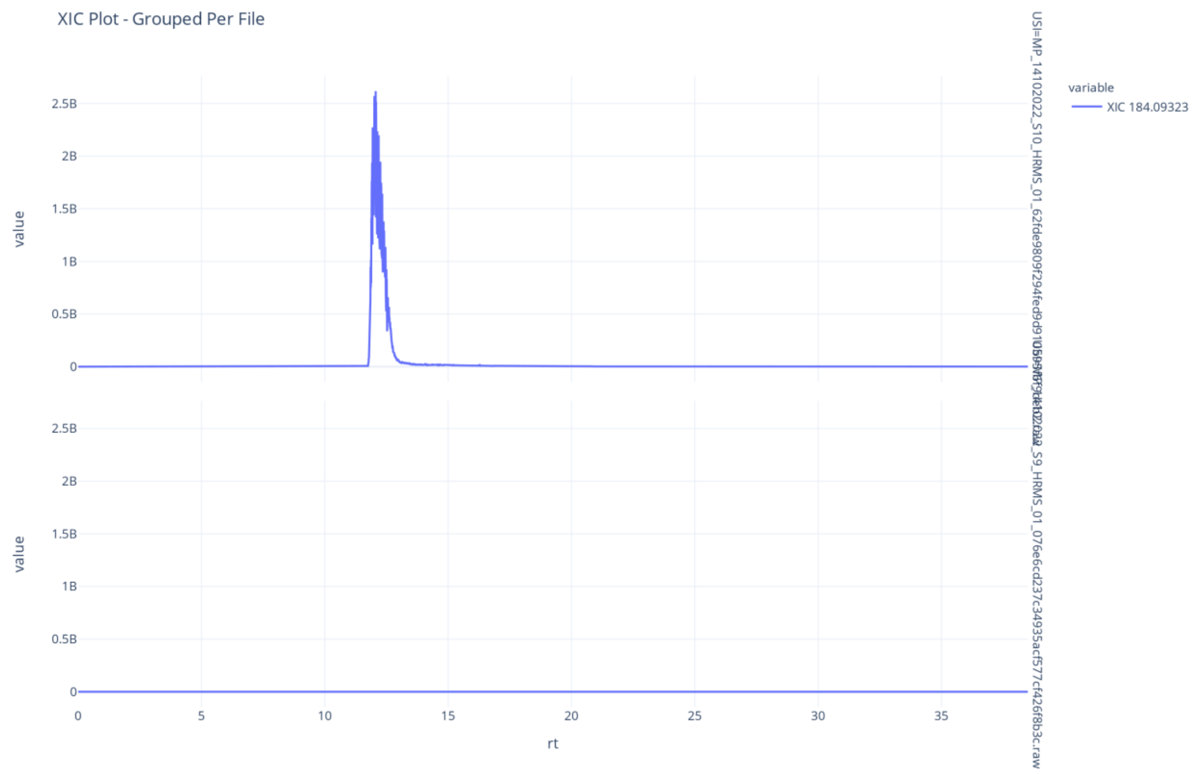

Top: ATA-117-Rd6, Bottom: no enzyme

*Extracted ion count (**3k**) LC-MS trace of biotransformations of **1k***

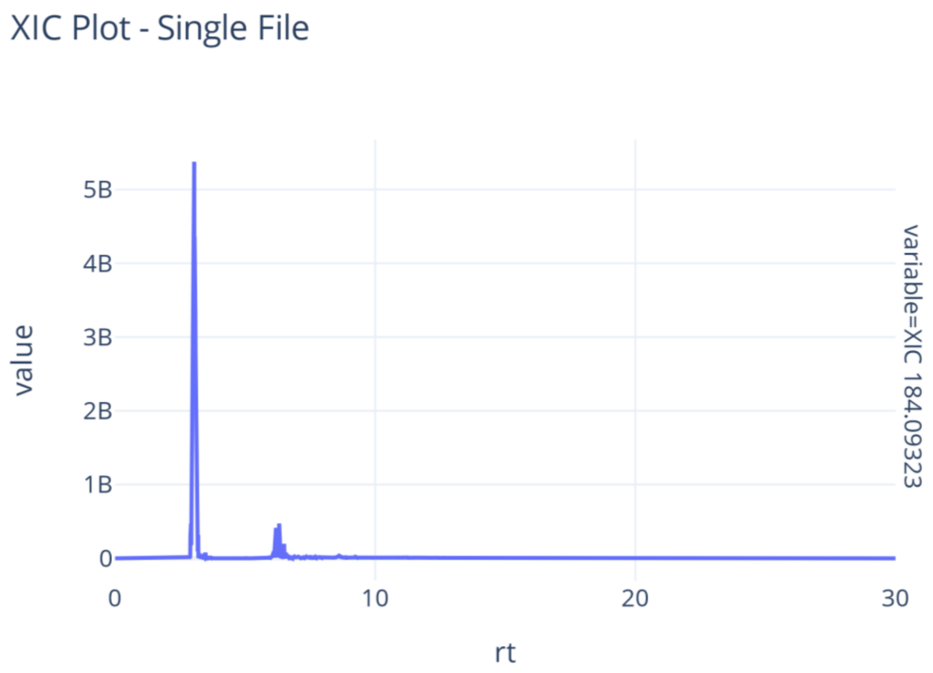

ATA-117-Rd6

*Extracted ion count (**3l**) LC-MS trace of biotransformations of **1l***

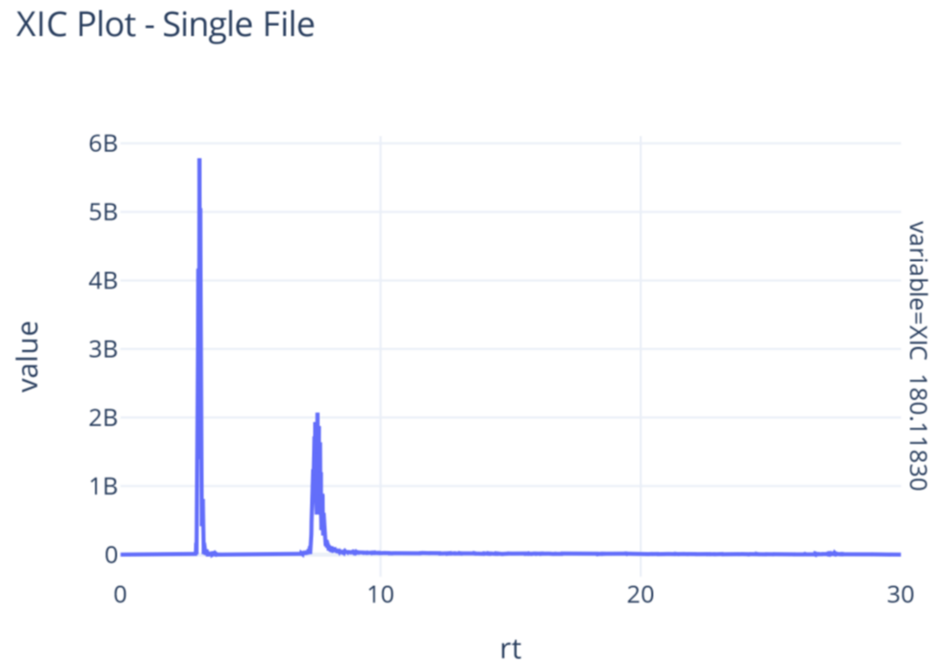

ATA-117-Rd6

*Extracted ion count (**3m**) LC-MS trace of biotransformations of **1m***

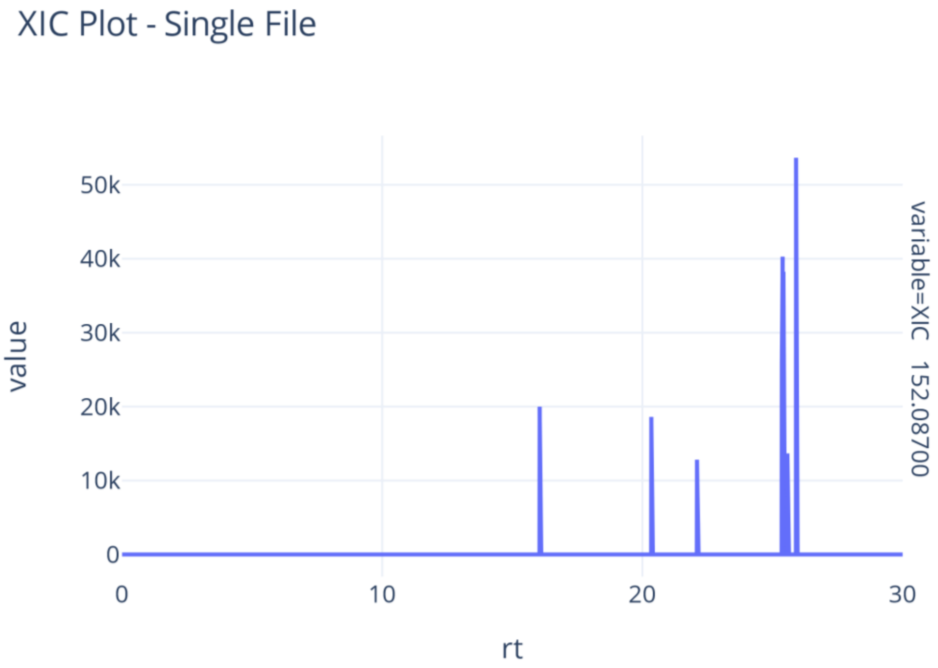

ATA-117-Rd6

*Extracted ion count (ω-isopropylamine-ketone) LC-MS trace of biotransformations of **1m***

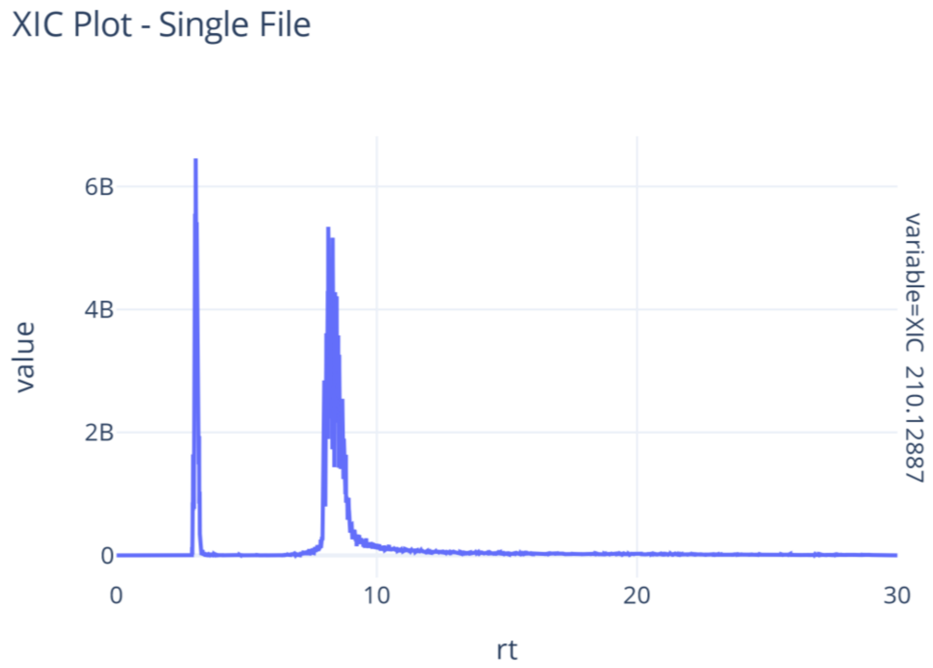

ATA-117-Rd6

*Chiral GC-FID chromatogram of biotransformations of **1a***

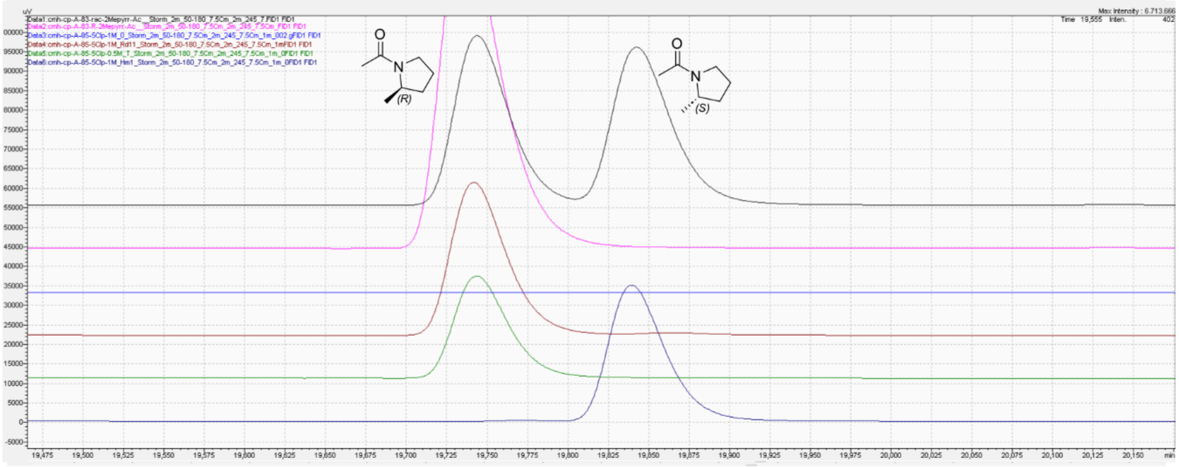

Black: commercial standard of racemic **3a**, pink: commercial standard of (*R*)-**3a**, blue: no enzyme, brown: ATA-117-Rd11, green: *Ts*RTA, dark blue: HEwT W56G

*Chiral GC-FID chromatogram of biotransformations of **1b***

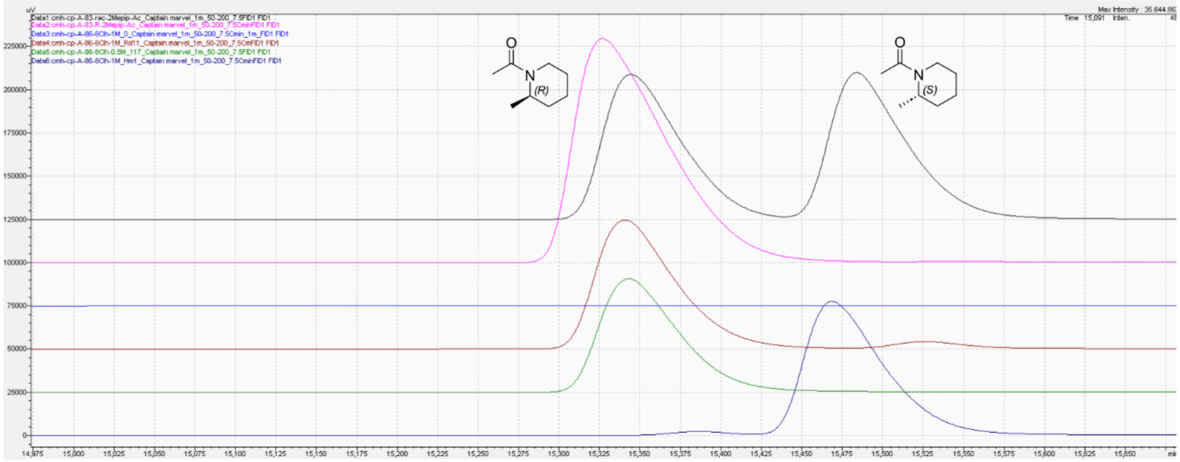

Black: commercial standard of racemic **3b**, pink: commercial standard of (*R*)-**3b**, blue: no enzyme, brown: ATA-117-Rd11, green: *Ts*RTA, dark blue: HEwT W56G

*Chiral GC-FID chromatogram of biotransformations of **1c***

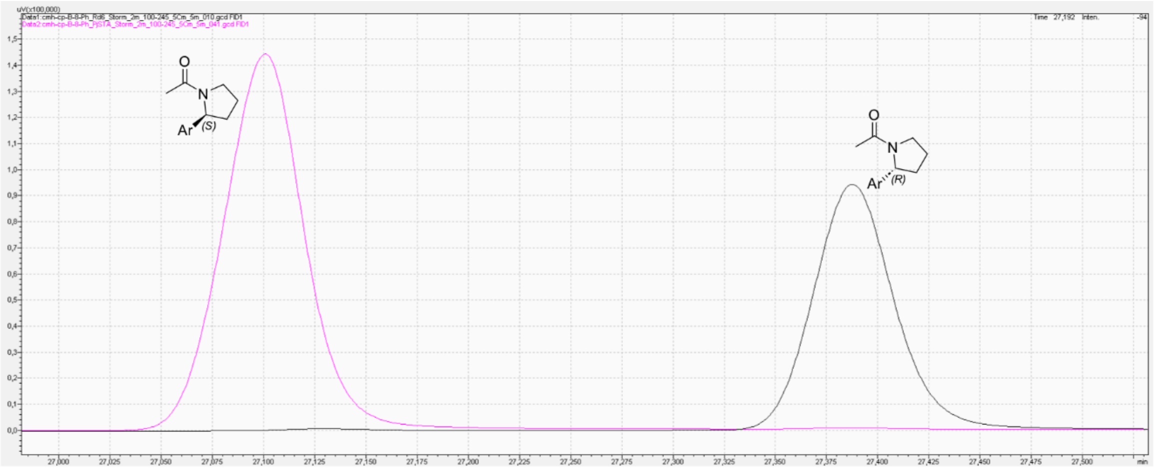

Black: ATA-117-Rd6, pink: *Pj*STA-R6-8

*Chiral GC-FID chromatogram of biotransformations of **1d***

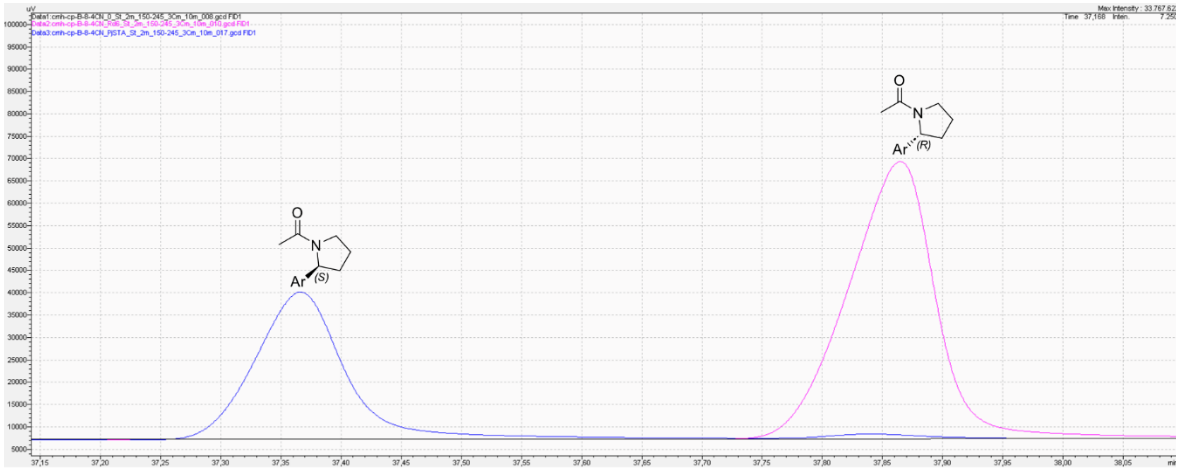

Black: no enzyme, pink: ATA-117-Rd6, blue: *Pj*STA-R6-8

*Chiral GC-FID chromatogram of biotransformations of **1e***

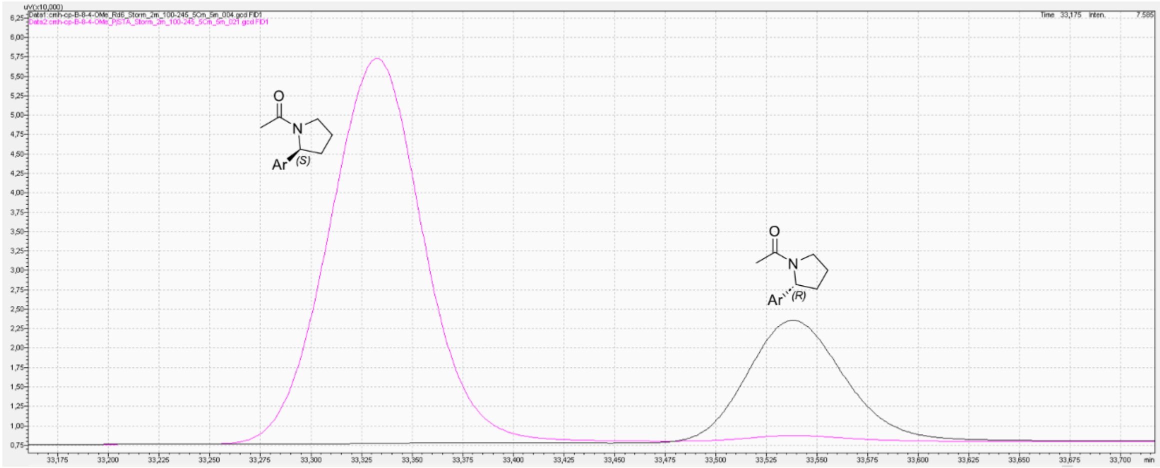

Black: ATA-117-Rd6, pink: *Pj*STA-R6-8

*Chiral GC-FID chromatogram of biotransformations of **1f***

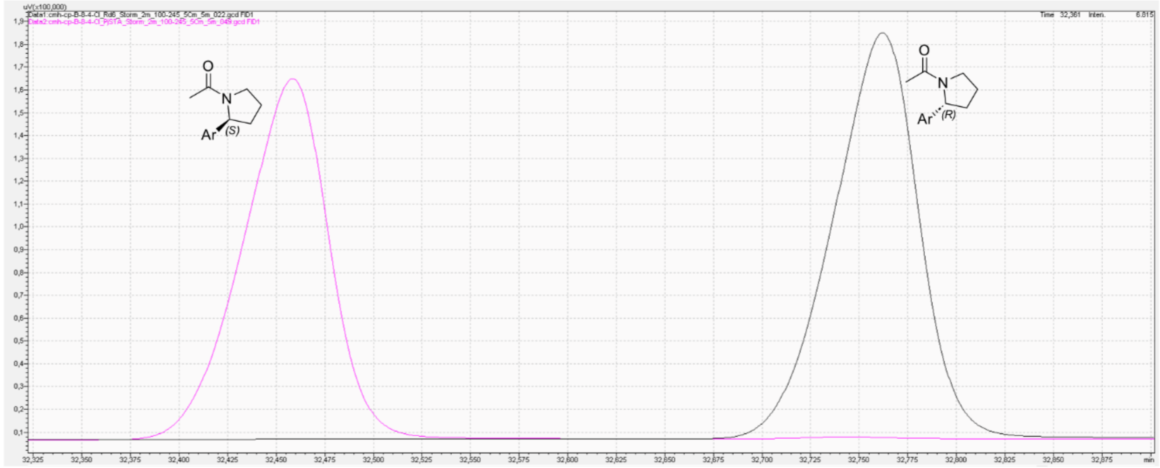

Black: ATA-117-Rd6, pink: *Pj*STA-R6-8

*Chiral GC-FID chromatogram of biotransformations of **1g***

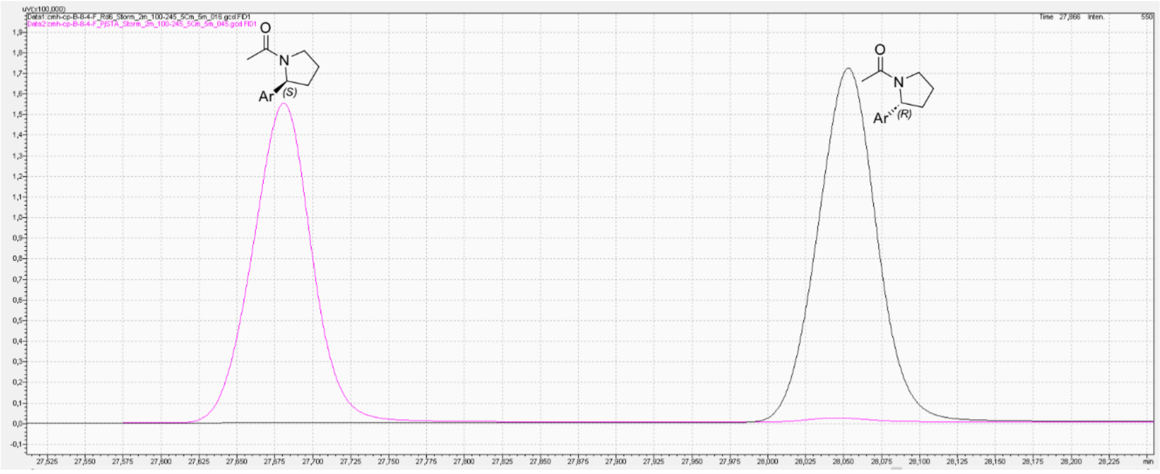

Black: ATA-117-Rd6, pink: *Pj*STA-R6-8

*Chiral GC-FID chromatogram of biotransformations of **1h***

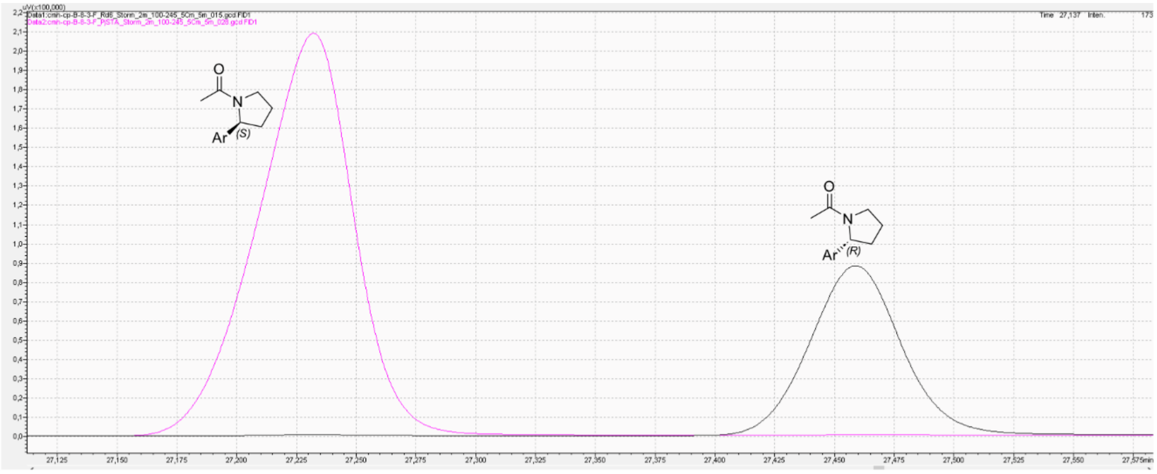

Black: ATA-117-Rd6, pink: *Pj*STA-R6-8

*Chiral GC-FID chromatogram of biotransformations of **1i***

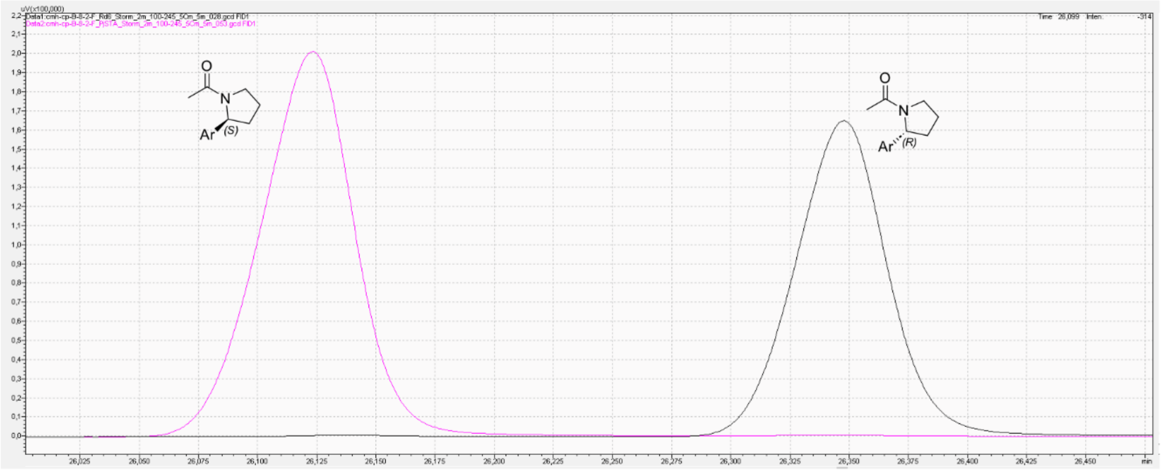

Black: ATA-117-Rd6, pink: *Pj*STA-R6-8

*Chiral GC-FID chromatogram of biotransformations of **1j***

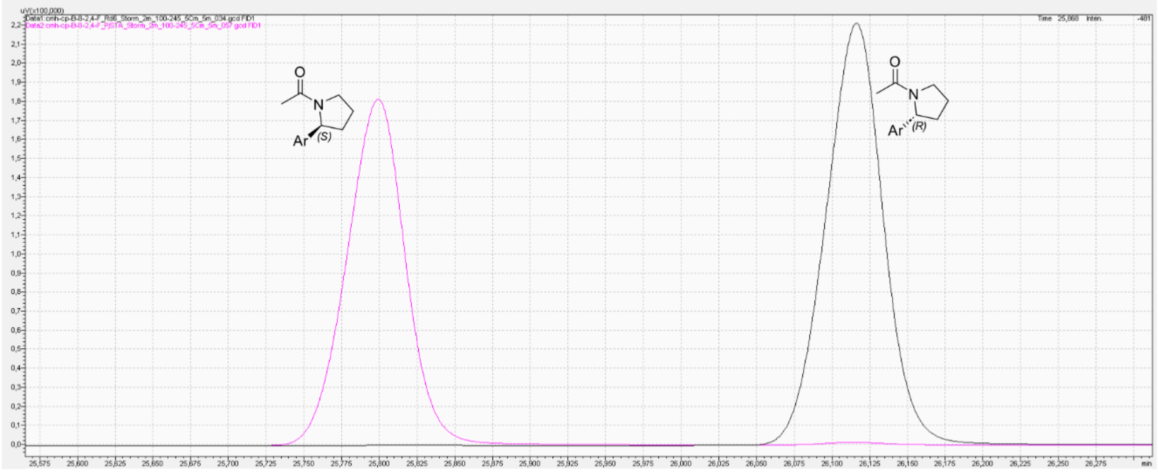

Black: ATA-117-Rd6, pink: *Pj*STA-R6-8

*Chiral GC-FID chromatogram of biotransformations of **1k***

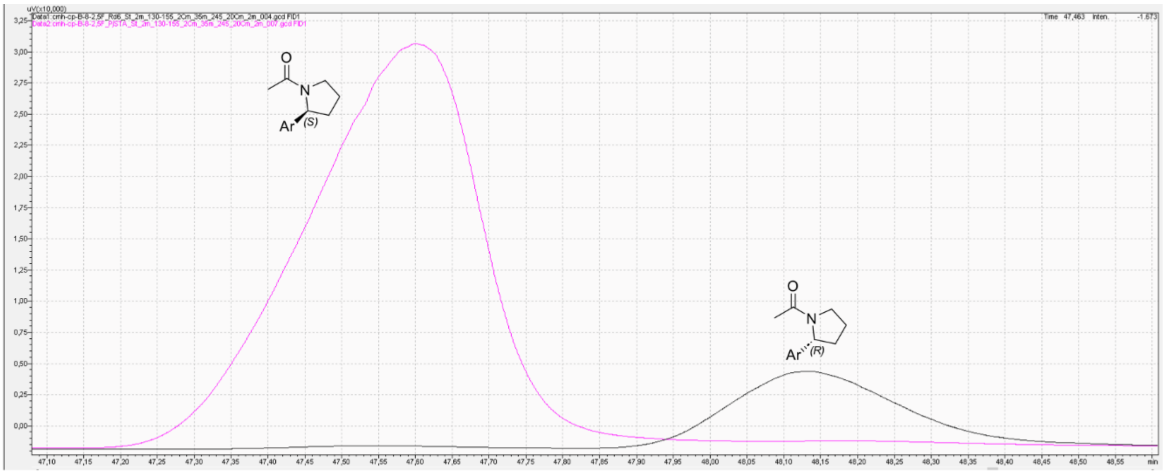

Black: ATA-117-Rd6, pink: *Pj*STA-R6-8

*Chiral GC-FID chromatogram of biotransformations of **1l***

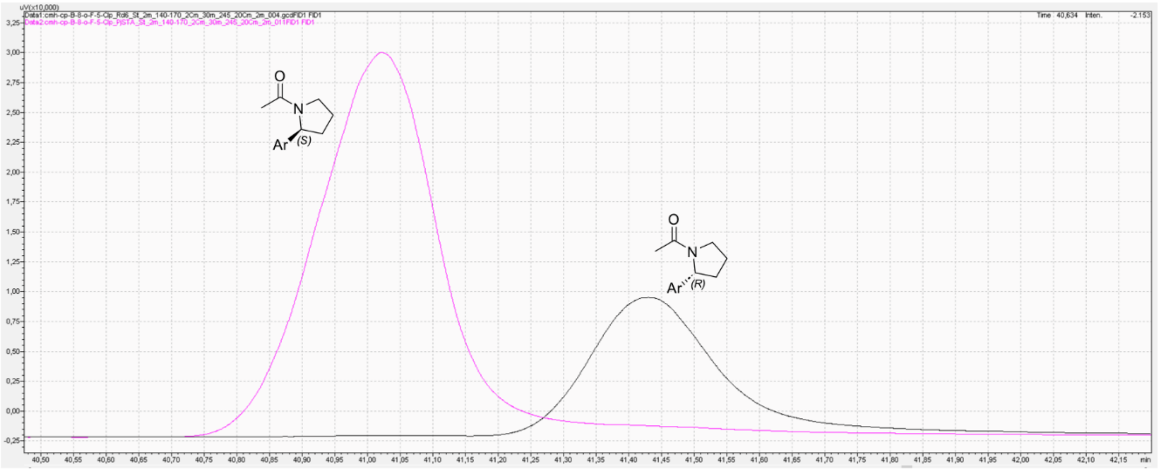

Black: ATA-117-Rd6, pink: *Pj*STA-R6-8

*Chiral GC-FID chromatogram of isolated (R)-**3f** from preparative biotransformation*

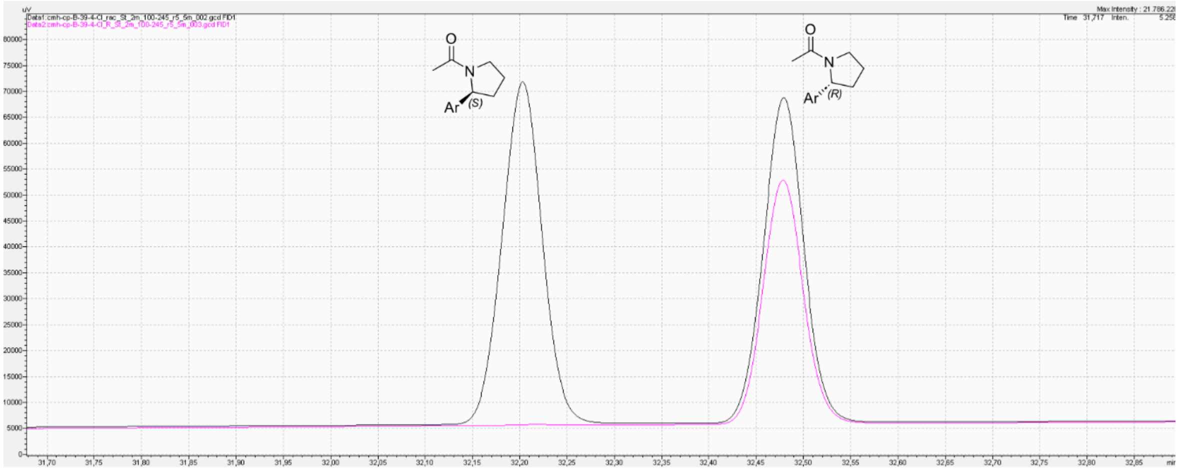

Black: racemic commercial standard of **3f**, pink: isolated (*R*)-**3f** from preparative biotransformation

NMR spectra

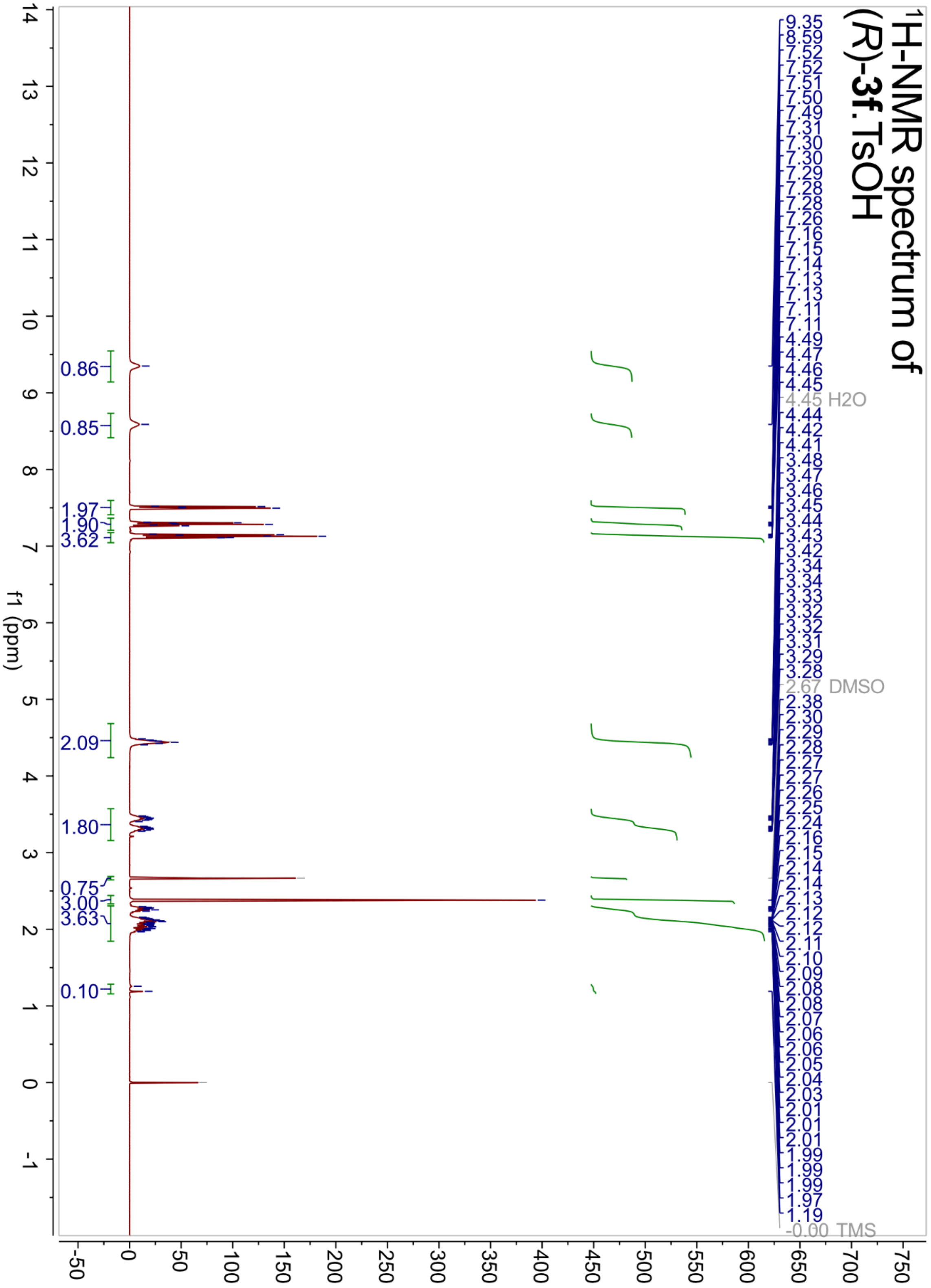

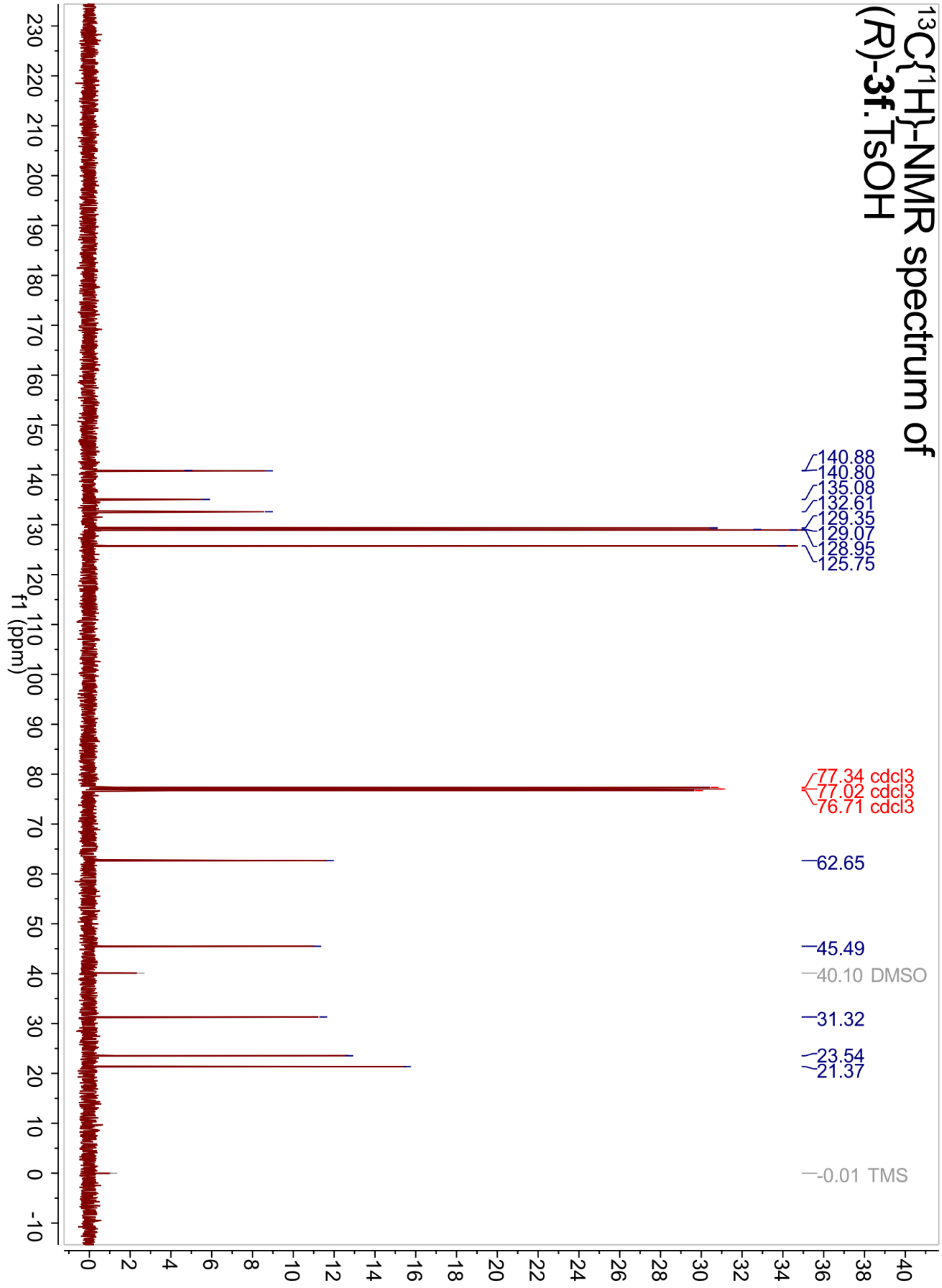

$^{13}C\{^{1}H\}$-NMR spectrum of
(*R*)-**3f**.TsOH
140.88
140.80
135.08
132.61
129.35
129.07
128.95
125.75
77.34 cdcl3
77.02 cdcl3
76.71 cdcl3
62.65
45.49
40.10 DMSO
31.32
23.54
21.37
-0.01 TMS
f1 (ppm)

multiplicity edited HSQC spectrum of (*R*)-**3f**.TsOH

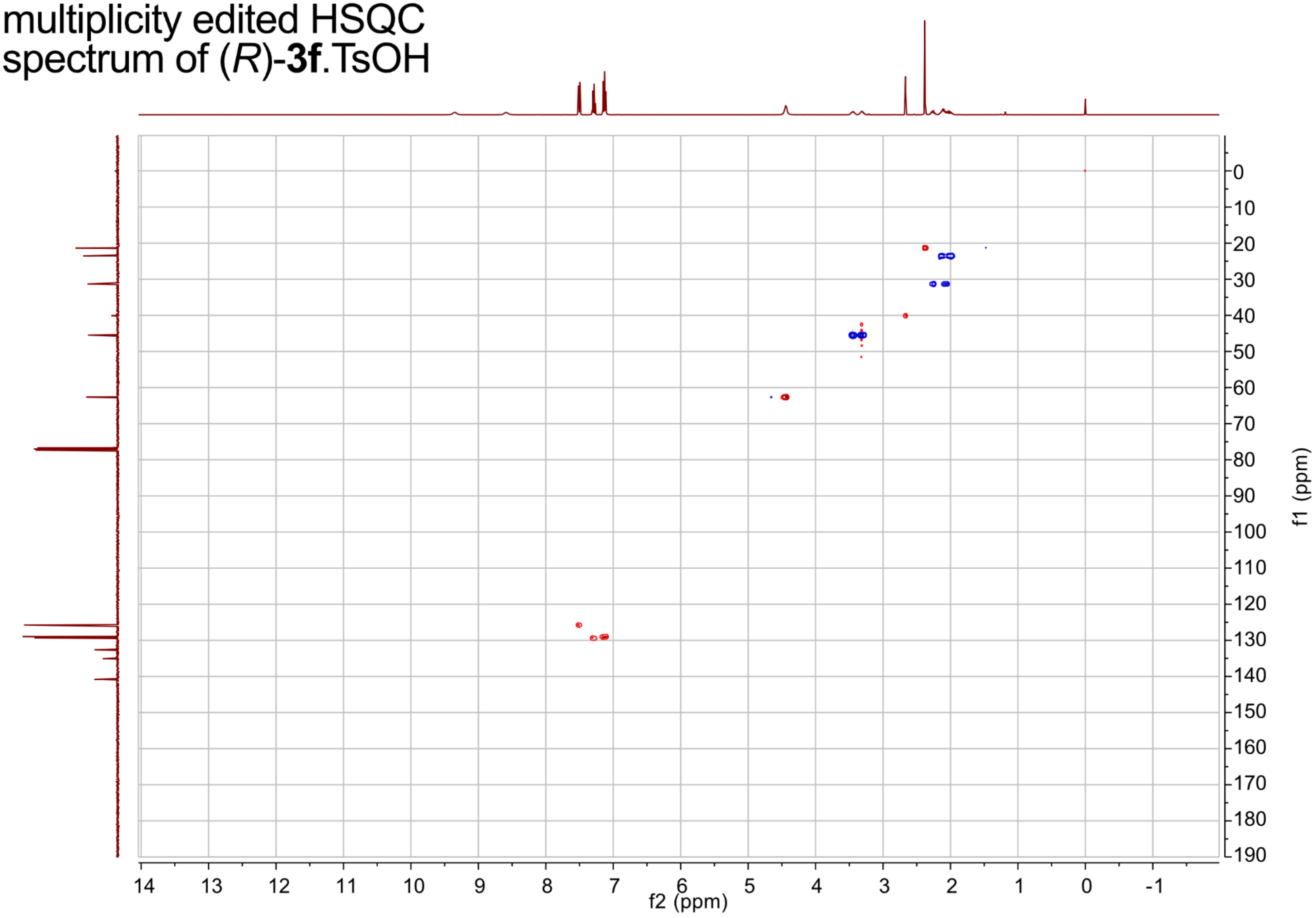

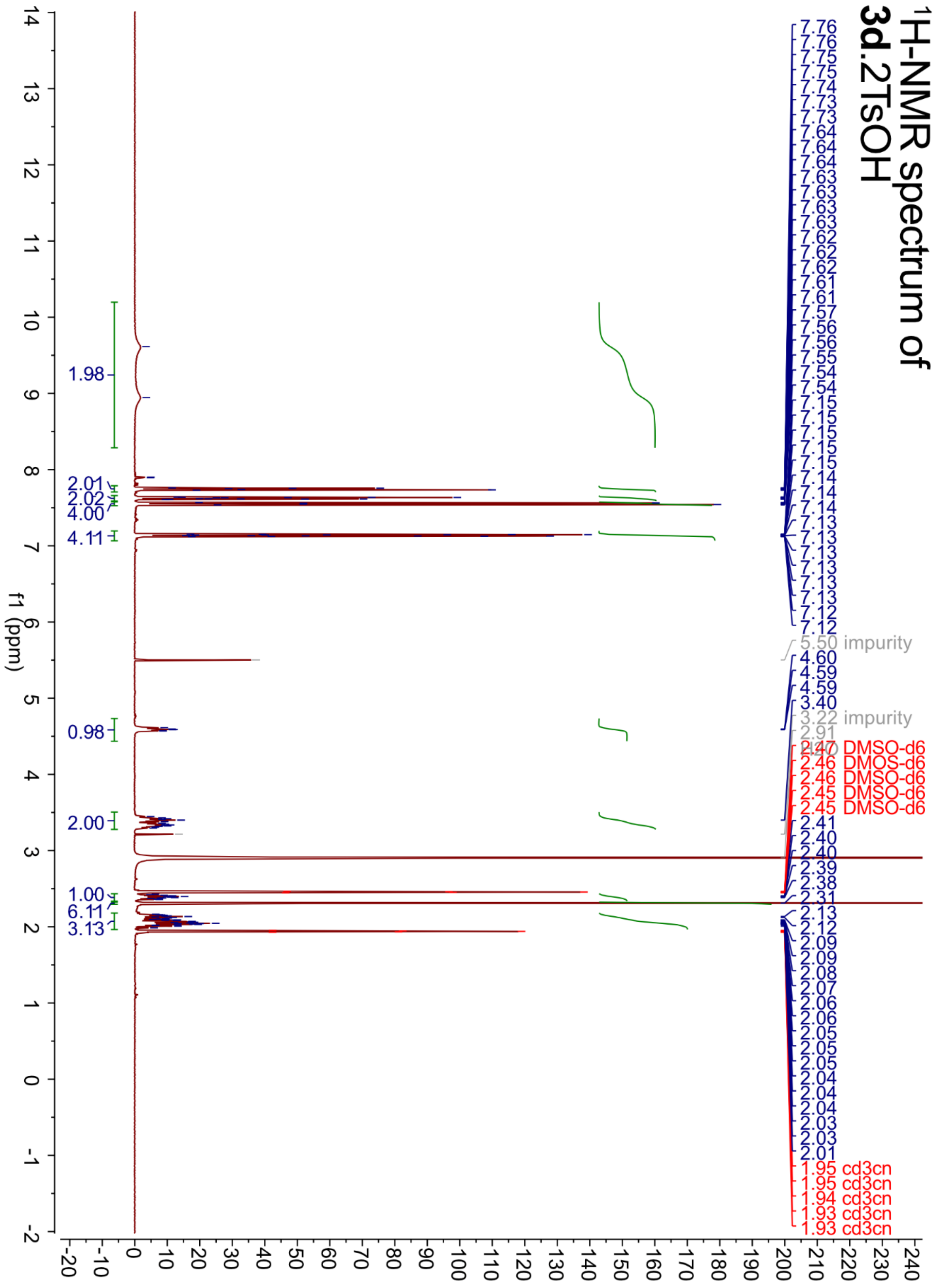
1H-NMR spectrum of
3d.2TsOH
7.76
7.76
7.75
7.75
7.74
7.73
7.73
7.64
7.64
7.64
7.63
7.63
7.63
7.63
7.62
7.62
7.62
7.61
7.61
7.57
7.56
7.56
7.55
7.54
7.54
7.15
7.15
7.15
7.15
7.15
7.14
7.14
7.14
7.13
7.13
7.13
7.13
7.13
7.13
7.13
7.12
7.12
5.50 impurity
4.60
4.59
4.59
3.40
3.22 impurity
2.91
2.47 DMSO-d6
2.46 DMOS-d6
2.46 DMSO-d6
2.45 DMSO-d6
2.45 DMSO-d6
2.41
2.40
2.40
2.39
2.38
2.31
2.13
2.12
2.09
2.09
2.08
2.07
2.06
2.06
2.05
2.05
2.05
2.04
2.04
2.04
2.03
2.03
2.01
1.95 cd3cn
1.95 cd3cn
1.94 cd3cn
1.93 cd3cn
1.93 cd3cn
1.98
2.01
2.02
4.00
4.11
0.98
2.00
1.00
6.11
3.13
f1 (ppm)

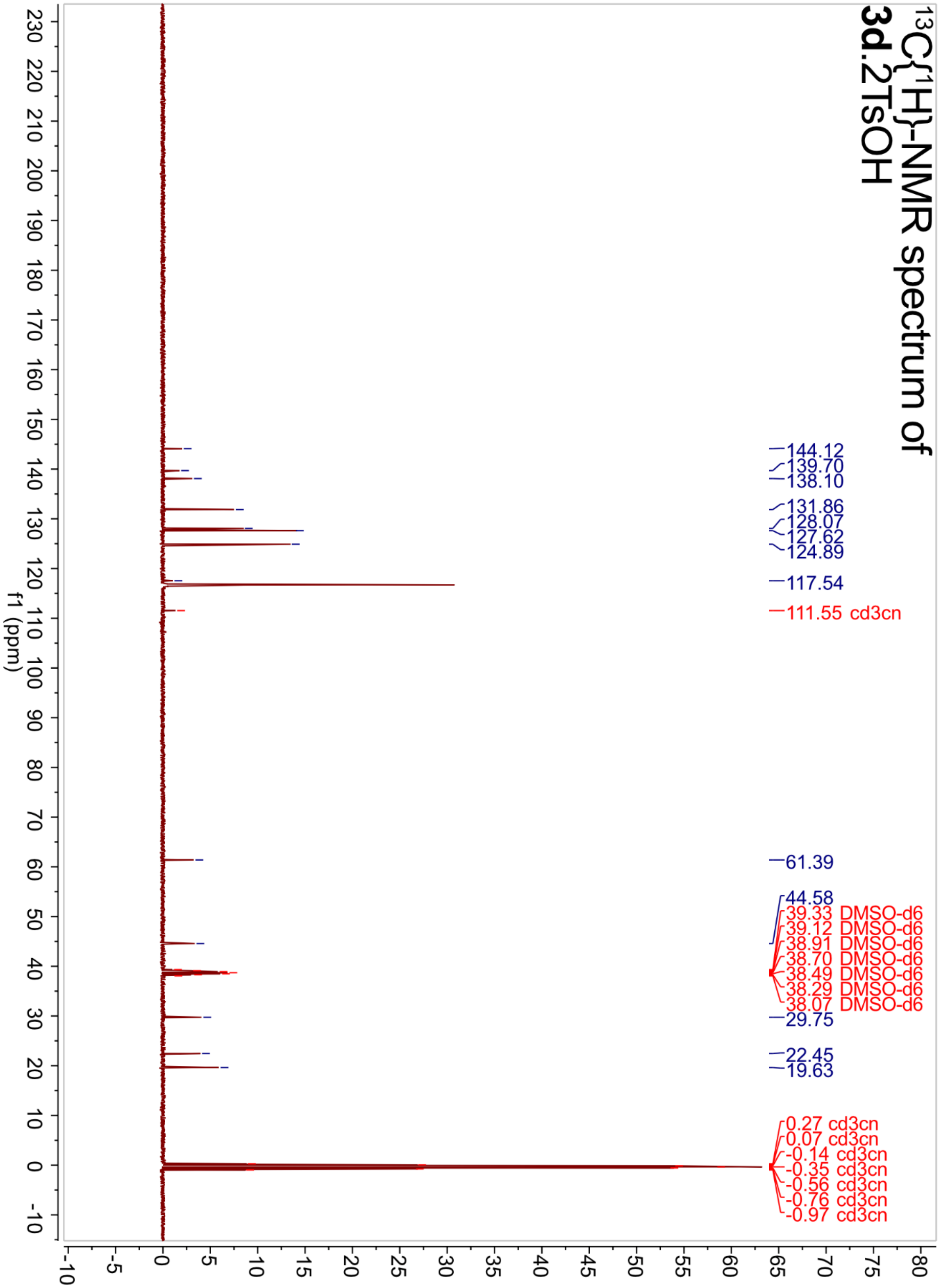

$^{13}C\{^1H\}$-NMR spectrum of
3d.2TsOH
144.12
139.70
138.10
131.86
128.07
127.62
124.89
117.54
111.55 cd3cn
61.39
44.58
39.33 DMSO-d6
39.12 DMSO-d6
38.91 DMSO-d6
38.70 DMSO-d6
38.49 DMSO-d6
38.29 DMSO-d6
38.07 DMSO-d6
29.75
22.45
19.63
0.27 cd3cn
0.07 cd3cn
-0.14 cd3cn
-0.35 cd3cn
-0.56 cd3cn
-0.76 cd3cn
-0.97 cd3cn
f1 (ppm)

multiplicity edited HSQC spectrum of **3d**.2TsOH

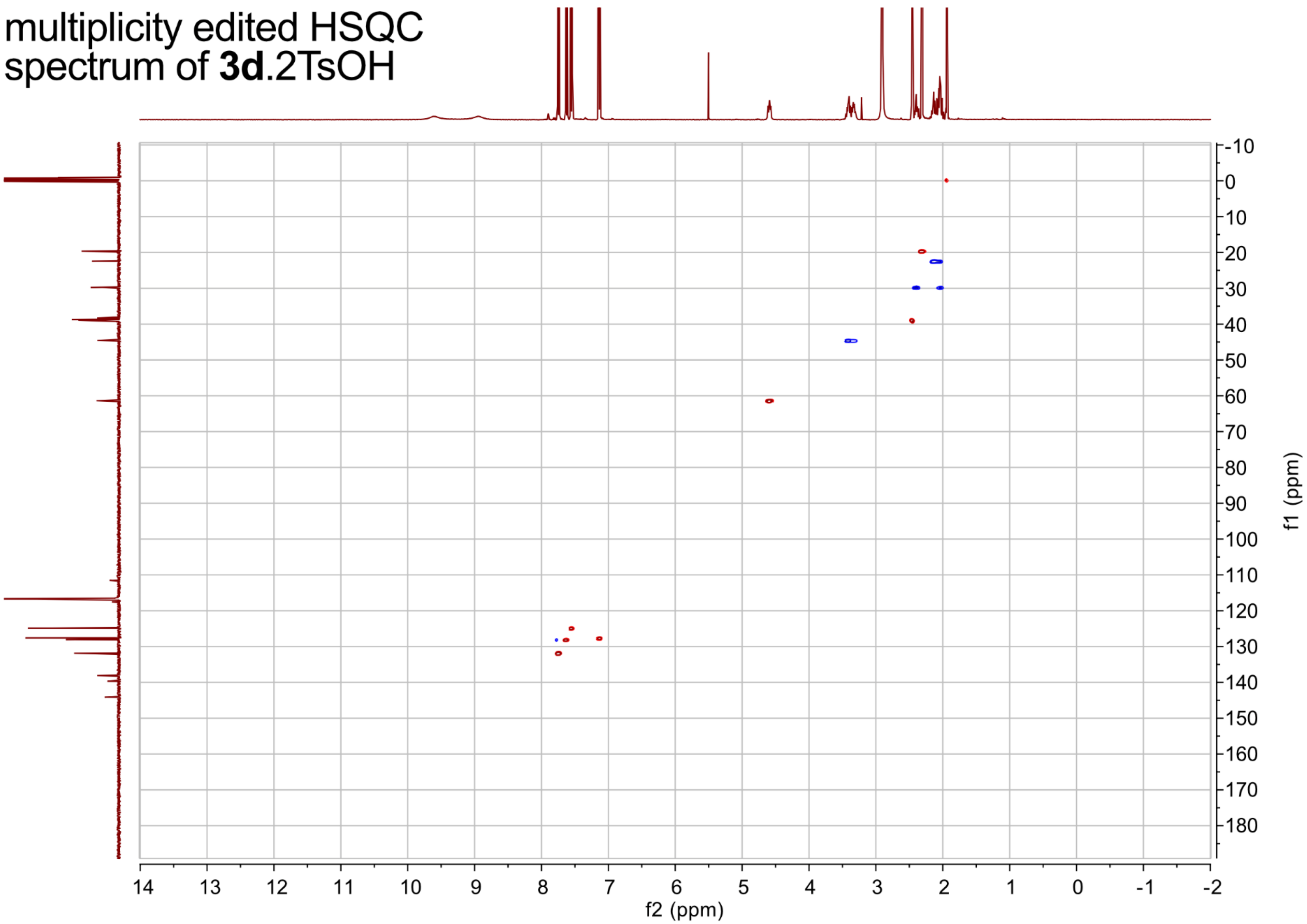

References

(1) Savile, C. K.; Janey, J. M.; Mundorff, E. C.; Moore, J. C.; Tam, S.; Jarvis, W. R.; Colbeck, J. C.; Krebber, A.; Fleitz, F. J.; Brands, J.; Devine, P. N.; Huisman, G. W.; Hughes, G. J. Biocatalytic Asymmetric Synthesis of Chiral Amines from Ketones Applied to Sitagliptin Manufacture. *Science* **2010**, *329*, 305–310. https://doi.org/10.1126/science.1188934.

(2) Heckmann, C. M.; Gourlay, L. J.; Dominguez, B.; Paradisi, F. An (R)-Selective Transaminase From Thermomyces Stellatus: Stabilizing the Tetrameric Form. *Front. Bioeng. Biotechnol.* **2020**, *8*, 707. https://doi.org/10.3389/fbioe.2020.00707.

(3) Höhne, M.; Schätzle, S.; Jochens, H.; Robins, K.; Bornscheuer, U. T. Rational Assignment of Key Motifs for Function Guides in Silico Enzyme Identification. *Nat. Chem. Biol.* **2010**, *6*, 807–813. https://doi.org/10.1038/nchembio.447.

(4) Cerioli, L.; Planchestainer, M.; Cassidy, J.; Tessaro, D.; Paradisi, F. Characterization of a Novel Amine Transaminase from Halomonas Elongata. *J. Mol. Catal. B Enzym.* **2015**, *120*, 141–150. https://doi.org/10.1016/j.molcatb.2015.07.009.

(5) Kaulmann, U.; Smithies, K.; Smith, M. E. B.; Hailes, H. C.; Ward, J. M. Substrate Spectrum of W-Transaminase from Chromobacterium Violaceum DSM30191 and Its Potential for Biocatalysis. *Enzyme Microb. Technol.* **2007**, *41*, 628–637. https://doi.org/10.1016/j.enzmictec.2007.05.011.

(6) Meng, Q.; Ramírez-Palacios, C.; Capra, N.; Hooghwinkel, M. E.; Thallmair, S.; Rozeboom, H. J.; Thunnissen, A.-M. W. H.; Wijma, H. J.; Marrink, S. J.; Janssen, D. B. Computational Redesign of an ω-Transaminase from Pseudomonas Jessenii for Asymmetric Synthesis of Enantiopure Bulky Amines . *ACS Catal.* **2021**, *11*, 10733–10747. https://doi.org/10.1021/acscatal.1c02053.

(7) Pavlidis, I. V.; Weiß, M. S.; Genz, M.; Spurr, P.; Hanlon, S. P.; Wirz, B.; Iding, H.; Bornscheuer, U. T. Identification of (S)-Selective Transaminases for the Asymmetric Synthesis of Bulky Chiral Amines. *Nat. Chem.* **2016**, *8*, 1076–1082. https://doi.org/10.1038/NCHEM.2578.

(8) Contente, M. L.; Planchestainer, M.; Molinari, F.; Paradisi, F. Stereoelectronic Effects in the Reaction of Aromatic Substrates Catalysed by Halomonas Elongata Transaminase and Its Mutants. *Org. Biomol. Chem.* **2016**, *14*, 9306–9311. https://doi.org/10.1039/C6OB01629D.

(9) Studier, F. W. Protein Production by Auto-Induction in High-Density Shaking Cultures. *Protein Expr. Purif.* **2005**, *41*, 207–234. https://doi.org/10.1016/j.pep.2005.01.016.

(10) Schätzle, S.; Höhne, M.; Redestad, E.; Robins, K.; Bornscheuer, U. T. Rapid and Sensitive Kinetic Assay for Characterization of ω-Transaminases. *Anal. Chem.* **2009**, *81*, 8244–8248. https://doi.org/10.1021/ac901640q.

(11) Chambers, M. C.; MacLean, B.; Burke, R.; Amodei, D.; Ruderman, D. L.; Neumann, S.; Gatto, L.; Fischer, B.; Pratt, B.; Egertson, J.; Hoff, K.; Kessner, D.; Tasman, N.; Shulman, N.; Frewen, B.; Baker, T. A.; Brusniak, M. Y.; Paulse, C.; Creasy, D.; Flashner, L.; Kani, K.; Moulding, C.; Seymour, S. L.; Nuwaysir, L. M.; Lefebvre, B.; Kuhlmann, F.; Roark, J.; Rainer, P.; Detlev, S.; Hemenway, T.; Huhmer, A.; Langridge, J.; Connolly, B.; Chadick, T.; Holly, K.; Eckels, J.; Deutsch, E. W.; Moritz, R. L.; Katz, J. E.; Agus, D. B.; MacCoss, M.; Tabb, D. L.; Mallick, P. A Cross-Platform Toolkit for Mass Spectrometry and Proteomics. *Nat. Biotechnol.* **2012**, *30*, 918–920. https://doi.org/10.1038/nbt.2377.

(12) Petras, D.; Phelan, V. V.; Acharya, D.; Allen, A. E.; Aron, A. T.; Bandeira, N.; Bowen, B. P.; Belle-Oudry, D.; Boecker, S.; Cummings, D. A.; Deutsch, J. M.; Fahy, E.; Garg, N.; Gregor, R.; Handelsman, J.; Navarro-Hoyos, M.; Jarmusch, A. K.; Jarmusch, S. A.; Louie, K.; Maloney, K. N.; Marty, M. T.; Meijler, M. M.; Mizrahi, I.; Neve, R. L.; Northen, T. R.; Molina-Santiago, C.; Panitchpakdi, M.; Pullman, B.; Puri, A. W.; Schmid, R.; Subramaniam, S.; Thukral, M.; Vasquez-Castro, F.; Dorrestein, P. C.; Wang, M. GNPS Dashboard: Collaborative Exploration of Mass Spectrometry Data in the Web Browser. *Nat. Methods* **2022**, *19*, 134–136. https://doi.org/10.1038/s41592-021-01339-5.

(13) Waterhouse, A.; Bertoni, M.; Bienert, S.; Studer, G.; Tauriello, G.; Gumienny, R.; Heer, F. T.; de Beer, T. A. P.; Rempfer, C.; Bordoli, L.; Lepore, R.; Schwede, T. SWISS-MODEL: Homology Modelling of Protein Structures and Complexes. *Nucleic Acids Res.* **2018**, *46*, W296–W303. https://doi.org/10.1093/nar/gky427.

(14) Guthrie, J. P.; Pitchko, V. Hydration of Carbonyl Compounds, an Analysis in Terms of No Barrier Theory: Prediction of Rates from Equilibrium Constants and Distortion Energies. *J. Am. Chem. Soc.* **2000**, *122*, 5520–5528. https://doi.org/10.1021/ja992991q.
